# From flat to twisted – multifunctional phosphacyclic nanocarbons based on Vat Orange 3†‡

**DOI:** 10.1039/d4sc07106a

**Published:** 2025-01-28

**Authors:** Reza Dadgaryeganeh, Jesse LeBlanc, Ekadashi Pradhan, Dandan Miao, Amaar Hussein, Howard N. Hunter, Tao Zeng, Carlos Romero-Nieto, Thomas Baumgartner

**Affiliations:** a Department of Chemistry, York University 4700 Keele St Toronto ON M3J 1P3 Canada; b Facultad de Farmacia, Universidad de Castilla-La Mancha Calle Almansa 14, Edificio Bio-Incubadora Albacete 02008 Spain tzeng@yorku.ca carlos.romeronieto@uclm.es tbaumgar@yorku.ca

## Abstract

The field of π-conjugated organic materials has seen significant advances in recent years. However, enhancing the functionality of well-established, mass-produced compounds remains a considerable challenge, despite being an intriguing strategy for designing high-value organic materials with low production costs. In this context, vat dyes, known for their wide range of colors and extensive use in the textile industry are particularly attractive. Here, we present an innovative approach that conjoins phosphorus heterocycles with the dye Vat Orange 3 (VO3) to yield novel nanocarbons with enhanced functional properties. X-ray crystallography reveals distinct twisting of the scaffold in the solid state, while the modification of the phosphorus centers leads to intriguing and versatile photophysics. Thin-film analyses show unusual, pronounced emission features that switch from green to orange upon aggregation. Furthermore, Lewis-adduct formation induces a fluorescence redshift upon coordination to the phosphorus moiety and cyclic voltammetry confirms the acceptor character of the system. This work demonstrates the versatility of phosphorus-modified vat dyes as value-added organic compounds and paves the way for the development of new functional 2D nanocarbons with broad technological relevance.

## Abstract 2

Core-expansion of the commercial dye Vat Orange 3 with two P-containing fused rings unlocks a multitude of tunable properties that are not present in the original dye, paving the way for practical application of these value-added nanocarbons.

## Graphical abstract

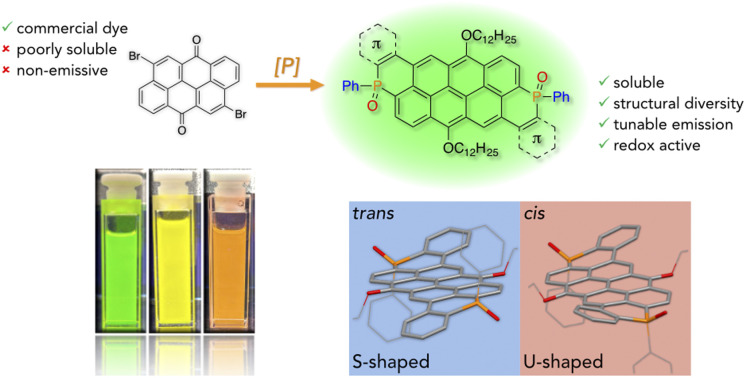

## Introduction

The last two decades have witnessed accelerating development of π-conjugated organic materials with the goal of replacing metals and other limited resources for their negative environmental impact in practical applications.^1,2^ Many π-conjugated molecular systems have led to organic devices with satisfactory performances, however, more research is required to establish this technology widely.^3–5^ Key parameters to enhance the value of novel organic materials are, among others, the quality of interaction between the extended π-systems and visible light, ease of gaining or losing electrons, and/or conduction of ionic charges through their bulk phases. The latter requires optimized molecular packing in the solid state.^6^ Thus, to be able to exploit the full potential of organic materials in practical devices, novel molecular structures need to be designed that allow: (1) effectively modulating the electronic structure of the systems to attain desirable functional properties and (2) adjusting the solid-state interactions for effectively optimizing the supramolecular organization in the bulk.

In this context, nanocarbons such as nanographenes, graphene nanoribbons (GNRs), and 2-dimensional (2D) polycyclic aromatic hydrocarbons (PAHs) are intriguing cores with desirable photophysical and semiconducting properties for organic electronics.^7–9^ In addition, recent studies have shown that structural ‘anomalies’ such as non-hexagonal rings, *i.e.*, five-, seven-, or eight-membered carbo- and/or heterocycles^10–16^ can unlock intriguing structural diversity (*i.e.*, twisted *vs.* planar graphenic scaffolds) and enable the exploitation of unique electronic properties not present in smaller systems (*e.g.*, effectively tunable frontier molecular orbitals and energy gaps).^17–20^

Two fundamental approaches for the preparation of new nanocarbons with improved capabilities include: (a) the bottom-up synthesis from thoroughly designed molecular architectures or (b) innovative derivatization of industrially produced dyes on large scales. While the former strategy has indeed led to outstanding molecular architectures, it often suffers from tedious synthetic pathways with long production times and high costs. The latter approach has the advantage of starting from economically accessible raw materials, which through straightforward synthetic routes, enable cost-effective preparation. However, the disadvantage of this approach is that providing improved capabilities to well-established molecular architectures is often an arduous task.

**Fig. 1:**
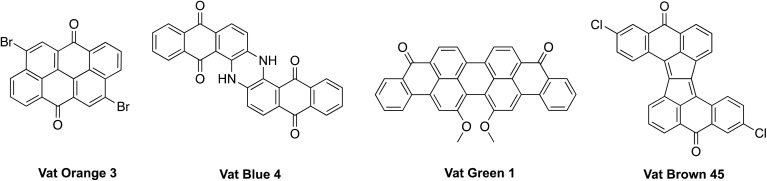
Representative vat dyes.

In this context, vat dyes are a particularly interesting family of organic molecules (Fig. 1).^21^ Their production is estimated to be in the range of 50–100,000 tons per year^22^ accounting for approximately 5–10% of the total textile dye market.^23^ Vat dyes comprise extended π-systems with intense UV-vis absorption, however, they are inherently insoluble due to strong π–π interactions, in addition to being mostly non-emissive. Nevertheless, owing to their absorption features, vat dyes cover a variety of colors and their massive volumes lower the production cost considerably.^24,25^ Moreover, the presence of nanocarbon units already within the vat dye core (*e.g.*, anthanthrene, rylene) that are otherwise difficult to access due to onerous and expensive synthetic protocols, constitutes a key asset for the effective elaboration of functional materials. In addition, the presence of keto, halogen, and (occasionally) amine groups make them attractive precursor scaffolds.^26–28^ Examples of vat-dye functionalization toward improved functional properties have been recently reported, revealing potential applicability in organic photovoltaics (OPVs), organic light-emitting devices (OLEDs), and organic field-effect transistors (OFETs).^29–35^ However, there still remains a need for comprehensive research to uncover their full potential. In this context, Vat Orange 3 (VO3, Fig. 1) is a particularly interesting congener.^36–40^ It features an anthanthrene core with strategically placed functional groups – keto and bromo – that can be leveraged for functionalization toward enhanced properties; *i.e.*, solubility, electronic properties, luminescence, *etc.*

We hypothesized that synergistically embedded heterocycles with inorganic main group elements, such as B, Si, and P, would enable the modulation of the frontier molecular orbital energy levels due to their distinct intrinsic electronics. Simultaneously, the molecular packing in the bulk phase could be also controlled.^13,41^ Among the most suitable main group elements, phosphorus provides several desirable electronic and geometrical characteristics for improved functions through simple chemistry avenues.^42^ For example, several phosphorus-containing conjugated materials with efficient luminescence or pronounced electron-accepting abilities have been reported.^42–48^ Notably, the tetrahedral geometry of the phosphorus moieties also enables their use as stable chiral centers.^49^ Thus, embedding two phosphorus centers in a π-conjugated scaffold gives rise to two stereoisomers (*cis* and *trans*) that can be leveraged for the modulation of the supramolecular organization in the solid state, while maintaining the photophysics and electronics specific to the π-conjugated core.^6^ Despite the advantages that main group elements, and especially P-containing conjugated systems, provide for the development of improved functional materials,^13–16,48,50^ their influence on the properties of vat dyes remains largely unexplored. Given the well-established benefits of phosphorus in enhancing the electronic properties of smaller carbon-based systems, the design and synthesis of novel 2D-expanded π-conjugated frameworks incorporating phosphorus present a promising area for further investigation.

Herein, we showcase the design and development of a first-of-its-kind series of highly functional, π-extended VO3 materials. Through an innovative derivatization strategy, we have fused two six-membered phosphorus heterocycles with the VO3 core, adding significant value to the overall properties of vat dyes. In particular, our comprehensive structure–property study reveals that the incorporation of the phosphorus-based units to the vat dye core leads to (i) an unprecedented class of 2D-extended nanocarbons containing phosphorus heterocycles that are soluble and have highly tunable optical and electronic properties, (ii) intriguing behavior in films, and iii) the formation of P-based stereoisomers with distinct solid-state packing. Our study thus provides a deeper understanding of the overall impact of phosphorus heterocycles in large, 2D-conjugated nanocarbon scaffolds.

## Results and discussion

### Synthesis and characterization

**Scheme 1:**
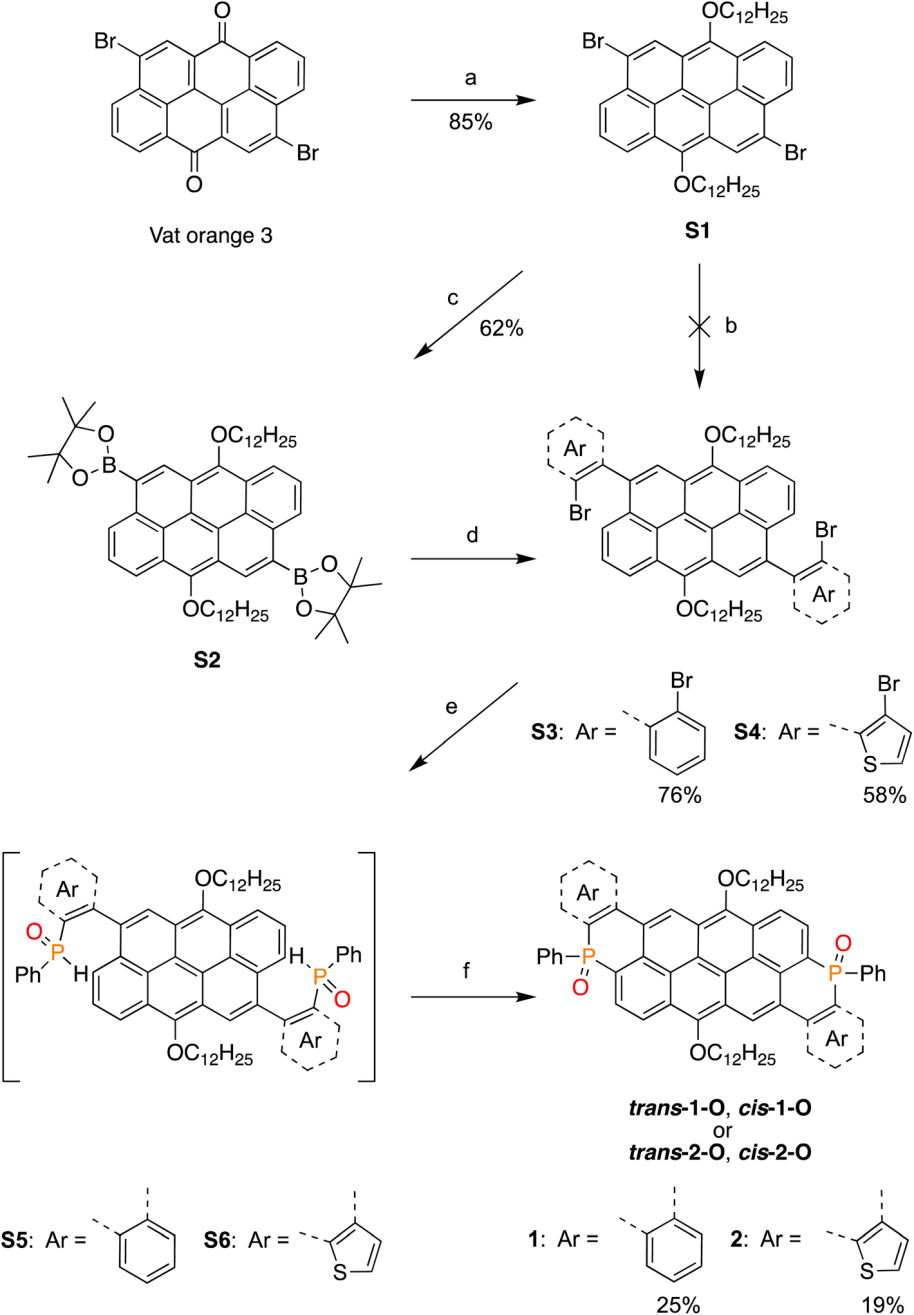
Synthetic routes toward 1-O, and 2-O as mixtures of *cis* & *trans* isomers. (a) Dodecyl bromide, sodium dithionite, 0.1 M NaOH, 70 °C, 3 h. (b) K_2_CO_3_, 3-bromo-2-borapinacolatothiophene, Pd(PPh_3_)_4_, 1,4-dioxane, 100 °C, 20 h. (c) (Bpin)_2_, Pd(dppf)Cl_2_, KOAc, dioxane, 100 °C, 20 h. (d) 2,3-Dibromothiophene (or 1,2-dibromobenzene), Pd(PPh_3_)_4_, K_2_CO_3_, Dioxane/H_2_O: 3 : 1, 100 °C, 20 h. (e) 1: *n*-BuLi, PhPCl_2_, THF, −78 °C, 4 h; 2: H_2_O, 1 h. (f) AgNO_3_, CH_3_CN/Toluene: 1 : 1, 90 °C, 12 h.

The synthetic route toward targets 1-O and 2-O is illustrated in Scheme 1. To alleviate the well-known poor solubility of vat dyes due to strong π-stacking interactions induced by the planar conjugated units, we first converted VO3 to S1 in 85% yield. Installing the 2-bromoaryl units on S1 could, in principle, be realized through Suzuki cross-coupling with the corresponding borylated reagent. However, despite several attempts and varied reaction conditions, S4 did not form.

Instead, cross-coupling was accomplished by Miyaura borylation *via*S2 and the subsequent reaction with the corresponding dibromoaryl derivatives to afford S3 and S4 in 76% and 58% yield, respectively. Finally, the phosphoryl groups were incorporated by metal–halogen exchange with *n*-BuLi and subsequent addition of PhPCl_2_. Hydrolysis of the remaining P–Cl bond with water then led to the intermediate S5 (or S6) after Michaelis-Arbuzov rearrangement (see Scheme S1‡). Treatment of the crude mixtures of S5 or S6 with a catalytic amount of AgNO_3_ led to both 1-O and 2-O as a mixture of *cis*- and *trans*-isomers in 25 and 19% overall yields for reactions *e* and *f* (Scheme 1), respectively.^51^ The two isomers of 1-O are each characterized by a singlet in their ^31^P-NMR spectra at 9.2 and 8.8 ppm for *trans*-1-O and *cis*-1-O, whereas the isomers for 2-O feature corresponding singlets at 7.8 and 7.5 ppm for *trans*-2-O and *cis*-2-O, respectively. Notably, all derivatives are soluble in chloroform and dichloromethane, with accessible concentrations of 1.2 × 10^−2^ M, and 5 × 10^−4^ M, respectively, and present outstanding air- and photo-stability. Moreover, due to the pronounced polarity difference stemming from the orientation of the P=O groups for both the benzo- and thieno-fused species, the *cis*- and *trans*-isomers were separable *via* column chromatography (see ESI‡ for details).

**Scheme 2:**
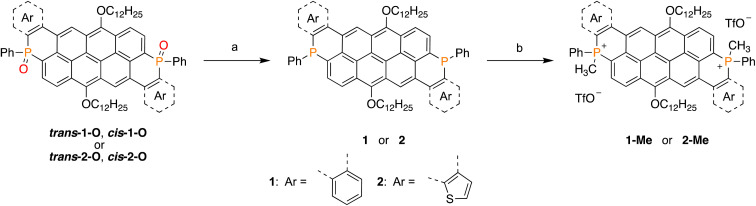
P-modification reactions of 1-O and 2-O toward 1 and 2 and further modification toward 1-Me and 2-Me, both as mixtures of isomers (inseparable); (a) HSiCl_3_, THF, MW, 150 °C, 20 min. (b) MeOTf, CH_2_Cl_2_, 0 °C → RT, 12 h.

The presence of the phosphorus atoms within the vat frameworks provides access to a larger family of differently P-functionalized species. This, however, requires a starting material with trivalent phosphorus atoms (Scheme 2). Thus, 1-O and 2-O were treated with trichlorosilane in a microwave reactor at 150 °C, to afford the P-reduced products after 20 minutes. This non-stereoselective reaction^52^ led to isomeric mixtures of 1 (*δ*^31^P: −27.2 ppm, −27.5 ppm) and 2 (*δ*^31^P: −26.6 ppm, −26.8 ppm), even when using pure diastereomers as precursors. The trivalent species are prone to oxidation under ambient conditions, precluding their separation. Consequently, compounds 1 and 2 were purified under inert atmosphere as isomeric mixtures.

The reaction of 1 and 2 with an excess amount of methyl triflate in CH_2_Cl_2_ (0 °C → RT) led to the dicationic phosphorus derivatives 1-Me (*δ*^31^P: 0.1 ppm, −0.4 ppm) and 2-Me (*δ*^31^P: −1.4 ppm, −1.9 ppm), respectively. The charged 1-Me and 2-Me are stable under ambient conditions but inseparable by chromatographic techniques due to their high polarities. Hence, purification and characterization were performed on the mixture of isomers, similar to the trivalent congeners.

**Fig. 2:**
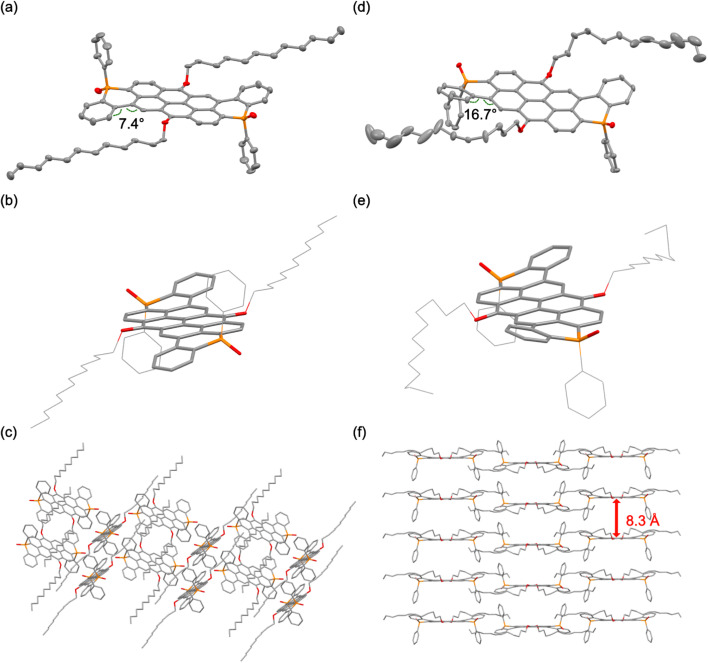
Solid-state structures obtained by crystallization from toluene of *trans*-1-O: (a) single molecule, (b) molecular curvature, (c) packing; and *cis*-1-O (d) single molecule (one enantiomer shown), (e) molecular curvature, (f) packing ((a and d) 50% probability level, H-atoms omitted for clarity).

The structures of *trans*-1-O and *cis*-1-O in the solid state, depicted in Fig. 2, S1 and S2,‡ were determined by single crystal X-ray diffraction. Notably, *cis*-1-O is also chiral and crystallizes as racemic mixture (Fig. S2a‡). The noticeably curved π-system of the extended core in both *trans*-1-O and *cis*-1-O results from steric hindrance of the H atoms in the bay positions, selectively twisting the terminal rings away from the P-phenyl groups and leading to overall S- and U-shaped molecular scaffolds for *trans*-1-O and *cis*-1-O, respectively (torsion angles *trans*-1-O: 7.4°; *cis*-1-O: 16.7° and 16.3°/11.8° for the two enantiomers; Fig. 2, S1 and S2‡). Moreover, the orientation of the P-centers also has a pronounced influence on the supramolecular packing. In sharp contrast with the parent VO3, the P-extended species surprisingly exhibit little to no π-stacking in either case, despite their nanocarbon scaffolds. Compound *trans*-1-O exhibits a slipped stack, herringbone-type arrangement with dodecyl chains separating the neighboring π-systems in an interdigitated fashion (Fig. 2c). Conversely, the molecular packing of the racemic mixture of *cis*-1-O exhibits an “H-type” stacking of the conjugated systems, despite the long distance of 8.3 Å with the voids again filled by parts of the dodecyl chains (Fig. 2f and S2‡). We were also able to crystallize two additional polymorphs of *trans*-1-O with solvated water and chloroform, respectively (Fig. S1b and c‡). Despite similar molecular conformations overall, each polymorph of *trans*-1-O shows a distinct supramolecular organization due to the co-crystallized solvates. This also results in slightly altered torsion angles of 15.9° and 12.3° (Fig. S1‡).

The solid-state structure of the thieno-congener *trans*-2-O (Fig. S3‡) shows a herringbone-type packing pattern and a similarly curved molecular structure as *trans*-1-O, with a torsion angle of 9.6° (Fig. S3‡). These results underscore the strong impact of the stereochemistry of the phosphorus centers that reflects earlier results on smaller linear 1D heteroacenes,^53^ but also the steric footprint and torsional flexibility of the terminal fused rings on the supramolecular organization in the solid state. Notably, the system's proclivity for aggregation in different environments is quite evident and suggests an inherently broader scope for fine-tuning the supramolecular organization of these large 2D nanocarbon systems compared to the significantly smaller relatives reported earlier.^6^

**Fig. 3:**
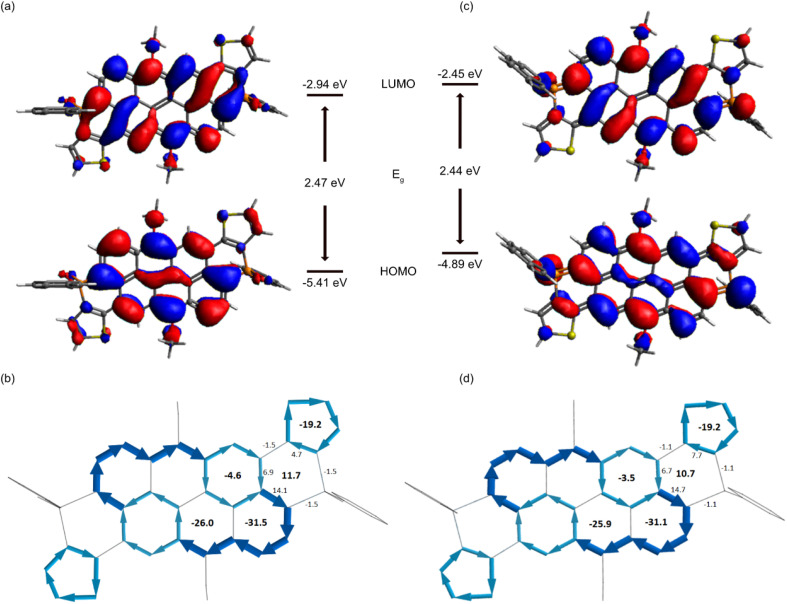
FMOs of *trans*-2-O′ (a) and *trans*-2′ (c); NICS values and ring current of *trans*-2-O′ (b) and *trans*-2′ (d); note: bond current vectors for the P-heterocycles are too small to be visible; corresponding values are included as numbers on the bonds instead.

To gain some deeper insight into the effect of phosphacycle extension of the VO3 core and the delocalized π-system of the new nanocarbons, we performed DFT calculations (see ESI‡ for technical details). The frontier molecular orbitals (FMOs) are shown in Fig. 3 and S4.‡ Illustratively, the FMOs of *trans*-2-O′ and *trans*-2′ (dodecyl truncated to Me) are spread over most of the scaffold including the phosphorus centers. The energy of the highest occupied molecular orbital (HOMO) is −5.41 and −4.89 eV for *trans*-2-O′ and *trans*-2′, respectively, while the energy of the highest unoccupied molecular orbital (LUMO) is −2.94 and −2.45 eV for *trans*-2-O′ and *trans*-2′, respectively (Table 1). Reducing the phosphorus centers to their trivalent state thus leads to an increase of the FMO energies, but with a stronger impact on the HOMO, reducing the energy gap overall. By contrast, methylating the P-center significantly lowers the FMO energy levels to around −4.12 and −6.18 eV for HOMO and LUMO, respectively (Table 1). Nucleus Independent Chemical Shift (NICS) analysis confirms that the anthanthrene core is well preserved in all the synthesized species (Fig. 3).^54^ The ring current is symmetrically distributed over the extended core. Notably, the formally antiaromatic phosphacycles separate the aromatic currents from the anthanthrene core and the terminal benzo or thienyl rings, respectively.

**Table 1:** Photophysical, electrochemical, DFT data of the 1 and 2 series of compounds

Compd	*λ*_abs_ a/nm	*ε* a/M^−1^ cm^−1^	*λ*_em_ a/nm, *Φ*_f_^b^	*λ*_em_/nm 0.2, 1.2%^c^	*Φ*_f_ 0.2, 1.2%^d^	*E*_red1,2_ e/V	*E*_LUMO_/eV (CV)^f^	*E*_LUMO_, *E*_HOMO_/eV^g^	*E*_g_/eV^h^
1-O_*t*_	356, 509	94 300	528, 0.55	560, 617	0.45, 0.24	−1.70, −2.04	−3.10	−2.85, −5.37	2.52
1-O_*c*_	356, 509	58 600	526, 0.37	559, 632	0.35, 0.19	−1.64, −1.99	−3.16	−2.85, −5.38	2.53
1	369, 510	134 200	559, 0.39	562, 587	0.15, 0.08	−1.99	−2.81	−2.42, −4.91 (*t*)	2.49
								−2.42, −4.91 (*c*)	2.49
1-Me	363, 533	88 400	573, 0.41	578, 632	0.45, 0.11	−1.24, −1.59	−3.56	−4.08, −6.18 (*t*)	2.10
								−3.61, −6.09 (*c*)	2.48
2-O_*t*_	366, 517	40 500	536, 0.52	575, 657	0.25, 0.13	−1.58, −1.91	−3.22	−2.94, −5.41	2.47
2-O_*c*_	367, 517	61 700	537, 0.22	573, 683	0.13, 0.06	−1.59, −1.96	−3.21	−2.94, −5.40	2.46
2	376, 514	67 200	576, 0.31	586, 626	0.09, 0.06	—	—	−2.45, −4.89 (*t*)	2.44
								−2.46, −4.90 (*c*)	2.44
2-Me	373, 540	57 500	585, 0.35	590, 656	0.27, 0.10	−1.13, −1.47	−3.67	−4.12, −6.18 (*t*)	2.06
								−4.12, −6.19 (*c*)	2.07

a Absorption/emission maxima and molar extinction coefficient obtained from CH_2_Cl_2_ solutions.

b Fluorescence quantum yield determined *via* integrating sphere.

c Emission maxima from PMMA thin films containing 0.2 and 1.2% P-modified vat dyes.

d Fluorescence quantum yields of PMMA thin films containing 0.2 and 1.2% of P-modified vat dyes determined *via* integrating sphere.

e Reduction potentials in volts obtained by cyclic voltammetry.

f LUMO energy in eV obtained from the CV measurements.

g Calculated HOMO and LUMO energies by DFT calculations.

h Calculated energy gap.

### Photophysical properties in solution

**Fig. 4:**
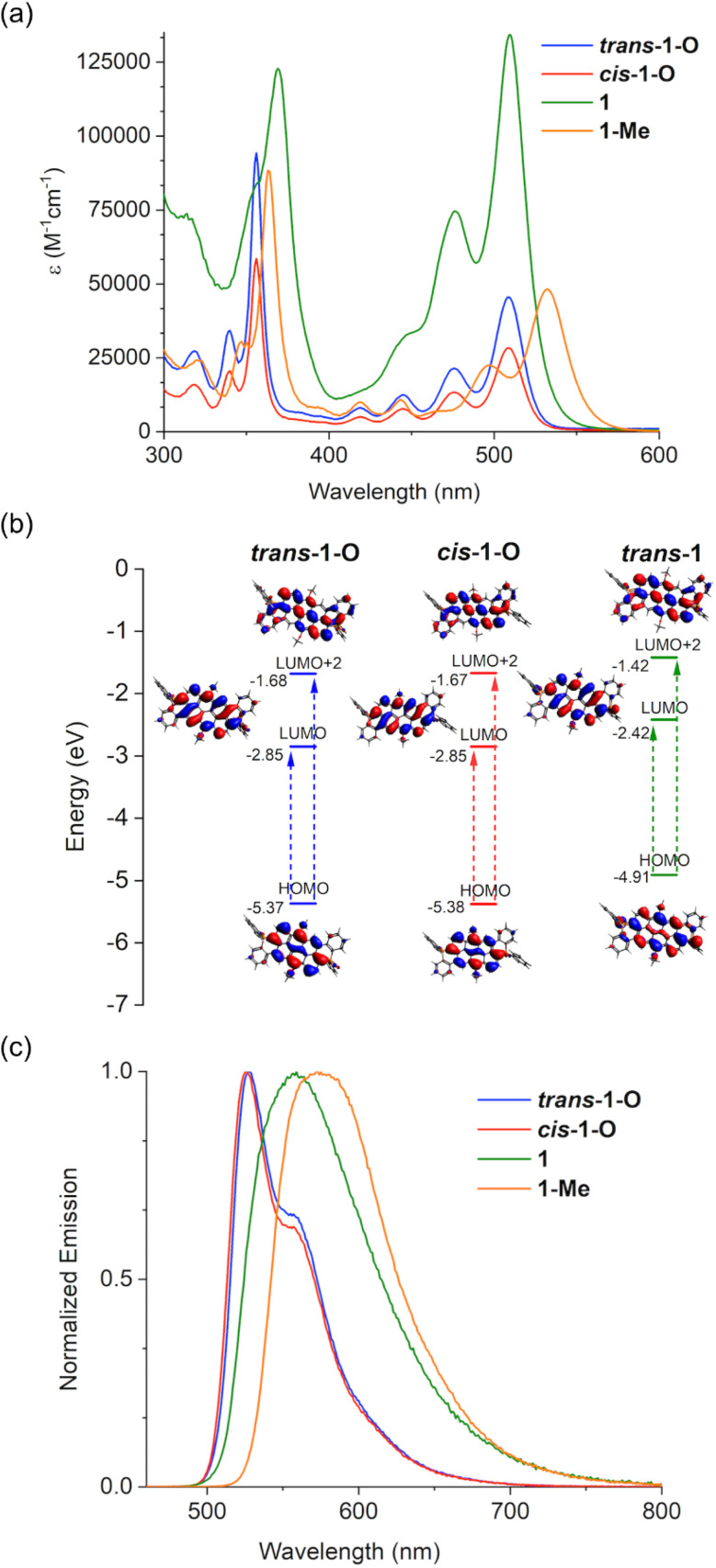
Absorption spectra (a), major transitions involved (b), and emission spectra (c) in CH_2_Cl_2_ of the benzo-fused isomers *trans*-1-O and *cis*-1-O along with the isomeric mixtures of P-reduced 1 and P-methylated 1-Me.

The photophysical characteristics of the new species are summarized in Table 1, and representative UV-vis absorption and emission spectra of the benzo-fused series 1 are shown in Fig. 4 (see Fig. S9‡ for spectra of the thieno-fused series 2). Their overall photophysics are more complex, have considerably larger extinction coefficients, and are tunable over a wider range than those of previously reported smaller P-heteroacenes,^6,53^ but largely resemble those of related functionalized anthanthrenes.^26,55^ The observed superior photophysics can thus be attributed to the distinct molecular geometry of the extended 2D nanocarbon scaffold.

Specifically, all absorption spectra of the 1 and 2 series of compounds exhibit high- (356–376 nm) and low-energy (509–540 nm) absorption peaks, each with additional vibrational sidebands. Representative TD-DFT calculations for *cis*-2-O indicate that the low-energy absorption arises from HOMO → LUMO transition, and the high-energy absorption consists of a combination of HOMO → LUMO+2, HOMO-3 → LUMO, and HOMO-1 → LUMO+1 transitions (see Fig. S7‡). These are symmetry-allowed *a*_g_-to-*b*_u_ or *b*_u_-to-*a*_g_ excitations. Here, we use the irreducible representations of the *C*_2h_ symmetry of the core planar structure to label the orbitals. The latter is a typical feature for rigid polyaromatic hydrocarbons including anthanthrenes.^26^

The overall absorption patterns for the isomers *trans*-1-O and *cis*-1-O as well as their *λ*_max_ values are quite similar (356 and 509 nm; Fig. 4 and Table 1). The same is true for *trans*-2-O and *cis*-2-O, albeit somewhat shifted (366 and 517 nm; Fig. S9a‡), because of the altered electronics resulting from different terminal rings in 1 and 2 series. However, the extinction coefficients of *trans*-1-O are larger than those of *cis*-1-O, while the situation is reversed for *trans*-2-O and *cis*-2-O (Table 1). While TD-DFT calculations show qualitatively identical oscillator strengths within each pair of *cis* and *trans* isomers (Table S1‡), we nonetheless posit that the observed extinction coefficients are due to different absorption cross-sections from geometrically distinct isomers.^6^ The photophysics of the trivalent species 1 and 2 experience a small redshift for the high energy bands with Δ*λ*_abs_ = 13 nm for 1 and Δ*λ*_abs_ = 10 nm for 2 (Fig. 4 and S9‡), and their extinction coefficients are the highest in each of their respective series (up to 134 200 M^−1^ cm^−1^). Methylation of the P-centers commonly stabilizes the LUMO in similar systems,^56,57^ and this is also manifested in the low energy bands for 1-Me and 2-Me that have the most red-shifted Δ*λ*_abs_, with notable values of 24 and 23 nm, in their respective series.

In general, replacing the terminal benzo unit with a thieno ring (*i.e.*, 1 → 2) red-shifts the absorption bands, which is also confirmed by DFT calculations (see ESI‡). Moreover, the extinction coefficients are high for all species (40 500–134 200 M^−1^ cm^−1^), making them attractive materials for a range of applications (*e.g.*, sensors, organic solar cells).

The emission spectra of *trans*-1-O and *cis*-1-O exhibit a similar shape and emission maxima at *λ*_em_ = 527 nm, respectively (Fig. 4b). This is also the case for *trans*-2-O and *cis*-2-O (Fig. S9b‡), overall exhibiting different shades of green fluorescence. Yet the quantum yields for *trans* isomers are notably much higher than those of the *cis* congeners in both systems, which can also be ascribed to distinct molecular geometries and flexibility of the twisted scaffolds in the excited state (Table 1). Reduction of the P-centers significantly red-shifts the emission to yellow (Δ*λ*_em_ = 32 nm for 1; 40 nm for 2) (Fig. 4a and S9b‡), an effect also found in other conjugated compounds with six-membered phosphorus heterocycles.^56,57^ Methylation causes a further redshift compared to the oxidized species toward orange luminescence (Δ*λ*_em_ = 46 nm for 1-Me; 49 nm for 2-Me (Fig. 4b and S9b‡). This confirms the considerable impact of the P-center on the overall electronics and photophysics of the scaffold. The quantum yields of all the new species range from moderate to high (22–55%) and with the benzo-fused series having slightly higher values than the thieno congeners (Table 1). The excited-state lifetimes of *trans*-1-O and *cis*-1-O species range between *τ* = 3–4 ns, clearly categorizing the emission as fluorescence (Fig. S12a‡).

**Fig. 5:**
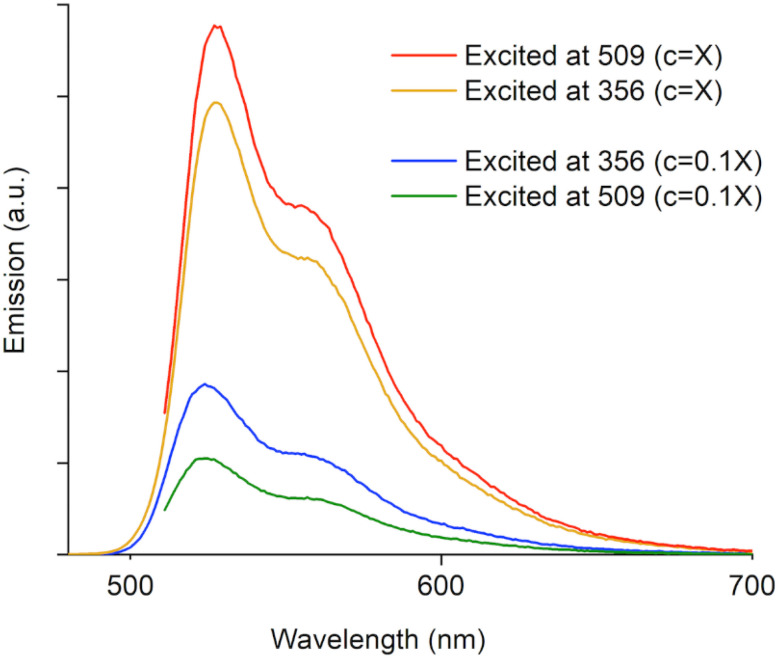
Concentration-dependant emission spectra of *trans*-1-O excited at 356 and 509 nm in CH_2_Cl_2_ (*X* = 6.4 × 10^−6^ M).

The two distinctive sets of absorption bands in all the species intrigued us to probe the excited-state photophysics and potential light-harvesting features of the system (Fig. 5).^58,59^ As representative example, the emission spectra of *trans*-1-O excited at 356 nm and 509 nm revealed that although the emission maxima and overall patterns are alike, the relative intensities of the emissions depend on the excitation wavelength and also the concentration of the solution. At low concentration (*i.e.*, *c* = 10^−7^ M), the spectrum excited at 356 nm has a higher emission intensity than the one excited at 509 nm. As the excitation at 356 nm creates more excitons in higher excited states (*vide supra*), it is only logical to assume that at least some return to the LUMO *via* internal conversion (Kasha's rule), leading to a higher emission intensity than the spectrum excited at 509 nm, where the excited state exclusively involves the LUMO.^60^ However, at higher concentrations (*c* > 6 × 10^−6^ M) the emission spectrum excited at 365 nm is less intense; this can be attributed to exciton quenching from non-radiative processes and molecular collisions due to aggregation. This is also reflected by the relative peak intensities in the respective excitation spectra (Fig. S11‡), and similar results were obtained for all the new π-conjugated compounds of this series. These results reveal that controlling the aggregation for these systems in solution state is practically achievable.

### Emission properties in the solid state

**Fig. 6:**
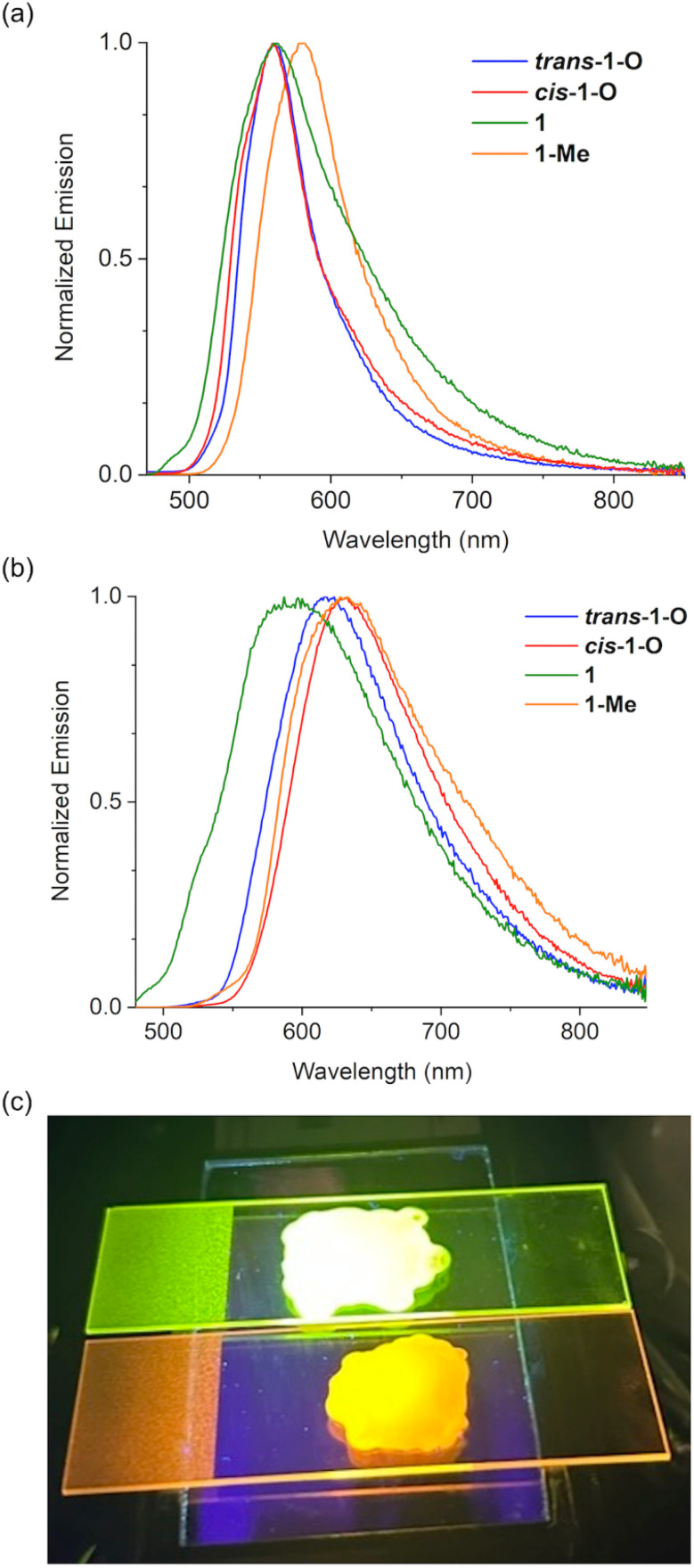
Emission spectra of the PMMA films containing (a) 0.2 wt% and (b) 1.2 wt% of the benzo-fused series 1. (c) Emission color of the PMMA films with 0.2 wt% (top), and 1.2 wt% (bottom) *trans*-1-O.

To date, solid-state emission data have not been reported for any of the related systems with 6-membered phosphacycles in the literature.^6,53^ We were thus interested in uncovering the emissive behavior of the new species in the solid state, as it could provide tangible insight into the potential utility of the materials in practical applications. Not surprisingly, all species were found to display negligible emission in the bulk, as emission quenching is already observed in solution (*vide supra*). This is indicative of strong intermolecular interactions that lead to non-radiative relaxation processes for these expansive π-systems. We thus surmised that separating individual molecules in a rigid matrix could lead to effective emission in solid state. Indeed, when polymethyl methacrylate (PMMA) is used as a matrix to seclude the nanocarbon species, pronounced luminescence emerges for all species. Their emissive behavior was assessed by preparing 0.2 and 1.2 wt% films of the respective species in PMMA (Fig. 6). Intriguingly, the emissions of the 0.2 wt% films for all the compounds in series 1 and 2 are very intense with fluorescence quantum yields up to 45% (Table 1). In particular, the 0.2 wt% films of the benzo-fused *trans*-1-O and *cis*-1-O display quite similar *λ*_em_ values, whereas a redshift is observed for 1 and 1-Me, akin to the trends seen in solution (Fig. 6a and 4b). More importantly, slightly increasing the concentration to 1.2 wt% has a surprisingly strong effect. The *λ*_em_ values are considerably red-shifted, which is reflected in a switch of the emission colors of the films, *i.e.*, from green to orange for *trans*-1-O (Fig. 6c). In fact, the emission wavelength differences (Δ*λ*_em_) between the PMMA films with 0.2 and 1.2 wt% of the benzo-fused series range from 25 nm for 1 up to 73 nm for *cis*-1-O. In particular, when comparing the emission spectra of the 0.2 and 1.2 wt% films, broader, red-shifted profiles are observed for the films with higher concentration in all cases, suggesting the formation of J-type excimers or aggregates of the dye-based compounds (Fig. 6a and b).^61–63^ Intriguingly, the excited-state lifetimes of the 0.2 wt% films of *trans*-1-O and *trans*-2-O exhibit a 3–4 ns component that is close to the values in solution, but also another component with a longer lifetime of around 10–13 ns that suggests the presence of aggregates already at low concentration. Moreover, the 1.2 wt% films display fluorescence lifetimes of around 30 ns which supports strong aggregation, but a component with a lifetime of about 4–6 ns is still present here as well (Fig. S12b and c‡). Thus, even though aggregates may be responsible for the red-shifted emissions, in the PMMA matrix, the latter appear to be sufficiently isolated from additional deactivation pathways that could lead to a rapid dissipation of the excited state energy.

Regardless of the specific underlying processes, such a drastic change in luminescence color and fluorescence lifetimes of a single, conjugated compound from only a minor change in the concentration is unique. In fact, it is also worth mentioning that films with more than 5 wt% of the phosphacycle-modified vat dyes exhibit aggregation-caused quenching of the emission, similar to the bulk solid.

The measurements on the thieno-fused series 2 provided similar emission patterns for the 0.2 wt% films (Table 1 and Fig. S10‡). However, Δ*λ*_em_ between the 0.2 and 1.2 wt% films for them is overall more pronounced (40 nm for 2 up to 110 nm for *cis*-2-O, Fig. S10‡). Moreover, the 1.2 wt% films of the thieno-fused series 2 possess considerable emission in the near-IR region. These results convincingly underpin that the delicate balance between molecular and intermolecular interactions of this system has a considerable impact on the overall solid-state photophysics.

**Fig. 7:**
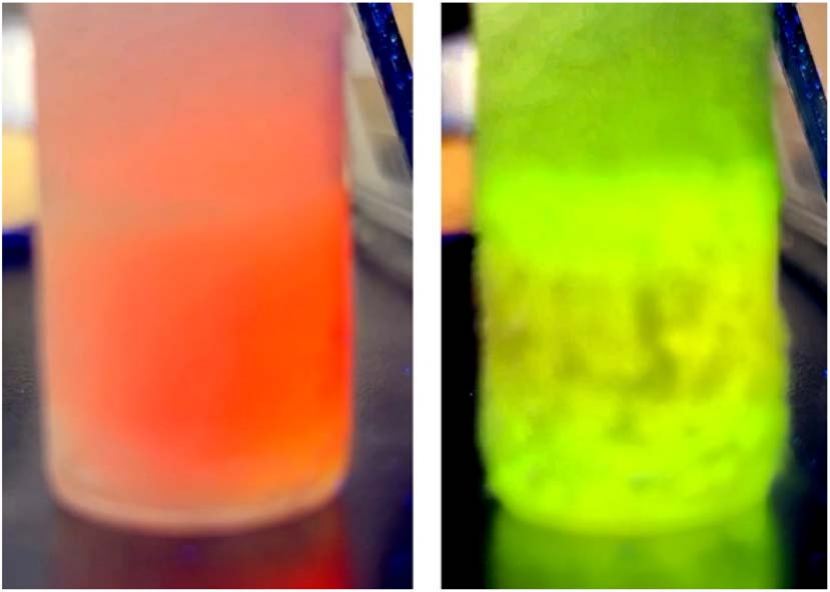
Fluorescence of the vials containing CH_2_Cl_2_ solutions of *trans*-1-O at 77 K (liquid N_2_) (left) and 2 minutes after being taken out of the liquid N_2_ (right).

To determine the influence of temperature on the photophysics of these systems and to inherently simulate the rigid environment of the thin films, a qualitative study was undertaken on *trans*-1-O. A CH_2_Cl_2_ solution of *trans*-1-O (*c* = 10^−6^ M) was cooled down to 77 K (liquid N_2_) before warming back up to room temperature, while the luminescence was monitored with an UV lamp at 365 nm (Fig. 7). Surprisingly, the orange emission of the frozen slurry correlated well with the emission color for the 1.2 wt% PMMA film. Upon slowly increasing the temperature, the color gradually changed to green, which is generally observed for solutions at room temperature. Additional experiments revealed that 157 K, sufficient to freeze CH_2_Cl_2_, is required to simulate the rigid environment of the PMMA films, *i.e.*, to trap the π-extended molecules close to each other and provide the aggregates with red-shifted emission. Notably, similar results were observed for both isomers of 1-O and 2-O, respectively.

### Further functionalization of the P-oxide species

**Scheme 3:**
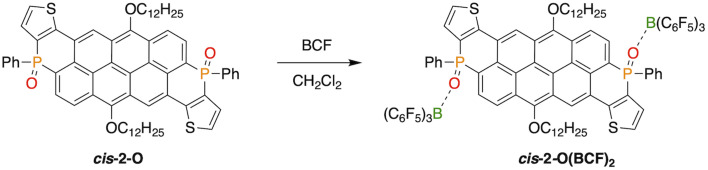
Generation of the Lewis adduct *cis*-2-O(BCF)_2_ in CH_2_Cl_2_ from *cis*-2-O.

Since the reduction toward the trivalent species in each series always led to an inseparable mixture of isomers, we were interested in maintaining of the isomeric purity upon P-modification. To this end, we investigated the coordination chemistry of *cis*-2-O with the strong Lewis acid B(C_6_F_5_)_3_ (BCF).^52^ We previously established that selective coordination of the P=O bond to a Lewis acid such as BCF red-shifts and often enhances the luminescence because of a stabilized LUMO.^64,65^ To our satisfaction, upon addition of BCF to a solution of *cis*-2-O, a prompt emission shift from green to orange was observed, indicating the formation of the adduct *cis*-2-O(BCF)_2_ (Scheme 3).

**Fig. 8:**
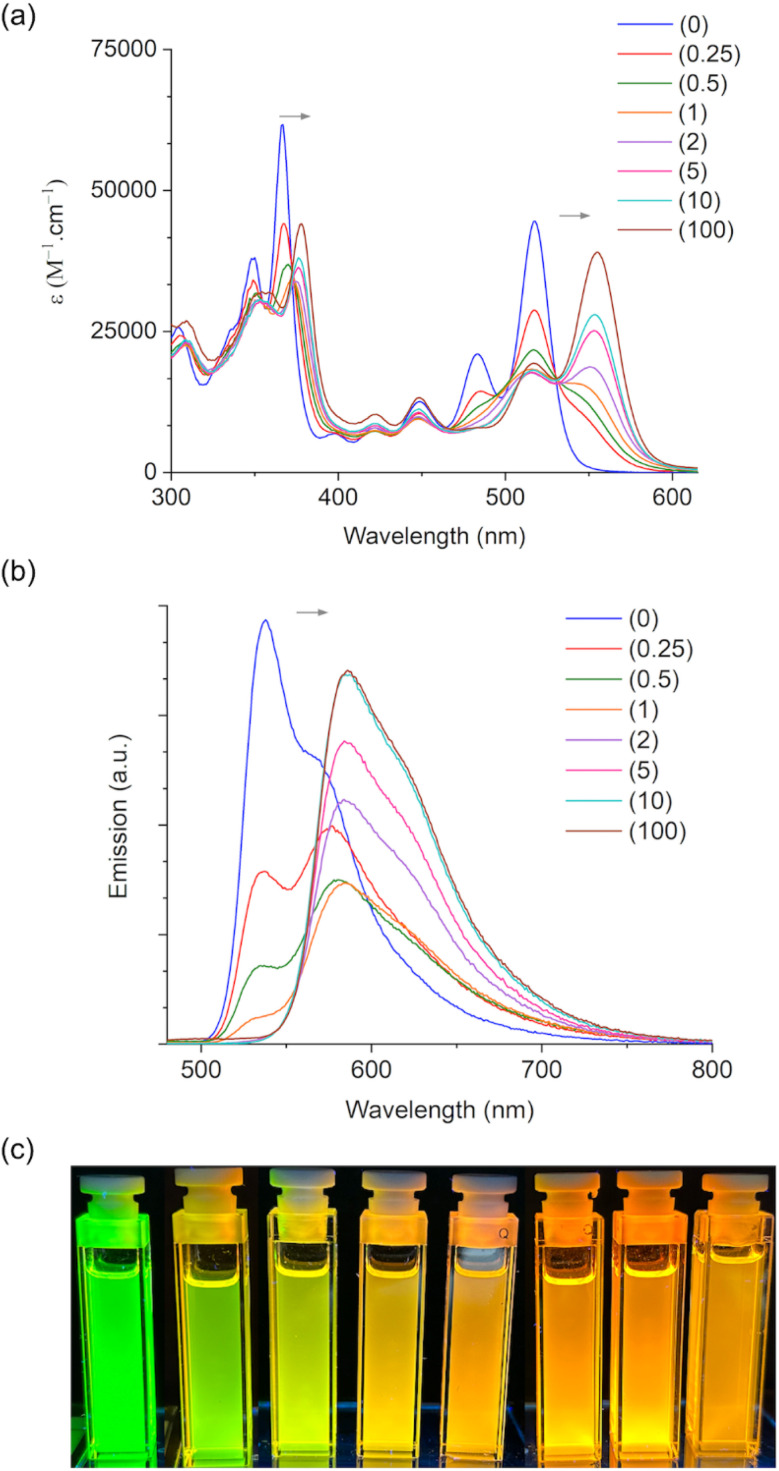
Absorption (a) and emission (b) spectra of *cis*-2-O upon titration with BCF in CH_2_Cl_2_. (c) Solutions with equal concentrations of *cis*-2-O with increasing amounts of BCF from 0 (left) to 100 (right) equivalents.

This was also supported by a pronounced low-field shift from *δ* = 7 to 19 ppm in the ^31^P{^1^H} NMR spectrum (See ESI‡). In order to track the coordination of BCF to the two phosphorus centers, *cis*-2-O was titrated with increasing amounts of BCF (0–100 equiv), and the resulting changes were measured by UV-vis spectroscopy (Fig. 8a). The clean conversion from *cis*-2-O to *cis*-2-O(BCF)_2_ was observed *via* two isosbestic points at 373 and 531 nm (Fig. 8a). While the high energy absorption peak is red-shifted by only 11 nm, the low energy band shifts by 38 nm. As revealed by the TD-DFT calculations (*vide supra*), the low-energy absorption arises from the HOMO → LUMO transition. This reveals that the newly added P-terminal rings to the π-system of the vat dyes have a stronger contribution to the lower-energy peaks upon functionalization.

The corresponding emission spectra were measured by exciting the solutions at the two isosbestic points (Fig. 8b and c). Although exact isoemissive points were not detected (likely due to both equilibria occurring at the same time), the presence of three species can be clearly distinguished in the emission spectra that are assigned as follows: uncoordinated *cis*-2-O, as well as coordinated *cis*-2-O(BCF), and *cis*-2-O(BCF)_2_, *i.e.*, with one or two BCF coordinated moieties, respectively.

At the outset, *cis*-2-O exhibits an emission maximum at *λ*_em_ = 537 nm, while in the presence of 0.25 equiv. of BCF, this peak diminishes and a new peak at 577 nm emerges, which is attributed to the monocoordinated *cis*-2-O(BCF). Upon increasing the amount of BCF beyond 1 equiv. (enough for only one P-center), the peak at 537 nm disappears, and similarly, a red-shifted peak at 585 nm emerges, suggesting the formation of *cis*-2-O(BCF)_2_ even with less than 2 equiv. of BCF. Excess of BCF then leads to the complete formation of *cis*-2-O(BCF)_2_ with a *λ*_em_ = 586 nm, with an overall redshift of 49 nm for the full conversion of *cis*-2-O to *cis*-2-O(BCF)_2_, which is more than twice the redshift observed for a related, but smaller 1D systems (20 nm) reported earlier.^52^ This again illustrates the strong influence of phosphorus modification on the overall electronic structure of the nanocarbon system. To further understand the spectral change induced by BCF, we compared the DFT-calculated results for *cis*-2-O and *cis*-2-O(BCF)_2_ (Fig. S7‡). BCF coordination lowers the LUMO energy more than the HOMO energy. Consequently, the low-energy absorption that arises from HOMO → LUMO excitation is noticeably red-shifted, which aligns with our previous work on dithienophospholes.^64^

The same coordination study was also performed for *trans*-1-O (Fig. S8‡), however, the best results were obtained for *cis*-2-O, which is attributed to the overall distinctly twisted scaffolds (*cis vs. trans*) and the resulting π-stacking interactions of one of the perfluorophenyl substituents of BCF with the extended nanocarbon main scaffold that is often observed for such systems.^53,64^

### Redox properties

**Fig. 9:**
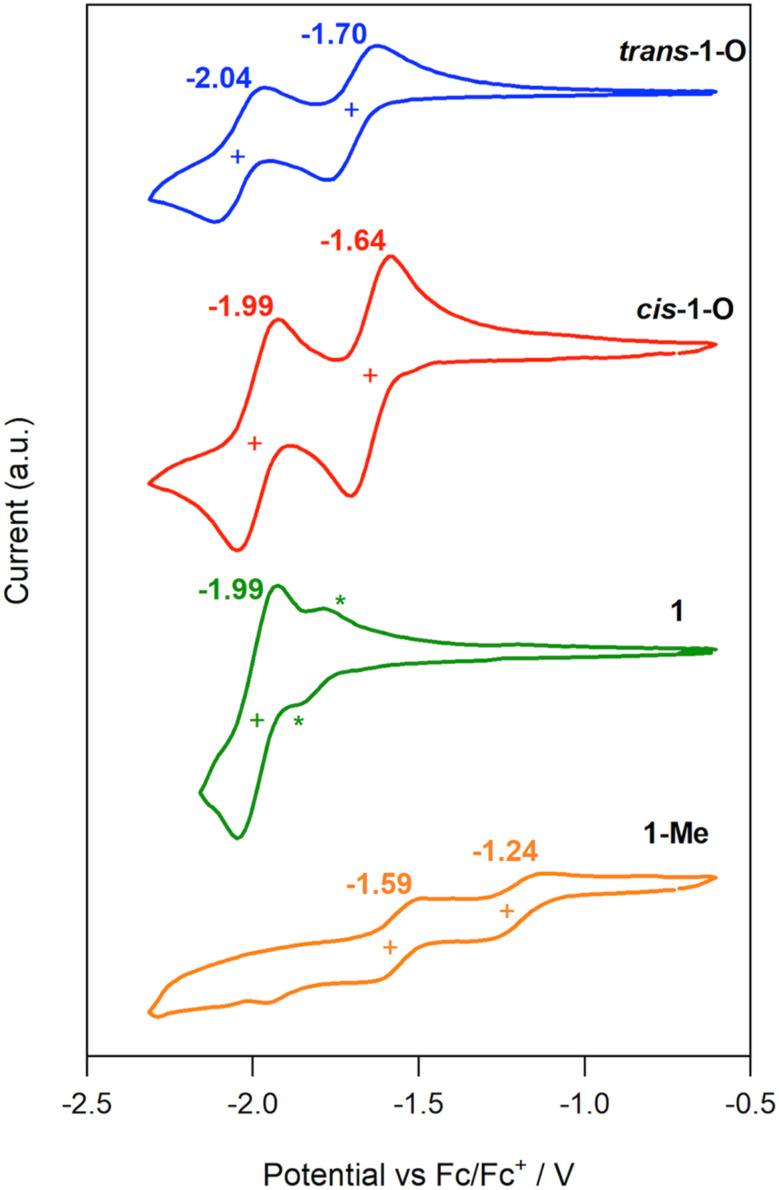
Cyclic voltammograms of *trans*-1-O (blue), *cis*-1-O (red), 1 (green; * indicate the anodic and cathodic redox processes for the second isomer), 1-Me (orange), with scan rate 100 mV s^−1^, in CH_2_Cl_2_ with 0.1 M NBu_4_PF_6_, *vs.* Fc/Fc^+^.

To measure the influence of the electron-withdrawing phosphorus-containing moieties on the electron-rich anthanthrene core of VO3, cyclic voltammetry was performed on all new species (see Fig. 9 for series 1 and S13 for all species). To our satisfaction, *cis*-1-O and *trans*-2-O each exhibit two reversible reduction peaks with *E*_red1_ at −1.64 and −1.58 V, respectively. While *trans*-1-O and *cis*-2-O have fully reversible (or quasi-reversible) first reduction peaks (−1.70 and −1.59 V), their second reductions are quasi-reversible. The trivalent isomeric P-species 1 have reversible peaks at *E*_red1_ = −1.99 V and −1.81 V as the result of their higher-lying LUMO. It should be noted that this pronounced degree of (quasi) reversibility is not observed for the smaller P-containing, or native nanocarbon relatives in the literature that generally show irreversible reduction events.^6,53,66^ Methylating the P-centers causes a significant decrease in reduction potentials of 1-Me and 2-Me, lowering the *E*_red1_ to −1.24 and −1.13 V, albeit as quasi-reversible (or irreversible) reductions. Nonetheless, these values correlate well with low-lying LUMO and LUMO+1 energies of −3.56 and −3.67 eV, respectively, akin to those found in PCBM-C_60_ (−3.7 eV), a strong electron acceptor widely used in electronic devices.^67^ This is remarkable for a such an expansive π system. In addition, most compounds also show one or two oxidation peaks above −0.12 V but all are irreversible,^68^ which is typical for phosphorus-containing conjugated materials.

## Conclusion

We successfully obtained a new family of π-conjugated 2D-nanocarbons through innovative derivatization of Vat Orange 3 with two phosphorus heterocycles. Our approach provides access to significantly value-added vat dyes, *i.e.*, materials with a longstanding history and multi-ton annual production, thus paving the way for scalable preparation. The new benzo- and thieno-fused species with two chiral, and easily modifiable phosphorus centers present as two diastereomers for each species with distinct packing in solid state, while maintaining similar electronic properties. These materials exhibit not only rich and straightforward chemistry but also intriguing emissive properties in both solution and solid state. They are strong UV-vis absorbers with extinction coefficients of up to 134 200 M^−1^ cm^−1^ and identical *λ*_max_ values for the *trans* and *cis* isomers, respectively. Moreover, their photophysical properties can be tailored by P-functionalization, resulting in redshifts in both absorption and emission spectra. Indeed, both the benzo- and thieno-fused vat derivatives can be endowed with intriguing emission properties with moderate to high fluorescence quantum yields. PMMA films of the P-functionalized vat dyes revealed interesting solid-state photophysics. At a very low concentration of fluorophore (0.2 wt%), the films exhibit strong emission with a slight red shift compared to their solutions. Surprisingly, a minimal increase in concentration of only 1 wt% gives rise to a dramatically red-shifted emission, which we attribute to the formation of aggregates, together with a remarkable increase in fluorescence lifetime. A similar effect was observed for a frozen slurry at 77 K; the solid matrix displayed red-shifted emission, while thawing recovered the original green-yellow emission of the species in solution. Coordination of BCF to the P=O groups of individual diastereomers results in a clean conversion to Lewis adducts with strongly red-shifted photophysics (from green to orange), while maintaining the isomeric purity of the species. Cyclic voltammetry revealed the existence of reversible reduction peaks for 1-O and 2-O, along with very low reduction peaks and LUMO levels at *ca.* −3.6 eV for 1-Me and 2-Me, rivaling those of widely used fullerene acceptors. Therefore, the integration of phosphorus heterocycles into 2D nanocarbons not only inherently enhances the properties of purely carbon-based systems, but also endows the system with an intriguing degree of tunability that is highly desirable for functional materials chemistry.

All-in-all, this study provides a fundamental cornerstone that underscores the significant potential of main-group chemistry in elevating conjugated materials, particularly by establishing the chemistry of phosphorus-embedded nanocarbons. The first successful integration of the advantageous functional properties of organophosphorus species and the economical availability and abundance of Vat Orange 3, allowed us to synthesize a highly value-added family of materials. Based on the promising results from this initial study, we are currently broadening the scope of organophosphorus-based vat dyes by exploring further molecular scaffolds and architectures.

## Data availability

All the data can be found within the manuscript and ESI files.‡

## Author contributions

The manuscript was written through contributions of all authors. All authors have given approval to the final version of the manuscript. Author contributions are as follows: R. D.: formal analysis, investigation, methodology, visualization, writing – original draft; J. L.: formal analysis, investigation; E. P.: formal analysis, investigation; D. M.: investigation, resources; A. H.: investigation, resources, H. N. H.: formal analysis, investigation; T. Z.: formal analysis, funding acquisition, validation, supervision, writing – original draft; C. R. N: conceptualization, funding acquisition, validation, project administration, writing – review and editing; T. B.: conceptualization, funding acquisition, validation, project administration, supervision, writing – review and editing.

## Conflicts of interest

There are no conflicts to declare.

## Supplementary Material

SC-016-D4SC07106A-s001

SC-016-D4SC07106A-s002
