# Supplementary material for: From flat to twisted – multifunctional phosphacyclic nanocarbons based on Vat Orange 3
Source: Chem Sci. 2025 Jan 28;16(8):3680–92. doi: 10.1039/d4sc07106a (PMC11774316; doi:10.1039/d4sc07106a)
Supplement: SC-016-D4SC07106A-s001 [file SC-016-D4SC07106A-s001.pdf]

Supplementary Information (SI) for Chemical Science.
This journal is © The Royal Society of Chemistry 2025

From Flat to Twisted – Multifunctional Phosphacyclic Nanocarbons Based on Vat Orange 3

Reza Dadgaryeganeh,[a] Jesse LeBlanc,[a] Ekadashi Pradhan,[a] Dandan Miao,[a] Amaar Hussein,[a] Howard N. Hunter,[a] Tao Zeng,[a,] Carlos Romero Nieto,[b,]* Thomas Baumgartner[a,]**

[a] *Department of Chemistry, York University, 4700 Keele St, Toronto, ON M3J 1P3, Canada*

[b] *Facultad de Farmacia, Universidad de Castilla-La Mancha, Calle Almansa 14, Edificio Bio-Incubadora, 02008, Albacete, Spain.*

email: tzeng@yorku.ca; carlos.romero-nieto@uclm.es; tbaumgar@yorku.ca

Table of Contents:

1. General section **......** ***2***

2. Experimental details: **......** ***4***

2.1 Synthesis 4

3. X-ray diffraction data **......** ***18***

4. Theoretical calculations **......** ***21***

4.1. Computational Details 21

4.2. Summary of Calculated Results 22

4.3. NICS Results and Aromaticities of the series 1 and 2 23

4.4. Results of TD-DFT Calculations and the Simulated Absorption Spectra 24

4.5. Frontier Molecular Orbitals 25

4.1. Computational Investigation of the Interaction of *cis*-2-O with BCF 29

5. Steady-state spectroscopy **......** ***32***

6. Electrochemical characterization **......** ***35***

7. NMR Spectra **......** ***37***

8. Coordinates (in Å) of optimized geometries of all 12 vat compounds and cis-2-O(BCF)$_2$ **......** ***75***

9. References **......** ***102***

1. General section

Reactions were carried out in dry glassware and under an inert atmosphere of purified argon using Schlenk techniques. Anhydrous solvents such as CH_2Cl_2, THF, pentane, were used directly from a solvent purification system (MBraun) or, in case of reactions or column chromatography, were purchased from commercial suppliers and used as received. Standard solvents such as MeCN, chloroform ($CHCl_3$), CH_2Cl_2, ethanol (EtOH), *n*-hexane, methanol (MeOH), and toluene were purchased from commercial suppliers and used as received. Deuterated solvents such as chloroform-d were purchased from commercial suppliers and used as received. Aluminum oxide, aq. ammonia solution (NH_4OH) (aqueous solution with 25% - 30% NH_3 basis), Celite®, 2,3-dibromothiophene, hydrochloric acid (HCl) (36.5 - 38% aq. solution), magnesium sulfate ($MgSO_4$), *n*-butyllithium (^{n}BuLi) (2.5 M solution in hexanes), potassium carbonate (K_2CO_3), potassium phosphate tribasic (K_3PO_4), silica gel, sodium carbonate (Na_2CO_3), sodium hydrogen carbonate ($NaHCO_3$), sodium hydroxide (NaOH), tetrakis(triphenylphosphine)palladium(0) ($Pd(PPh_3)_4$), 2-thienylboronic acid, trichlorosilane ($HSiCl_3$), triethylamine (Et_3N), [1,1′-Bis(diphenylphosphino)-ferrocene]dichloropalladium(II)($Pd_2(dppf)Cl_2$ were purchased from commercial suppliers and used as received.

NMR: ^{1}H, ^{13}C, and ^{31}P NMR as well as COSY, HSQC and HMBC spectra were recorded on a Bruker NEO 400, 700 spectrometer (400 MHz (^{1}H), 101 MHz (^{13}C), 162 MHz (^{31}P); 700 MHz (^{1}H), 176 MHz (^{13}C), respectively). The chemical shift δ values were corrected to $CDCl_3$ (δ 7.26 ppm for ^{1}H, δ 77.16 ppm for ^{13}C). Signal descriptions include: s = singlet, d = doublet, t = triplet, q = quartet, p = pentet, m = multiplet and br = broad. All coupling constants are absolute values and *J* values are expressed in Hertz (Hz). All ^{13}C signals are singlets unless stated otherwise.

X-Ray crystallography: X-ray crystal structure analyses were measured at 173 K on a Bruker DQ Quest eco instrument. The structure was solved and refined using OLEX2 and SHELXL. CCDC numbers 2370339 (***trans*-1-O** without solvent), 2370340 (with H_2O), 2370341 (***trans*-1-O**, with $CHCl_3$), 2370342 (***cis*-1-O**), 2370338 (***trans*-2-O**) contain the supplementary crystallographic data for this paper. These data can be obtained free of charge from The Cambridge Crystallographic Data Centre via www.ccdc.cam.ac.uk/data_request/cif.

Microwave reactions: Microwave reactions were carried out with an Anton Paar Monowave 200 microwave reactor using suitable reaction vials with Teflon-coated silicone caps.

Theoretical calculations: Molecular optimizations were carried out at the B3LYP/6-311+G(d) level by using the ORCA suite of programs.[S7] Solvent effects (CH_2Cl_2) were considered by using the polarization continuum model (PCM).

Steady-state spectroscopy: Absorption and emission spectra were recorded from CH_2Cl_2 solutions using an Agilent Technologies Cary 5000 UV-Vis spectrometer and Edinburgh FS5 instrument spectrometer with a SC-05 standard cuvette holder, respectively.

Fluorescence quantum yields *Φ*: Quantum yields were determined using integrating sphere.

Fluorescence lifetimes τ: The fluorescence decays were recorded with an Edinburgh FS5 instrument spectrometer using TCSPC. Fluorescence lifetimes were acquired by an exponential fit according to the least mean square with Fluoracle software.

Electrochemistry: Cyclic voltammograms were recorded using a Metrohm Autolab potentiostat from CH_2Cl_2 solutions using tetrabutylammonium hexafluorophosphate as electrolyte, glassy carbon as working electrode, platinum wire as counter electrode and silver/silver chloride (Ag/Ag^+) as pseudo-reference electrode. Scan rate is 100 mV s^{-1}. The curves were calibrated using ferrocene as internal standard ($E_{1/2}$ = 0.60 V). The voltammogram was recorded using the NOVA software package.

Preparation of the films: Polymethyl methacrylate (PMMA; 0.5 g) was added to 10 m CH_2Cl_2 solution in a 20 ml vial and stirred overnight. Then, 4 ml of the aforementioned solution (containing 0.2 g PMMA) was added to another 20 ml vial and subsequently charged with 2.4 mg of the compound from series **1** or **2**, and stirred for 1 h. The resulting solution was drop-cast onto a glass slide and the solvent evaporated subsequently. The prepared 1.2 wt% PMMA films were placed in an oven at 80 °C for 30 minutes to fully dry and then directly used in the fluorimeter. For preparing 0.2 wt % films, 0.4 mg of the respective conjugated compound and 4 ml of CH_2Cl_2 solution of PMMA (containing 0.2 g PMMA) were used in the identical procedure.

A blank slide was used as a reference to establish the absence of additional emission features arising from the glass that could interfere with the film measurements.

2. Experimental details

2.1 Synthesis

Compound S1

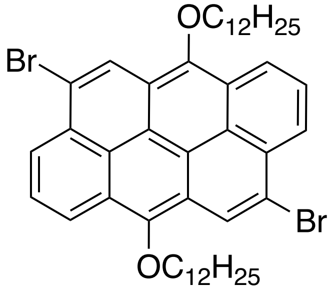

Based on a modified procedure.[S1] To a 250 ml Schlenk flask under argon, vat orange 3 (1.0 g, 2.1 mmol), dodecyl bromide (4.3 g, 17.2 mmol), aliquot 336 (1.2 ml, 2.6 mmol), Na2S2O4 (0.97 g, 5.6 mmol) were added, then dissolved in 100 ml of NaOH solution (0.1M) and the mixture was bubbled with argon for 10 minutes. The reaction was stirred at 70 °C for 3 h. After cooling to room temperature, the water was decanted, and the yellowish-orange solid was filtered and washed with methanol and then acetone. **S1** was purified by flash column chromatography using silica (CH_2Cl_2-Hex, 1:1), (85% yield, 1.47g).

^{1}H NMR (700 MHz, $CDCl_3$) δ 8.79 (d, *J* = 8.0 Hz, 2H), 8.77 (s, 2H), 8.63 (d, *J* = 7.4 Hz, 2H), 8.21 (t, *J* = 7.7 Hz, 2H), 4.34 (t, *J* = 6.5 Hz, 4H), 2.16 (t, 4H), 1.77 (t, *J* = 7.6 Hz, 4H), 1.52 (p, *J* = 7.6 Hz, 4H), 1.45 (p, *J* = 7.0 Hz, 4H), 1.41 – 1.35 (m, 4H), 1.35 – 1.20 (m, 22H), 0.89 (t, *J* = 7.0 Hz, 6H). **^{13}C{^{1}H} NMR** (101 MHz, $CDCl_3$) δ 149.0, 130.4, 126.4, 126.1, 125.6, 125.0, 124.7, 123.5, 122.1, 121.9, 119.2, 32.0, 30.8, 29.8, 29.7, 29.4, 26.3, 22.7, 14.2.

Compound S2

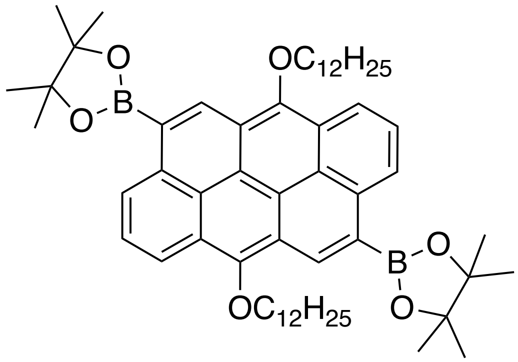

Based on a modified procedure.[S2] **S1** (2.1 g, 2.6 mmol), B_2pin_2 (3.0 g, 11.8 mmol), Pd(dppf)Cl2 (155 mg, 0.19 mmol) and KOAc (1.5 g, 15.3 mmol) were added to a 250 mL Schlenk flask under argon. After adding 30 ml of dioxane, the mixture was deaerated by bubbling with argon for 10 minutes. The mixture was then refluxed at 100 °C under inert atmosphere for 20 h. Upon completion, 100 mL of methanol were added to the cooled mixture and the generated precipitate was filtered and washed with methanol and acetone. **S2** was purified by flash column chromatography using silica (CH_2Cl_2-Hex, 1:1) obtaining the product as a yellow solid, (62% yield, 1.62 g).

^{1}H NMR (400 MHz, $CDCl_3$) δ 9.16 (s, 2H), 9.10 (d, *J* = 7.5 Hz, 2H), 8.79 (d, *J* = 8.1 Hz, 2H), 8.18 (t, *J* = 7.9 Hz, 2H), 4.42 (t, *J* = 6.4 Hz, 4H), 2.96 – 2.88 (m, 2H), 2.23 – 2.12 (m, 5H), 1.88 – 1.76 (m, 8H), 1.50 – 1.38 (m, 12H), 1.38 – 1.24 (m, 55H), 0.93 – 0.86 (m, 12H). **^{13}C{^{1}H} NMR** (176 MHz, $CDCl_3$) δ 150.4, 134.1, 134.0, 126.1, 125.9, 125.6, 125.1, 121.2, 120.9, 120.4, 84.0, 32.1, 31.0, 29.9, 29.9, 29.9, 29.8, 29.5, 26.6, 25.2, 22.8, 14.3.

Compound S3

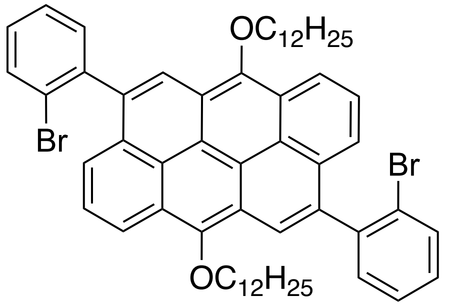

S2 (2.0 g, 2.2 mmol), 1,2-dibromobenzene (2.1 g, 8.9 mmol), $Pd(PPh_3)_4$ (254 mg, 0.22 mmol), K_2CO_3 (1.2 g, 8.9 mmol) were added to a 250 mL Schlenk flask under argon, then dissolved in dioxane/water (36/9 mL) and the mixture was deaerated by bubbling with argon for 10 minutes. The mixture was then refluxed at 100 °C for 20 h, cooled down to room temperature before 100 mL of water were added to the mixture. The crude product was filtered and washed with methanol and acetone. **S3** was purified by flash column chromatography using silica (CH_2Cl_2-Hex, 4:10), obtaining the pure product as a yellow powder (76 % yield, 1.59 g).

^{1}H NMR (400 MHz, $CDCl_3$) δ 8.83 (d, *J* = 8.2 Hz, 2H), 8.41 (s, 2H), 8.10 (t, *J* = 7.9 Hz, 2H), 7.88 (d, *J* = 8.0 Hz, 2H), 7.83 (d, *J* = 7.5 Hz, 2H), 7.69 – 7.60 (m, 2H), 7.56 (t, *J* = 7.4 Hz, 2H), 7.48 – 7.39 (m, 2H), 4.46 – 4.37 (m, 4H), 2.20 – 2.08 (m, 4H), 1.75 – 1.62 (m, 4H), 1.51 – 1.40 (m, 4H), 1.39-1.21 (m, 30H), 0.89 (t, *J* = 6.6 Hz, 6H). **^{13}C{^{1}H} NMR** (176 MHz, $CDCl_3$) δ 150.1, 141.8, 139.0, 133.2, 132.4, 131.1, 129.5, 127.6, 126.1, 126.0, 124.8, 124.6, 123.5, 123.2, 121.7, 120.9, 120.1, 77.3, 77.2, 77.0, 32.1, 30.9, 29.8, 29.8, 29.8, 29.7, 29.5, 26.4, 22.9, 14.3.

Compound S4

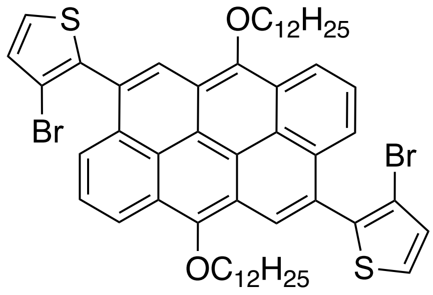

S2 (1.50 g, 1.55 mmol), 2,3-dibromothiophene (1.50 g, 6.20 mmol), $Pd(PPh_3)_4$ (179 mg, 0.16 mmol) and K_2CO_3 (0.86 g, 6.2 mmol) were added to a 250 mL Schlenk flask under argon, dissolved in dioxane/water (28/7 ml) and the mixture was deaerated by bubbling with argon for 10 minutes. The mixture was then refluxed at 100 °C for 20 h, cooled down to room temperature before 100 mL of water were added to the mixture. The crude product was filtered and washed with methanol and acetone. **S4** was purified with flash column chromatography using silica (CH_2Cl_2-Hex, 4:10) obtaining the pure product as a yellowish-orange solid, (58% yield, 0.87 g).

^{1}H NMR (700 MHz, $CDCl_3$) δ 8.84 (t, 2H), 8.57 (s, 2H), 8.17 (s, 2H), 8.16 (d, J = 1.4 Hz, 2H), 7.56 (d, J = 5.5 Hz, 2H), 7.28 (d, J = 5.5 Hz, 2H), 4.42 (t, J = 6.6 Hz, 4H), 2.15 (p, J = 6.8 Hz, 4H), 1.72 (p, J = 7.6 Hz, 4H), 1.47 (p, J = 7.3 Hz, 4H), 1.38 (p, J = 7.2 Hz, 4H), 1.35 – 1.21 (m, 27H), 0.89 (t, J = 7.0 Hz, 6H).

^{13}C{^{1}H} NMR (176 MHz, $CDCl_3$) δ 150.5, 137.0, 130.9, 130.4, 126.6, 126.4, 126.1, 126.1, 124.9, 123.8, 121.4, 121.2, 120.2, 111.6, 32.1, 30.9, 29.9, 29.8, 29.8, 29.8, 29.8, 29.5, 26.4, 22.9, 14.3.

Compound S5

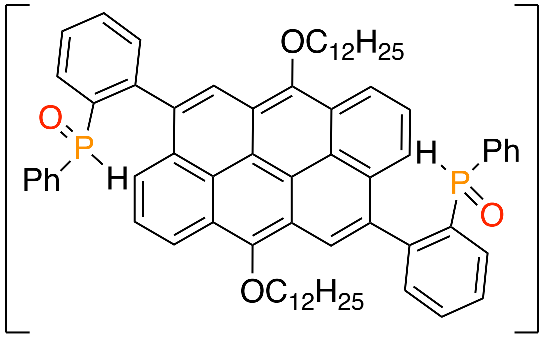

S3 (1.0 g, 1.05 mmol) was added to a 250 mL Schlenk flask with 150 mL of degassed THF under argon and the temperature was reduced to -78 °C. *n*-BuLi (0.92 ml, 2.30 mol, 2.50 M) was slowly added to the mixture and stirred for 1 h at low temperature. Next, $PhPCl_2$ (0.30 ml, 2.20 mmol) was added to the mixture and stirred for 3 h. Then, 7 mL of H_2O were added to the mixture, and it was stirred for 1 h. The solvent was evaporated under reduced pressure, the crude was re-dissolved in CH_2Cl_2, and the organic phase washed with water. The organic phase was dried using $MgSO_4$, the solvent was evaporated under vacuum. The product **S5** was obtained as a reddish crude, which was used for the next step without further purification.

Compounds *trans*-1-O and *cis*-1-O

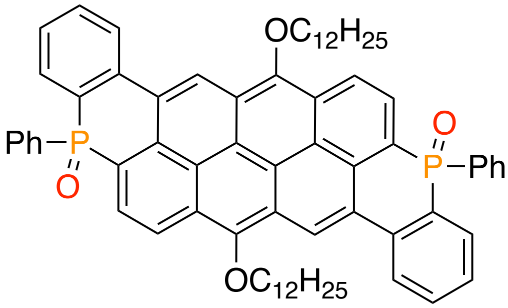

The crude containing **S5** from the previous step was dissolved in a 100 mL round-bottom flask under air with a mixture of toluene/acetonitrile (1:1, 20 mL) and wrapped with aluminum foil. Then, the reaction flask was charged with silver nitrate (0.04 g, 0.24 mmol), capped and stirred at

90 °C for 12 h. The reaction was allowed to reach room temperature and the solvent was evaporated under reduced pressure. ***trans*-1-O** and ***cis*-1-O** were purified by flash column chromatography using silica ($CHCl_3$-Acetone, 10:1, *trans*-isomer elutes first) and then recrystallized in a mixture of CH_2Cl_2 and hexane at -10 °C, obtaining the pure compounds as orange solids. The calculated reaction yields are for the overall transformations from **S3** and to ***trans*-1-O** or ***cis*-1-O** (e and f steps of Scheme 1, main text).

Note: The *trans*- and *cis*-isomers are formed in an uneven ratio (confirmed by ^{31}P NMR{^{1}H} of the crude mixture). Also, due to the lower solubility of the *trans*-isomer, it precipitates preferentially during the recrystallization process.

Trans-isomer ***trans*-1-O**: (14.5 %, 0.16 g). **^{1}H NMR** (700 MHz, $CDCl_3$) δ 9.61 (s, 2H), 8.97 (d, *J* = 8.3 Hz, 2H), 8.74 – 8.65 (m, 4H), 8.17 (dd, *J* = 12.2, 6.8 Hz, 2H), 7.83 (t, 2H), 7.73 – 7.67 (m, 4H), 7.63 (t, *J* = 7.2 Hz, 2H), 7.42 (td, *J* = 7.3, 1.4 Hz, 2H), 7.37 (td, *J* = 7.6, 3.0 Hz, 4H), 4.50 – 4.42 (m, 4H), 2.25 – 2.16 (m, 4H), 1.85 – 1.80 (m, 4H), 1.53 (p, *J* = 7.4 Hz, 4H), 1.49 – 1.41 (m, 4H), 1.40 – 1.34 (m, 5H), 1.34 – 1.21 (m, 24H), 0.88 (t, *J* = 7.1 Hz, 6H).
^{31}P{^{1}H} NMR (162 MHz, $CDCl_3$) δ ppm 9.2.
^{13}C{^{1}H} NMR (176 MHz, $CDCl_3$) δ 152.4, 138.1 (d, *J* = 7.0 Hz) , 135.2 (d, *J* = 110.9 Hz), 132.6, 132.5 (d, *J* = 7.0 Hz), 131.7 (d, *J* = 3.5 Hz), 131.3 (d, *J* = 10.6 Hz), 131.0 (d, *J* = 5.3 Hz), 129.0 (d, *J* = 10.6 Hz), 128.9 (d, *J* = 10.6 Hz), 128.7 (d, *J* = 12.3 Hz), 128.6 (d, *J* = 102.1 Hz), 128.4 (d, *J* = 7.0 Hz), 127.9, 125.5 (d, *J* = 8.8 Hz), 124.4 (d, *J* = 10.6 Hz), 123.9 (d, *J* = 102.1 Hz), 122.5, 122.4, 122.3, 119.4, 78.3, 32.1, 31.0, 30.0, 29.9, 29.8, 29.8, 29.8, 29.5, 26.8, 22.8, 14.3.
^{13}C{^{1}H} DEPT 135 NMR (176 MHz, $CDCl_3$) δ 132.5, 132.4 (d, *J* = 7.0 Hz), 131.6, 131.2 (d, *J* = 10.6 Hz), 128.9 (d, *J* = 10.6 Hz), 128.6 (d, *J* = 14.1 Hz), 128.2 (d, *J* = 7.0 Hz), 125.4 (d, *J* = 8.8 Hz), 122.3, 122.2, 78.2, 31.9, 30.9, 29.8, 29.7, 29.7, 29.7, 29.7, 29.4, 26.7, 22.7, 14.1. E.A. (%) calcd for $C_{70}H_{74}O_4P_2$ (1041.31): C 80.74, H 7.16. Found: C 80.21, H 7.29.

Cis-isomer ***cis*-1-O**: (11.0 %, 0.12 g). **^{1}H NMR** (400 MHz, $CDCl_3$) δ 9.52 (s, 2H), 8.94 (d, *J* = 8.5 Hz, 2H), 8.74 (dd, *J* = 11.9, 8.4 Hz, 2H), 8.63 (dd, *J* = 8.4, 5.3 Hz, 2H), 8.18 (dd, *J* = 13.0, 7.6 Hz, 2H), 7.83 (t, *J* = 7.8 Hz, 2H), 7.66 – 7.55 (m, 6H), 7.43 – 7.33 (m, 2H), 7.32 – 7.27 (m, 4H), 4.44

(t, J = 6.4 Hz, 4H), 2.27 – 2.15 (m, 4H), 1.84 (p, J = 6.9 Hz, 4H), 1.61 – 1.51 (m, 4H), 1.50 – 1.42 (m, 4H), 1.41 – 1.17 (m, 24H), 0.88 (t, J = 6.9 Hz, 6H).

^{31}P{^{1}H} NMR (162 MHz, $CDCl_3$) δ ppm 8.8.

^{13}C{^{1}H} NMR (101 MHz, $CDCl_3$) δ 152.3, 137.9 (d, J = 6.1 Hz), 135.7 (d, J = 110.1 Hz), 132.7, 132.2 (d, J = 6.1 Hz), 131.5 (d, J = 2.0 Hz), 131.0 (d, J = 10.1 Hz), 130.8 (d, J = 7.1 Hz), 129.0, 128.9 (d, J = 5.1 Hz), 128.6 (d, J = 12.1 Hz), 128.4 (d, J = 86.9 Hz), 128.1 (d, J = 6.1 Hz), 127.7 (d, J = 2.0 Hz), 125.6 (d, J = 10.1 Hz), 124.2 (d, J = 6.1 Hz), 123.7 (d, J = 99.0 Hz), 122.3, 122.2, 122.2, 119.1, 78.2, 32.1, 31.0, 30.0, 29.9, 29.8, 29.5, 26.8, 22.8, 14.3. E.A. (%) calcd for $C_{70}H_{74}O_4P_2$ (1041.31): C 80.74, H 7.16. Found: C 79.79, H 7.12.

Compound S6

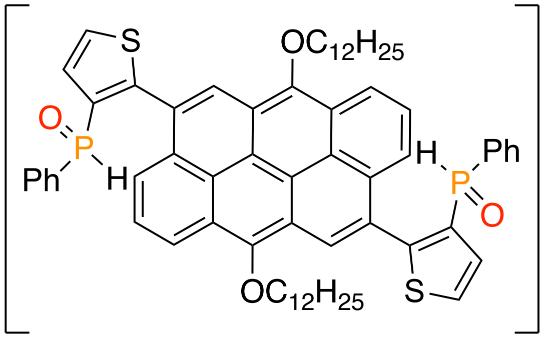

S4 (1.0 g, 1.03 mmol) was added to a 250 mL Schlenk flask with 150 mL degassed THF under argon and the temperature was reduced to -78 °C. *n*-BuLi (0.91 ml, 2.27 mol, 2.50 M) was slowly added to the mixture and stirred for 1 h at low temperature. Next, $PhPCl_2$ (0.29 ml, 2.17 mmol) was added to the mixture and stirred for 3 h. Then, 7 mL of H_2O were added to the mixture and stirred for 45 minutes. The solvent was evaporated under reduced pressure, the crude was re-dissolved in CH_2Cl_2, and the organic phase was washed with water. The organic phase was then dried using $MgSO_4$ and the solvent was evaporated, obtaining **S6** as a reddish crude, which was used for the next step without further purification.

Compounds *trans*-2-O and *cis*-2-O

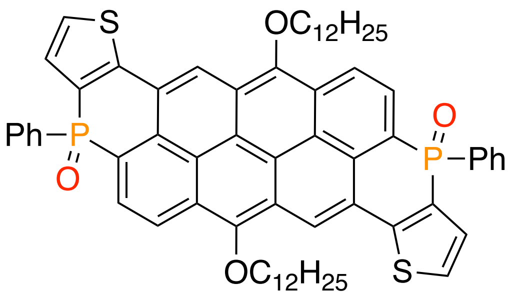

The crude containing **S6** from the previous step was dissolved in a 100 ml round-bottom flask under air with a mixture of toluene/acetonitrile (1:1, 20 ml) and wrapped with aluminum foil under air. Then, the mixture was charged with silver nitrate (0.04 g, 0.24 mmol), capped and stirred at 90 °C for 12h. The reaction was allowed to reach room temperature and the solvent was evaporated under reduced pressure. ***trans*-2-O** and ***cis*-2-O** were purified by flash column chromatography using silica ($CHCl_3$-acetone, 10:1, *trans*-isomer comes off first) and then recrystallized in a mixture of CH_2Cl_2 and hexane at -10 °C, obtaining the pure products as orange solid. The calculated reaction yields are from overall transformations from **S4** and to ***trans*-2-O** or ***cis*-2-O** (e and f steps of scheme 1, main text).

Note: The *trans*- and *cis*-isomers are formed in an uneven ratio (proved by ^{31}P NMR{^{1}H} of the crude mixture). Due to the lower solubility of *trans*-isomer, it precipitates more during the recrystallization process.

Trans-isomer ***trans*-2-O**: (13 %, 0.14 g) **^{1}H NMR** (400 MHz, $CDCl_3$) δ ppm 9.26 (s, 2H), 8.92 (d, *J* = 8.4 Hz, 2H), 8.62 (dd, *J* = 12.2, 8.5 Hz, 2H), 7.77 (dd, *J* = 13.0, 7.2 Hz, 4H), 7.61 – 7.52 (m, 4H), 7.51 – 7.46 (m, 2H), 7.46 – 7.38 (m, 4H), 4.43 (t, *J* = 5.6 Hz, 4H), 2.27 – 2.12 (m, 4H), 1.94 – 1.77 (m, 4H), 1.60 – 1.50 (m, 4H), 1.50 – 1.42 (m, 4H), 1.41 – 1.16 (m, 25H), 0.88 (t, *J* = 6.7 Hz, 6H).

^{31}P{^{1}H} NMR (162 MHz, $CDCl_3$) δ ppm 7.8.

^{13}C{^{1}H} NMR (101 MHz, $CDCl_3$) δ 152.5, 149.7 (d, *J* = 10.1 Hz), 134.9 (d, *J* = 113.1 Hz), 132.0 (d, *J* = 3.0 Hz), 131.5 (d, *J* = 11.1 Hz), 130.2, 129.9 (d, *J* = 11.1 Hz), 129.5 (d, *J* = 6.1 Hz), 129.1 (d, *J* = 3.0 Hz), 128.8 (d, *J* = 12.1 Hz), 128.4 (d, *J* = 133.3 Hz), 127.1 (d, *J* = 16.2 Hz), 126.7 (d, *J*

= 10.1 Hz), 125.4 (d, J = 104.0 Hz), 124.1 (d, J = 10.1 Hz), 122.7 (d, J = 12.1 Hz), 122.5, 122.3, 119.4, 78.5, 32.1, 30.9, 29.9, 29.8, 29.5, 26.7, 22.8, 14.3.

$^{13}C\{^1H\}$ DEPT 135 NMR (101 MHz, $CDCl_3$) δ 131.8, 131.3 (d, J = 10.1 Hz), 129.7 (d, J = 12.1 Hz), 128.9 (d, J = 7.1 Hz), 128.6 (d, J = 12.1 Hz), 126.9 (d, J = 17.2 Hz), 122.5 (d, J = 13.1 Hz), 122.3, 78.3, 31.9, 30.8, 29.7, 29.7, 29.6, 29.3, 26.5, 22.6, 14.1. E.A. (%) calcd for $C_{66}H_{70}O_4P_2S_2$ (1053.35): C 75.26, H 6.70. Found: C 71.15, H 6.56.[S3,S4]

Cis-isomer ***cis*-2-O**: (6.5 % , 0.07 g) **^1H NMR** (400 MHz, $CDCl_3$) δ 9.20 (s, 2H), 8.89 (d, J = 8.5 Hz, 2H), 8.62 (dd, J = 12.2, 8.5 Hz, 2H), 7.75 (dd, J = 12.7, 7.0 Hz, 4H), 7.60 – 7.51 (m, 4H), 7.50 – 7.44 (m, 2H), 7.43 – 7.36 (m, 4H), 4.47 – 4.33 (m, 4H), 2.26 – 2.10 (m, 4H), 1.89 – 1.76 (m, 4H), 1.61 – 1.50 (m, 4H), 1.49 – 1.41 (m, 4H), 1.40 – 1.11 (m, 26H), 0.88 (t, 6H).

$^{31}P\{^1H\}$ NMR (162 MHz, $CDCl_3$) δ ppm 7.5.

$^{13}C\{^1H\}$ NMR (101 MHz, $CDCl_3$) δ 152.2, 149.7 (d, J = 10.1 Hz), 134.8 (d, J = 113.1 Hz), 131.7 (d, J = 2.0 Hz), 131.4 (d, J = 11.1 Hz), 130.1, 129.8 (d, J = 11.1 Hz), 129.0, 129.0, 128.7 (d, J = 13.1 Hz), 128.1 (d, J = 170.7 Hz), 127.0 (d, J = 16.2 Hz), 126.1 (d, J = 10.1 Hz), 125.1 (d, J = 104.0 Hz), 123.6 (d, J = 10.1 Hz), 122.7 (d, J = 12.1 Hz), 122.3, 121.8, 118.8, 78.3, 32.0, 30.8, 29.9, 29.9, 29.8, 29.5, 26.7, 22.8, 14.2. E.A. (%) calcd for $C_{66}H_{70}O_4P_2S_2$ (1053.35): C 75.26, H 6.70. Found: C 74.77, H 6.74.

Compounds 1

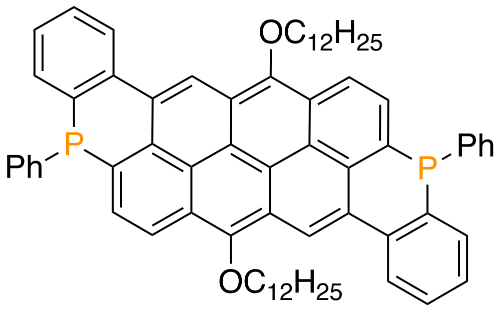

A mixture of ***trans*-1-O** and ***cis*-1-O** (1:1, 30 mg) was dissolved in 20 ml of THF in a microwave vial under inert atmosphere and reacted with $HSiCl_3$ (100 eq, 2.97 mmol, 0.30 ml) under microwave irradiation at 150 °C for 20 minutes. The mixture was allowed to cool to room

temperature, then transferred into a Schlenk flask and the solvent was evaporated under reduced pressure. The crude mixture containing **1** was recrystalized with DCM/pentane at -30 °C, obtaining a mixture of isomers. (77 % yield, 22 mg).

Note: Due to the inability of separating the isomers ***trans*-1** and ***cis*-1**, the ^{1}H, ^{31}P{^{1}H} and ^{13}C{^{1}H} NMR spectra of the mixture containing both isomers have been reported in here. In case of the ^{1}H NMR and ^{13}C{^{1}H} NMR data, precise peak assignments for the respective isomers are improbable as the chemical shifts appear too close to each other.

^{1}H NMR (400 MHz, $CDCl_3$) δ 9.25 (s, 2H), 8.69 (d, *J* = 8.5 Hz, 2H), 8.52 (d, *J* = 8.2 Hz, 2H), 8.13 (dd, *J* = 10.6, 8.3 Hz, 2H), 7.65 – 7.55 (m, 4H), 7.55 – 7.44 (m, 4H), 7.38 (t, *J* = 7.4 Hz, 2H), 7.32 – 7.26 (m, 4H), 4.39 (t, *J* = 6.4 Hz, 4H), 2.24 – 2.12 (m, 4H), 1.83 (p, *J* = 7.1 Hz, 4H), 1.60 – 1.48 (m, 4H), 1.48 – 1.40 (m, 4H), 1.40 – 1.16 (m, 32H), 0.87 (t, 8H).
^{31}P{^{1}H} NMR (162 MHz, $CDCl_3$) δ ppm -27.2 (*trans*), -27.5 (*cis*).
^{13}C{^{1}H} NMR (101 MHz, $CDCl_3$) δ 150.1, 141.1 (d, *J* = 24.2 Hz), 136.5, 134.6, 134.4 (d, *J* = 21.2 Hz), 134.3, 134.3 (d, *J* = 20.2 Hz), 133.8 (d, *J* = 4.0 Hz), 131.6, 130.9 (d, *J* = 32.3 Hz), 130.1, 129.5 (d, *J* = 10.1 Hz), 128.8 (d, *J* = 8.1 Hz), 127.9 (d, *J* = 11.1 Hz), 126.2, 125.7, 125.0, 122.5, 120.6 (d, *J* = 11.1 Hz), 120.3, 118.7, 77.4, 32.1, 31.1, 30.0, 29.9, 29.9, 29.9, 29.8, 29.5, 26.9, 22.9, 14.3. E.A. (%) calcd for $C_{70}H_{74}O_2P_2$ (1009.31): C 83.30, H 7.39. Found: C 79.58, H 7.30.[S3,S4]

Compounds 2

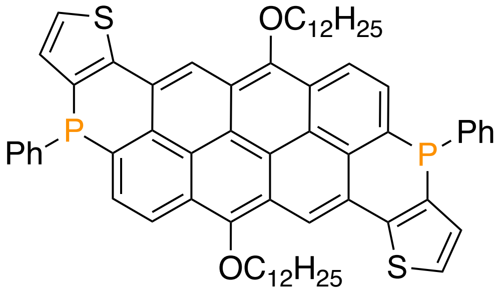

A mixture of ***trans*-2-O** and ***cis*-2-O** (1:1, 40 mg) was dissolved in 15 ml THF in a microwave vial under inert atmosphere and reacted with $HSiCl_3$ (100 eq, 3.92 mmol, 0.40 ml) under microwave irradiation at 150 °C for 20 minutes. The mixture was allowed to cool down, transferred into a

Schlenk flask and the solvent was evaporated under reduced pressure. **2** crude mixture was recrystalized with DCM/pentane in -30 °C, obtaining a mixture of isomers with uneven ratio. (71 % yield, 28 mg).

Note: Due to the inability of separating the isomers ***trans*-2** and ***cis*-2**, the ^{1}H, ^{31}P{^{1}H} and ^{13}C{^{1}H} NMR spectra of the mixture containing both isomers have been reported in here. In case of the ^{1}H NMR and ^{13}C{^{1}H} NMR data, precise peak assignments for the respective isomers are improbable as the chemical shifts appear too close to each other.

^{1}H NMR (400 MHz, $CDCl_3$) δ 8.80 (s, 2H), 8.66 (d, *J* = 8.7 Hz, 2H), 8.15 (dd, *J* = 11.4, 8.5 Hz, 2H), 7.58 – 7.49 (m, 4H), 7.44 (dd, *J* = 5.2, 2.6 Hz, 2H), 7.33 – 7.26 (m, 6H), 7.13 (dd, *J* = 5.2, 2.8 Hz, 2H), 4.36 (t, *J* = 6.5 Hz, 4H), 2.22 – 2.12 (m, 4H), 1.84 (p, *J* = 7.1 Hz, 4H), 1.54 (p, *J* = 6.9 Hz, 4H), 1.45 (p, *J* = 7.1 Hz, 5H), 1.41 – 1.17 (m, 30H), 0.88 (t, *J* = 6.9 Hz, 7H).

^{31}P{^{1}H} NMR (162 MHz, $CDCl_3$) δ ppm -26.6 (*trans*), -26.8 (*cis*).

^{13}C{^{1}H} NMR (101 MHz, $CDCl_3$) δ 149.7, 142.7, 139.9 (d, *J* = 27.3 Hz), 134.0 (d, *J* = 21.2 Hz), 132.9, 131.7 (d, *J* = 29.3 Hz), 131.6 (d, *J* = 35.3 Hz), 129.7, 128.9 (d, *J* = 8.1 Hz), 128.5, 126.3, 125.9 (d, *J* = 9.1 Hz), 124.6, 122.5, 120.8, 120.7, 117.7, 77.4, 32.1, 31.0, 29.9, 29.9, 29.6, 26.8, 22.9, 14.3. E.A. (%) calcd for $C_{66}H_{70}O_2P_2S_2$ (1021.35): C 77.62, H 6.91 Found: C 60.86, H 5.32.[S3,S4]

Compounds 1-Me

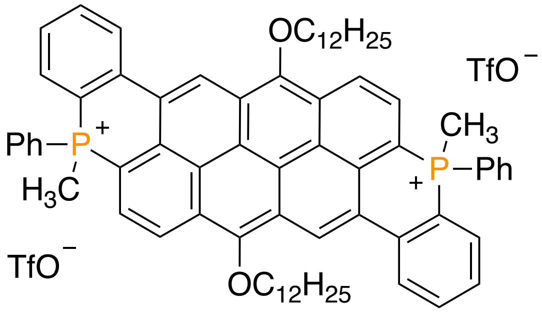

Compound **1** (30 mg) was dissolved in 10 ml of dry CH_2Cl_2 in a 25 mL Schlenk flask under argon and the temperature was cooled to 0 °C. MeOTf (100 eq, 2.97 mmol ,0.33 mL) was slowly added to the reaction mixture and it was stirred overnight at room temperature. Then, the solvent was evaporated under reduced pressure and the mixture **1-Me** was purified by recrystallization from a small amount of ethanol at -10 °C, obtaining a mixture of isomers (86 % yield, 34 mg).
Note: Due to the inability of separating the isomers ***trans*-1-Me** and ***cis*-1-Me**, the ^{1}H, ^{31}P{^{1}H} and ^{13}C{^{1}H} NMR spectra of the mixture containing both isomers have been reported in here. In case of the ^{1}H NMR and ^{13}C{^{1}H} NMR data, precise peak assignments for the respective isomers are improbable as the chemical shifts appear too close to each other.

^{1}H NMR (400 MHz, $CDCl_3$) δ 9.46 (s, 2H), 9.27 (s, 2H), 8.94 – 8.69 (m, 10H), 8.50 (d, *J* = 8.4 Hz, 2H), 8.38 – 8.30 (m, 2H), 8.29 – 8.24 (m, 2H), 8.23 – 8.14 (m, 5H), 8.11 – 8.00 (m, 9H), 7.99 – 7.91 (m, 4H), 7.84 (dd, *J* = 14.0, 7.8 Hz, 4H), 7.76 (dd, *J* = 7.2, 1.9 Hz, 2H), 7.74 – 7.67 (m, 4H), 7.63 (dd, *J* = 7.3, 1.9 Hz, 2H), 7.60 – 7.51 (m, 4H), 4.37 – 4.19 (m, 4H), 4.10 (q, *J* = 6.6 Hz, 2H), 4.02 (q, *J* = 7.8 Hz, 2H), 3.52 (d, *J* = 13.6 Hz, 6H), 3.20 (d, *J* = 13.6 Hz, 6H), 2.09 – 1.98 (m, 4H), 1.93 – 1.75 (m, 4H), 1.67 – 1.57 (m, 5H), 1.50 – 1.15 (m, 80H), 0.84 (t, 14H).
^{31}P{^{1}H} NMR (162 MHz, $CDCl_3$) δ ppm -0.1 (*trans*), -0.4 (*cis*).
^{13}C{^{1}H} NMR (101 MHz, $CDCl_3$) δ 153.5, 152.8, 138.6, 138.6, 138.5, 136.2, 136.0, 135.3, 135.1, 133.8, 133.7, 133.02 (d, *J* = 11.1 Hz), 132.6 (d, *J* = 11.1 Hz), 131.7, 131.7, 131.5, 131.3, 131.2, 131.1, 131.1, 130.8 (d, *J* = 14.1 Hz), 130.6 (d, *J* = 13.1 Hz), 129.5, 129.1 (d, *J* = 10.1 Hz), 128.7, 128.0, 127.0 (d, *J* = 12.1 Hz), 126.8 (d, *J* = 13.1 Hz), 124.3, 123.9, 123.8, 123.3, 123.2 (d, *J* = 11.1 Hz), 122.7, 122.6, 122.4, 122.4, 120.5 (q, *J* = 321.2 Hz) , 118.5, 117.8, 114.0, 113.9, 113.2, 113.0, 109.7, 109.6, 108.8, 108.8, 79.5, 79.4, 32.0, 30.8, 30.6, 29.9, 29.9, 29.8, 29.8, 29.7, 29.6, 29.5, 26.5, 26.3, 22.8, 14.2, 12.3 (d, *J* = 59.6 Hz), 12.0 (d, *J* = 59.6 Hz). E.A. (%) calcd for $C_{74}H_{80}O_8P_2S_2F_6$ (1337.5): C 66.45, H 6.04 Found: C 63.56, H 5.96.[S3,S4]

Compounds 2-Me

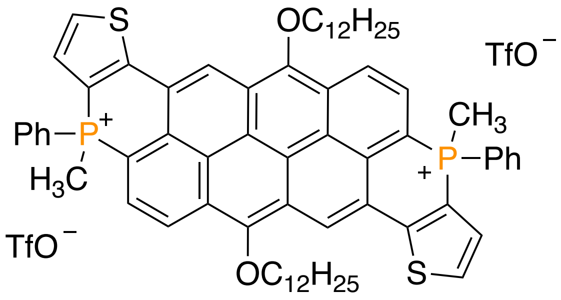

Compound **2** (25 mg) was dissolved in 10 ml of CH_2Cl_2 in a 25 ml Schlenk flask under argon and the temperature was cooled down to 0 °C. MeOTf (100 eq, 2.45mmol ,0.27 ml) was slowly added to the reaction mixture and it was stirred overnight at room temperature. Then, the solvent was evaporated under reduced pressure and the mixture **2-Me** purified by recrystallization from a small amount of ethanol at -10 °C, obtaining a mixture of isomers (82 % yield, 27 mg).

Note: Due to the inability of separating the isomers ***trans*-2-Me** and ***cis*-2-Me**, the ^{1}H, $^{31}P\{^{1}H\}$ and $^{13}C\{^{1}H\}$ NMR spectra of the mixture containing both isomers have been reported in here. In case of the ^{1}H NMR and $^{13}C\{^{1}H\}$ NMR data, precise peak assignments for the respective isomers are improbable as the chemical shifts appear too close to each other.

^{1}H NMR (400 MHz, $CDCl_3$) δ 9.05 (dd, *J* = 14.2, 8.6 Hz, 2H), 8.84 (s, 2H), 8.55 (s, 2H), 8.26 – 8.06 (m, 2H), 7.97 (dd, *J* = 14.3, 7.7 Hz, 4H), 7.83 – 7.67 (m, 5H), 7.66 – 7.56 (m, 3H), 4.53 – 4.15 (m, 1H), 3.92 (q, *J* = 7.7 Hz, 2H), 3.83 (q, *J* = 8.7 Hz, 2H), 3.61 (d, *J* = 14.0 Hz, 5H), 3.23 (d, *J* = 13.8 Hz, 1H), 2.11 – 1.94 (m, 1H), 1.85 – 1.68 (m, 3H), 1.50 – 1.05 (m, 40H), 0.85 (t, *J* = 6.6 Hz, 7H).

^{31}P{^{1}H} NMR (162 MHz, $CDCl_3$) δ ppm -1.4 (*trans*), -1.9 (*cis*).

^{13}C{^{1}H} NMR (101 MHz, $CDCl_3$) δ 153.4 (d, *J* = 8.1 Hz), 152.2, 135.4, 133.6 (d, *J* = 12.1 Hz), 132.8 (d, *J* = 13.1 Hz), 132.4 (d, *J* = 12.1 Hz), 130.9, 130.7, 128.9 (d, *J* = 5.0 Hz), 128.5, 127.2, 124.9 (d, *J* = 10.1 Hz), 123.7, 122.7, 122.3 (d, *J* = 10.1 Hz), 121.8, 119.6, 119.4 (q, *J* = 348.4 Hz), 112.1, 111.4, 111.2, 110.5, 79.8, 32.0, 30.5, 29.8, 29.7, 29.7, 29.6, 29.5, 29.5, 25.9, 22.8, 14.2, 12.9 (d, *J* = 58.6 Hz). E.A. (%) calcd for $C_{70}H_{76}O_8P_2S_4F_6$ (1349.54): C 62.30, H 5.69 Found: C 53.64, H 5.75.[S3,S4]

Compound *cis*-2-O(BCF)$_2$

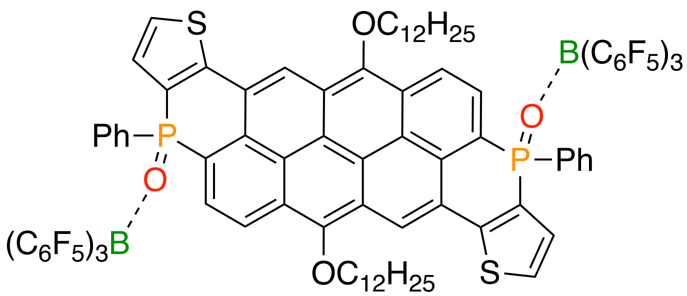

Compound ***cis*-2-O** (5 mg) was dissolved in a small volume of deuterated chloroform containing BCF (2 eq, 0.01 mmol, 4.9 mg) at room temperature. Quantitative transformation was monitored and verified by NMR.

^{1}H NMR (400 MHz, $CDCl_3$) δ 9.38 (s, 2H), 9.01 (dd, *J* = 8.6, 1.8 Hz, 2H), 8.45 (dd, *J* = 13.3, 8.6 Hz, 2H), 7.96 (dd, *J* = 15.1, 7.2 Hz, 4H), 7.77 – 7.69 (m, 2H), 7.67 – 7.57 (m, 6H), 7.45 (t, *J* = 4.9 Hz, 2H), 4.70 – 4.60 (m, 2H), 4.59 – 4.48 (m, 2H), 3.75 (s, 2H), 2.35 – 2.19 (m, 4H), 2.00 – 1.79 (m, 6H), 1.59 (p, 4H), 1.49 (p, *J* = 6.6 Hz, 4H), 1.43 – 1.22 (m, 24H), 0.87 (t, 6H).
^{31}P{^{1}H} NMR (162 MHz, $CDCl_3$) δ ppm 19.2.

Scheme S1. Details of the reaction mechanism toward the formation of the **S5** and **S6.**

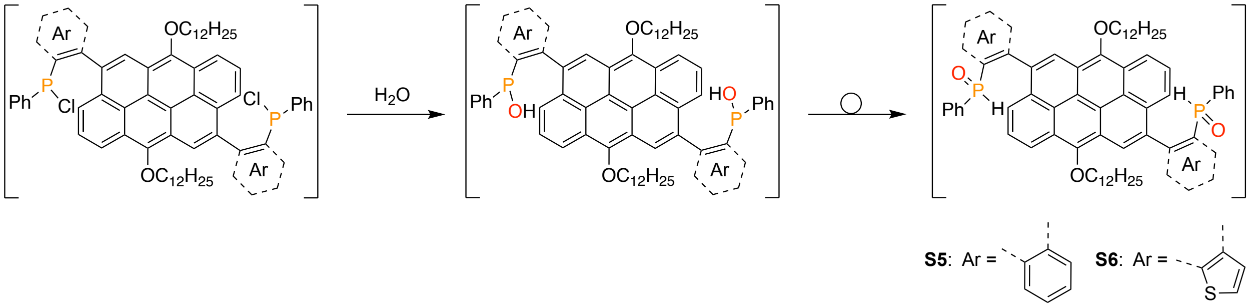

3. X-ray diffraction data

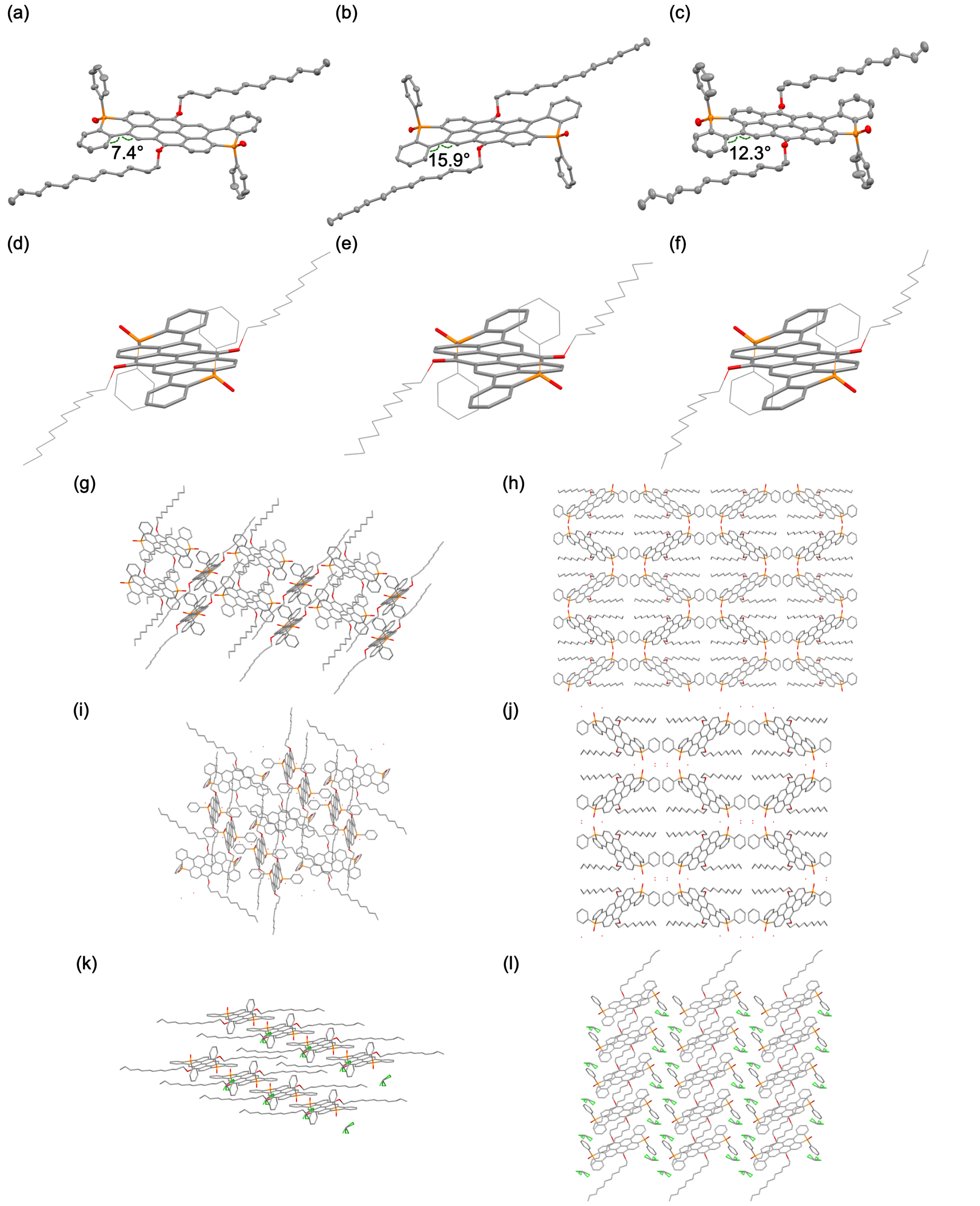

Figure S1. Structures of ***trans*-1-O** in the solid state without crystallized solvate (a), with water (b), and with chloroform (c) along with their respective twist (d, e, f). Packing of ***trans*-1-O** (g, h: with no crystallized solvate), (i, j: with water), (k, l: with chloroform).

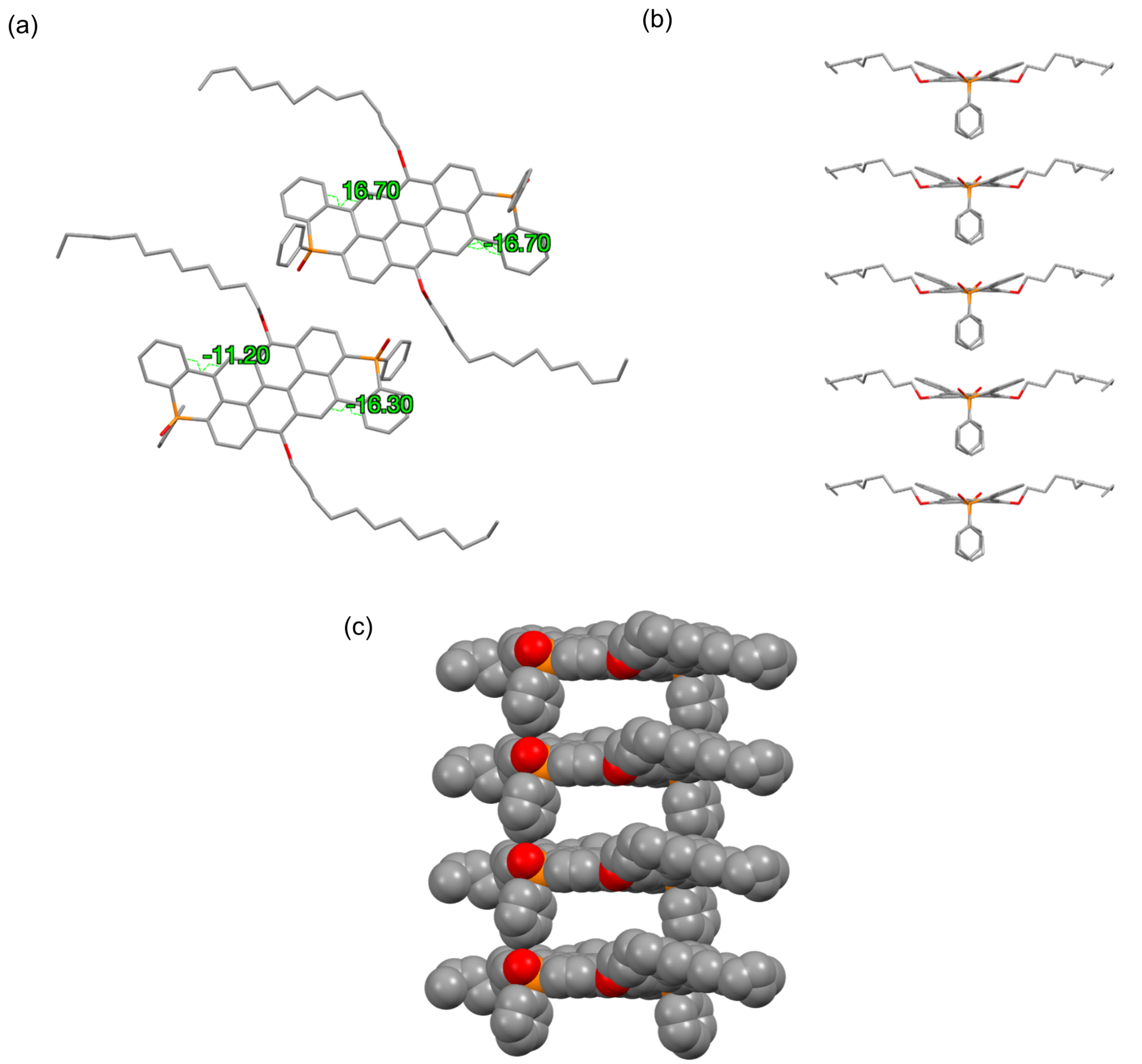

Figure S2. Unit cell of ***cis*-1-O** showing both enantiomers with their torsion angles (a). Packing of ***cis*-1-O** (b), and space filling view (c). H-atoms omitted for clarity.

(a)

(b)

(c)

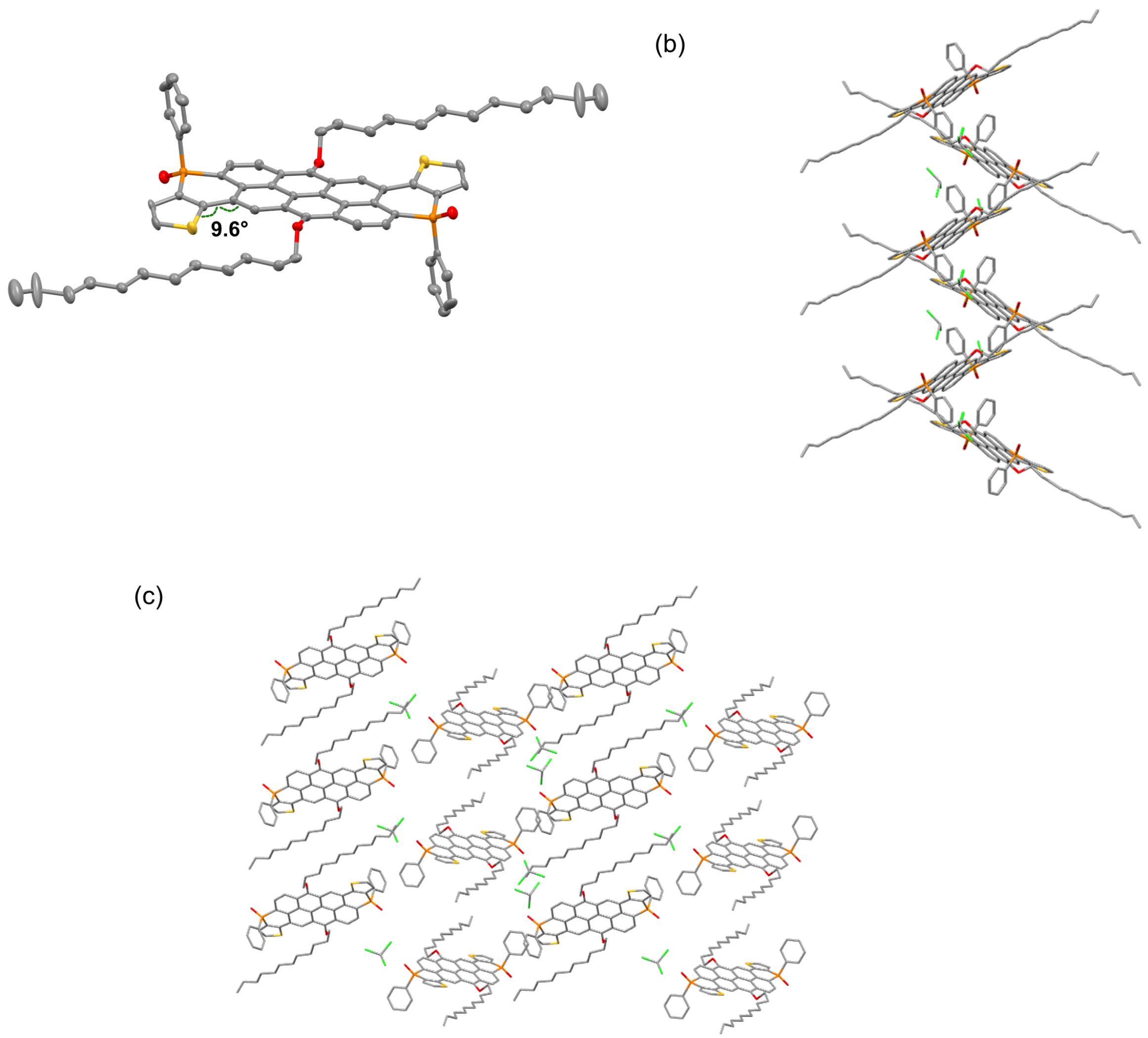

Figure S3. Molecular structure (a), and packing of ***trans*-2-O** side and front view (b,c). H-atoms omitted for clarity.

4. Theoretical calculations

4.1. Computational Details

The computational methodology adopted in this study involved a series of steps to comprehensively analyze the electronic and aromatic properties of the new compounds. All calculations were performed at the B3LYP[S5]/def2-TZVP[S6]/CPCM(CH_2Cl_2)[S7] level of theory, unless further specified. All 12 compounds were subjected to structural optimizations, followed by Hessian calculations to confirm the optimized structures are minima on their ground-state potential energy surfaces. TD-DFT calculations were then performed at the optimized structures to calculate vertical excited states and vertical excitation energies. The calculated excitation energies and oscillator strengths enable the assignment of peaks in experimental absorption spectra and explanation of the absorption intensities. Nucleus-Independent Chemical Shifts[S8] were calculated at 1.0 Å above (i.e., NICS$(1)_{zz}$) rings of interest of the optimized structures to estimate their aromaticities or antiaromaticities, at the ωB97X[S9]/cc-pVTZ level. The key calculated results are summarized in Table S1.

4.2. Summary of Calculated Results

Table S1. Experimental and calculated absorption maxima obtained by TD-DFT calculations and NICS values obtained for all derivatives from series **1** and **2**.

Compd	λ_{abs}[a] (calc)	λ_{abs}[b] (expt)	f_{osc}[c] (calc)	ε[d] (expt)	NICS(1)$_{ZZ}$ in ppm[e]				
					R_1	R_2	R_3	R_4	R_5
***Trans*-1-O**	361, 496	356, 509	0.33, 0.17	94K	-23.7	12.6	-31.2	-26.2	-5.0
***Cis*-1-O**	360, 496	356, 509	0.33, 0.17	59K	-23.9	11.3	-30.7	-26.1	-4.7
***Trans*-1**	376, 507	369, 510	0.30, 0.23	134K	-22.9	12.1	-31.1	-26.2	-4.1
***Cis*-1**	377, 506	369, 510	0.23, 0.20	134K	-22.7	13.5	-30.1	-26.0	-3.8
***Trans*-1-Me**	337, 558	363, 533	0.35, 0.23	88K	-22.7	11.4	-26.6	-23.3	-5.5
***Cis*-1-Me**	366, 506	363, 533	0.33, 0.19	88K	-23.2	11.5	-29.9	-25.5	-3.8
***Trans*-2-O**	350, 507	366, 517	0.22, 0.16	40K	-19.2	11.7	-31.5	-26.0	-4.6
***Cis*-2-O**	349, 509	367, 517	0.22, 0.15	62K	-19.3	10.0	-31.4	-25.9	-4.5
***Trans*-2**	386, 516	376, 514	0.23, 0.20	67K	-19.2	10.7	-31.1	-25.9	-3.5
***Cis*-2**	386, 516	376, 514	0.20, 0.18	67K	-19.1	12.0	-30.7	-25.6	-3.4
***Trans*-2-Me**	363, 572	373, 540	0.50, 0.18	57K	-18.6	11.3	-29.5	-26.0	-6.8
***Cis*-2-Me**	364, 572	373, 540	0.51, 0.20	57K	-18.8	11.1	-29.6	-25.9	-6.7

[a] Calculated absorption maxima. [b] Experimental absorption maxima. [c] Oscilator strength. [d] Experimental molar extinction coefficient. [e] Calculated NICS(1) values in ppm. See assignments below.

4.3. NICS Results and Aromaticities of the series 1 and 2.

For each compound of the series **1** and **2**, NICS(1)$_{zz}$ calculations were performed for the 5 rings exemplified in the figure below, which are unique in the context of the inherent C_{2h} symmetry of the core planar structure. They are labeled as Rings 1 to 5, and abbreviated as R1 to R5 in the discussion below. All 60 calculated NICS(1)$_{zz}$ values are summarized in Table S1.

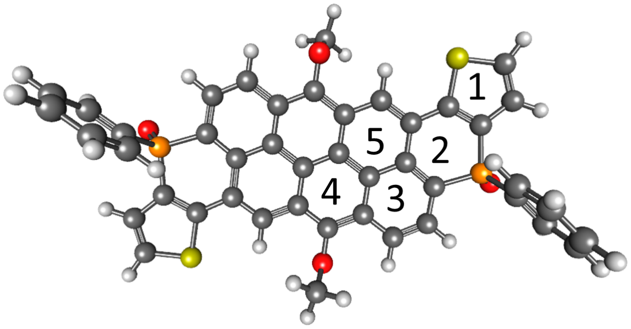

Labeling of rings in compound ***cis*-2-O**

The NICS(1)$_{zz}$ results are almost insensitive to the *cis* vs. *trans* configurations. R1 is aromatic in all compounds and the aromaticity is reduced in the replacement of the terminal benzo group by a thieno unit, as anticipated. R2 is antiaromatic in all compounds, likely due to the tetrahedral geometry of the P atom, and the antiaromaticity remains largely unchanged in all compounds. The P-containing rings also disconnect the electronic ring current between the central anthanthrene core, comprising R3-5 and their C_2 symmetry images, and the terminal rings R1 and its C_2 image. Notably, the NICS(1)$_{zz}$ values of R3-5 are consistent and follow the order of the values, -34, -29, and -12 ppm, of the corresponding rings of the free standing anthanthrene.[S10] Evidently, the aromaticity of the anthanthrene core is preserved in all the 12 compounds, despite the various modifications introduced to the system via π-expansion and additional phosphorus chemistry.

4.2. Results of TD-DFT Calculations and the Simulated Absorption Spectra

The two main absorption peaks of the TD-DFT-calculated and experimental absorption spectra of the 12 new compounds are compared in Table 1, in terms of their absorption wavelengths and intensities (calculated oscillator strengths vs. experimental extinction coefficients). The calculated absorption wavelengths (λ_{abs}) are all in close agreement with the experimental values. Notably, the calculated excitation energies and oscillator strengths are again insensitive to the *cis* or *trans* configurations of each isomer, respectively. The TD-DFT calculations allow us to assign excitation schemes for the observed peaks; see Figure S6 for ***cis*-2-O** as an example. The assignment also applies to the other 11 investigated compounds since the photophysics of all the compounds are similarly dominated by the anthanthrene core.

4.5. Frontier Molecular Orbitals

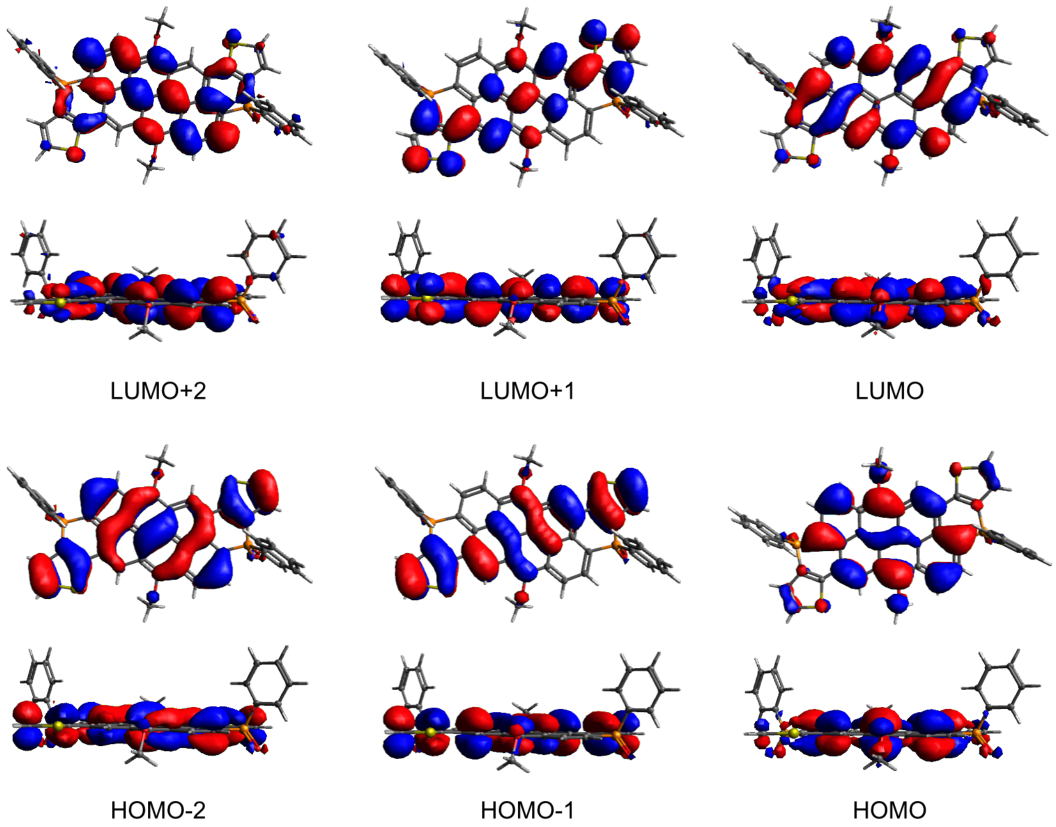

Figure S4. Frontier molecular orbitals of ***cis*-2-O** from HOMO-2 to LUMO+2. Both top and side views are presented for each orbital. They resemble frontier molecular orbitals of the other investigated species, which are hence not plotted.

Frontier molecular orbitals of the synthesized species; LUMO (top) and HOMO (bottom).

***trans*-1-O**

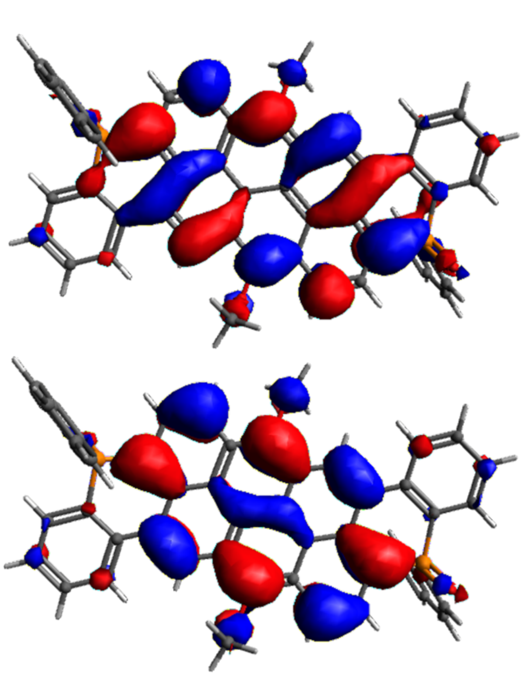

***cis*-1-O**

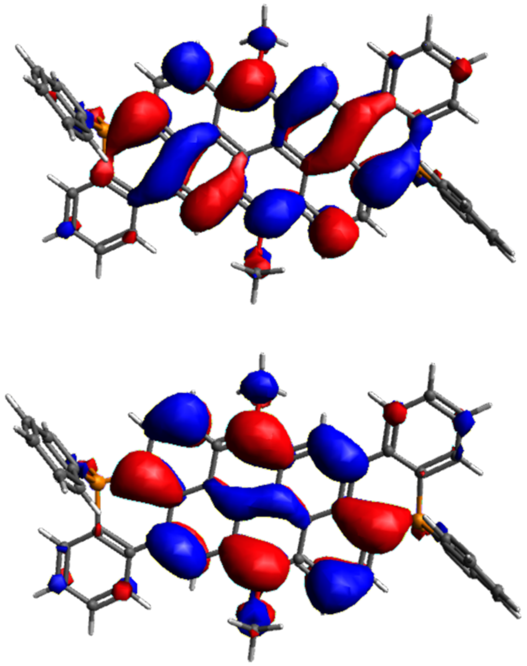

***trans*-1**

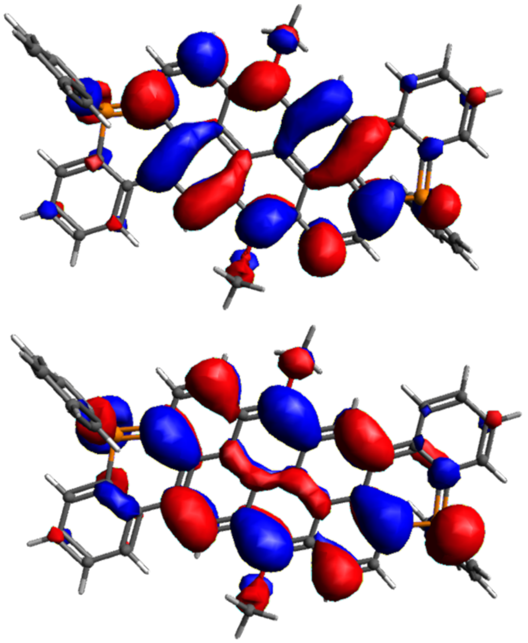

***cis*-1**

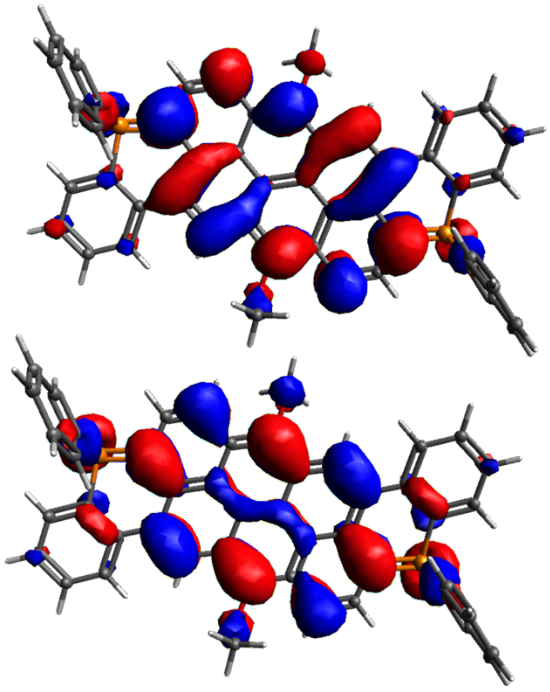

***trans*-1-Me**

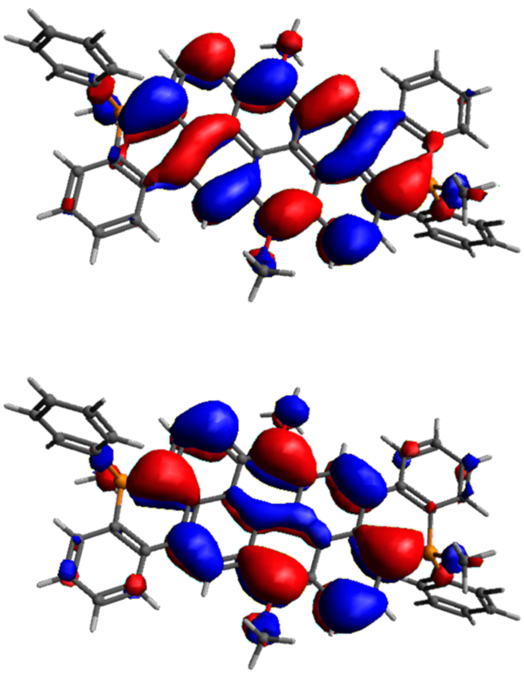

***cis*-1-Me**

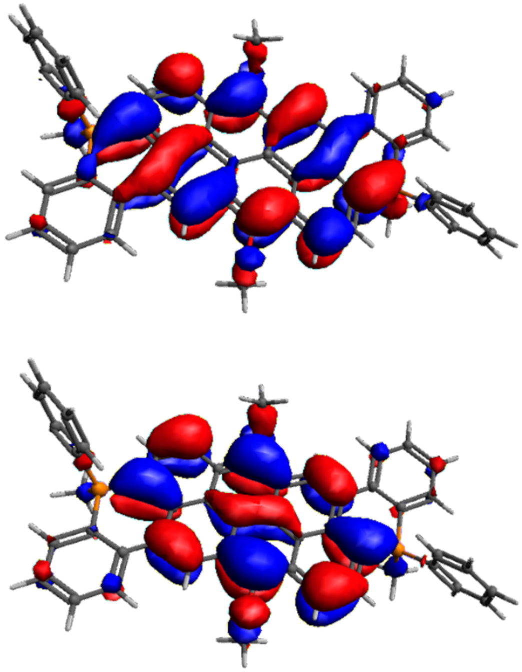

***trans*-2-O**

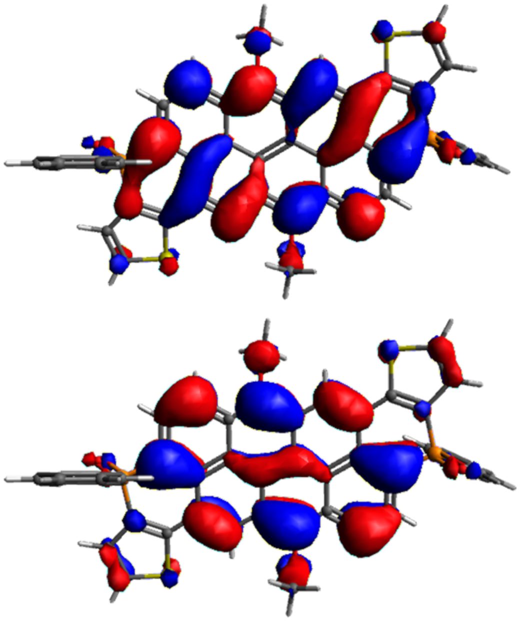

***cis*-2-O**

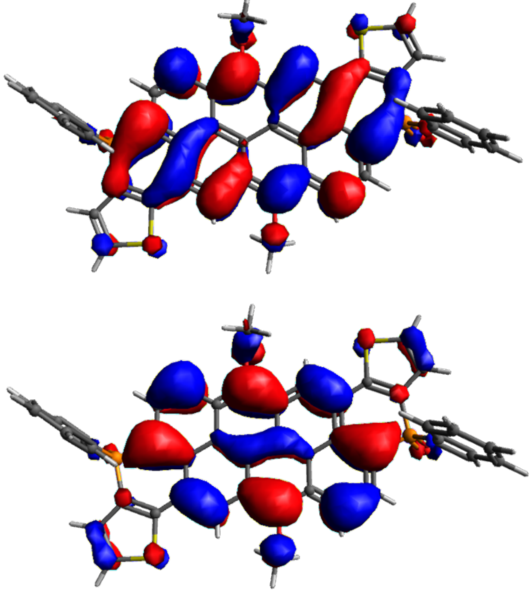

***trans*-2**

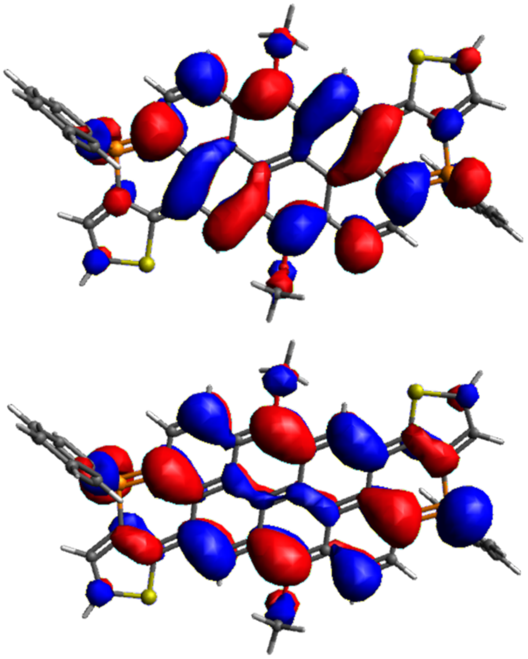

***cis*-2**

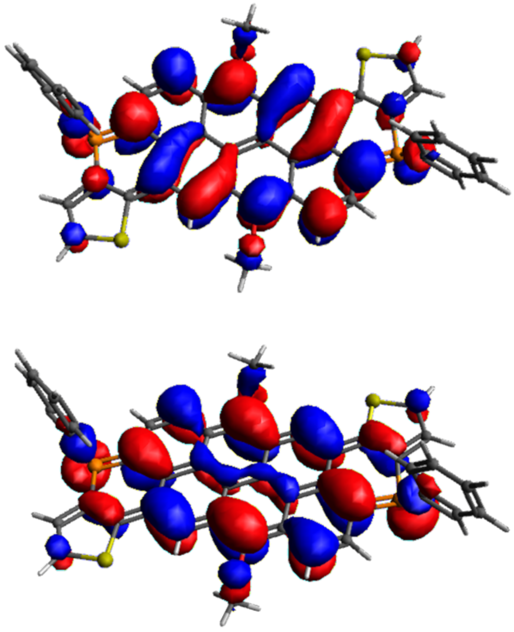

***trans*-2-Me**

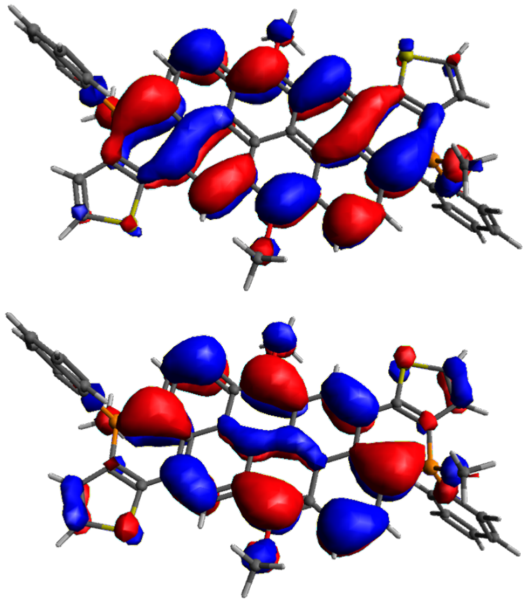

***cis*-2-Me**

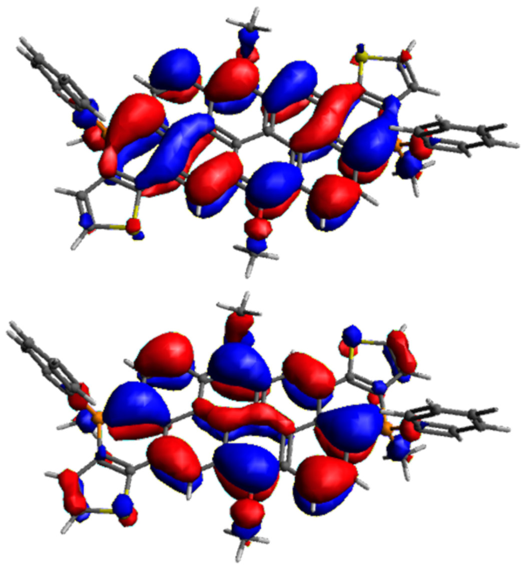

***cis*-2-O(BCF)$_2$**

LUMO (side view)

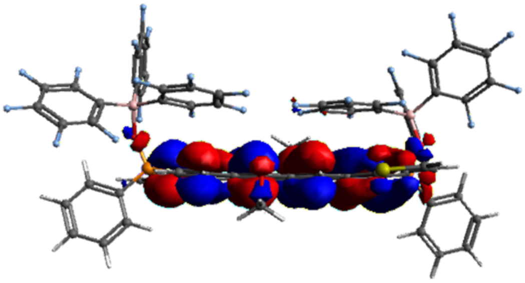

LUMO (top view)

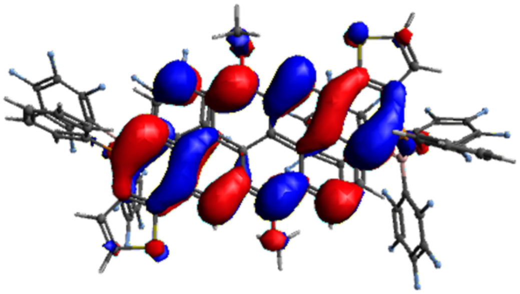

HOMO (side view)

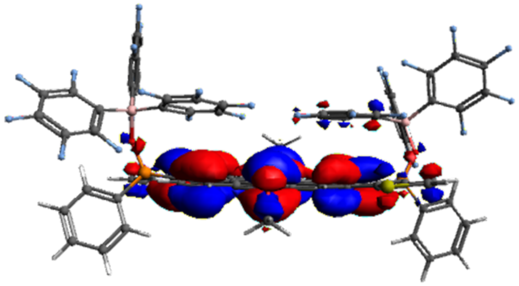

HOMO (top view)

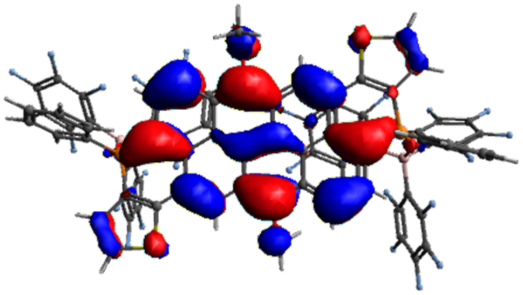

4.3. Computational Investigation of the Interaction of *cis*-2-O with BCF

The new compounds are notable for their distinctive UV-Vis spectra, characterized by peaks at approximately 375 and 510 nm. The P=O groups on these compounds function as exocyclic Lewis bases. When the compounds interact with the Lewis acid tris(pentafluorophenyl)borane (BCF) (Figure S5), a red shift of both peaks is observed. Moreover, the peak at 510 nm experiences a more pronounced shift than the peak at 375 nm (Figure S6). We performed TD-DFT calculations to provide a deeper understanding for these BCF-induced red shifts.

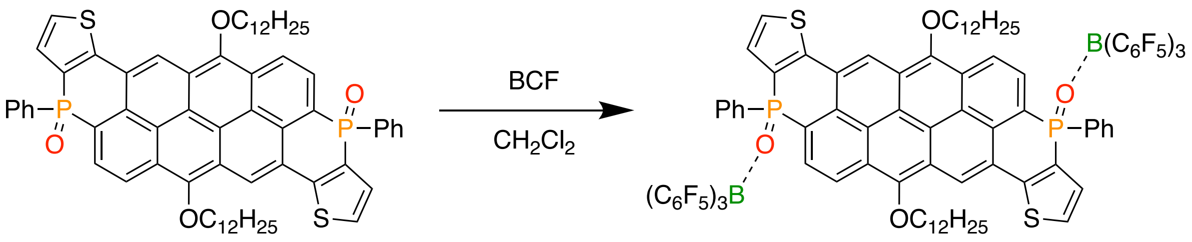

Figure S5. The coordination of $B(C_6F_5)_3$ (BCF) to ***cis*-2-O** to form ***cis*-2-O(BCF)$_2$**.

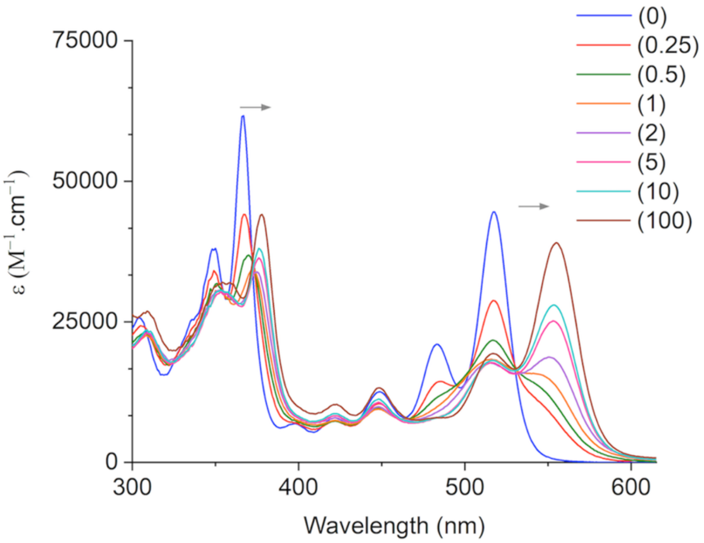

Figure S6. Experimental absorption spectra of ***cis*-2-O** in the presence of different amounts of BCF indicated by the parenthesized number. (0) means pure ***cis*-2-O** and (100) means 100 equiv. of BCF – sufficient for converting all ***cis*-2-O** to ***cis*-2-O(BCF)$_2$**.

We performed calculations for ***cis*-2-O** and ***cis*-2-O(BCF)$_2$** as representative examples to investigate the origin of the red shift upon BCF coordination. Structural optimizations were

performed at the CAM-B3LYP/D3BJ/def2-SVP level, followed by TD-DFT calculations to calculate their vertical excited states and simulate their absorption spectra (Figure S7).

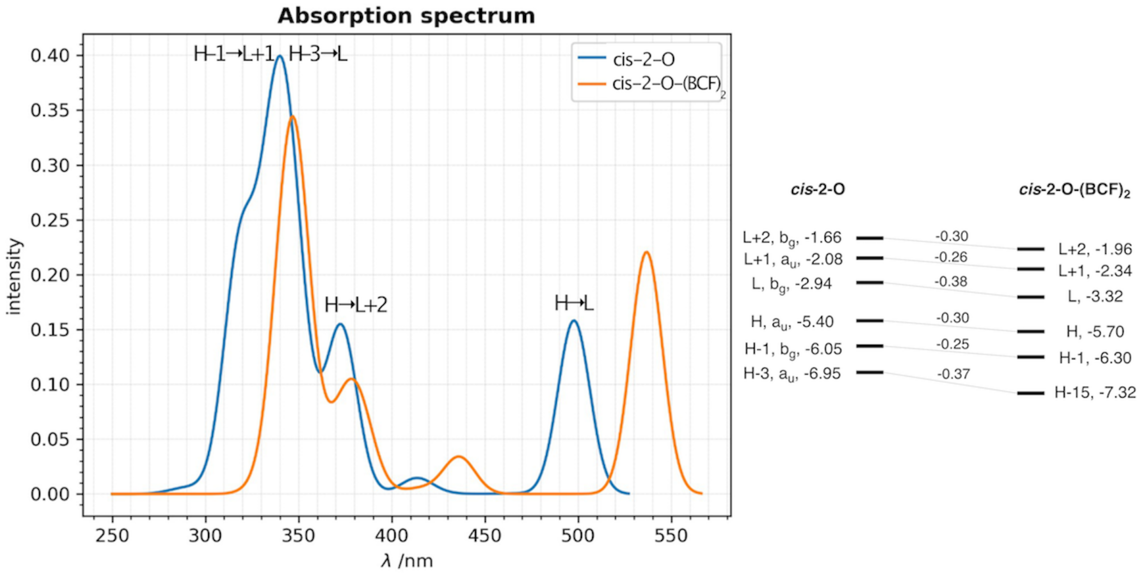

Figure S7. TD-DFT simulated absorption spectra of ***cis*-2-O** and ***cis*-2-O(BCF)₂**. Excitation schemes that are responsible for the intense absorptions are added to the ***cis*-2-O** spectrum. The correlation between molecular orbitals in ***cis*-2-O** and ***cis*-2-O(BCF)₂** are given on the right side. H, H-1, and L+1 stand for HOMO, HOMO-1, and LUMO+1, etc.

The simulated absorption spectra of ***cis*-2-O** and ***cis*-2-O*(*BCF*)*₂** are in qualitative agreement with the experimental data. Especially, the red shifts for the peaks around 500 and 350 nm upon addition of BCF are captured. Excitation schemes for the intense absorption peaks around the two wavelengths are indicated in the spectrum. In addition to the spectra, the correlations between the frontier orbitals before and after the BCF coordination that are involved in those key excitation schemes, are presented. Naturally, the coordination of the two strong Lewis acidic molecules lowers the energies of all the frontier orbitals. Among them, HOMO-3 and LUMO are stabilized more and to a similar extend (0.37 vs. 0.38 eV). Consequently, the H→L excitation is substantially red-shifted, dominating the absorption profile around 500 nm. On the contrary, the H-3→L excitation, contributing to the absorption around 350 nm, is only slightly red-shifted. HOMO-1 and LUMO+1 are stabilized to the same extent of about 0.25 eV, while HOMO and LUMO+2 are stabilized to the same extent of 0.30 eV. Consequently, the H-1→L+1 and H→L+2 excitations are also only slightly red-shifted, and these excitations also contribute to the absorptions around 350

nm. The different red shift for the absorption peaks around 500 and 350 nm are fully consistent by the lowered orbital energies induced by the BCF coordination.

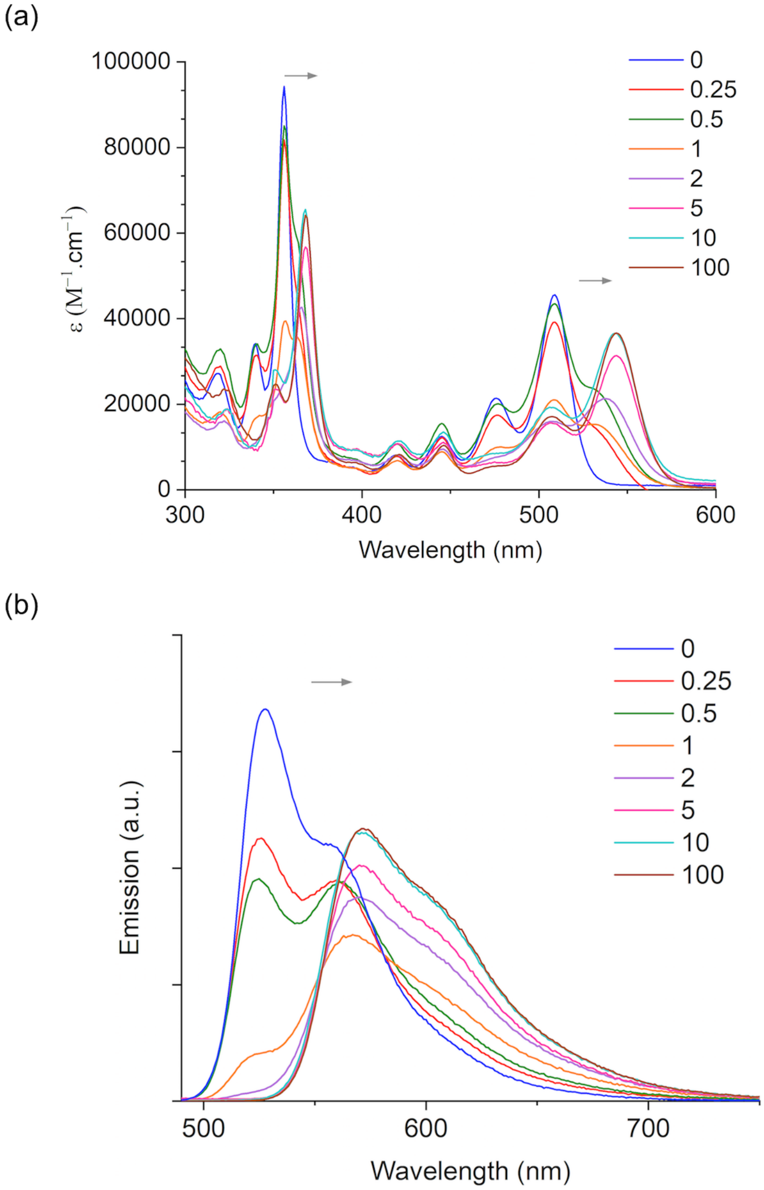

Figure S8. Absorption (a) and emission (b) spectra of ***trans*-1-O** upon titration with BCF in CH_2Cl_2 (Solutions with equal concentrations of ***trans*-1-O** with increasing amounts of BCF from 0 to 100 equivalents).

5. Steady-state spectroscopy

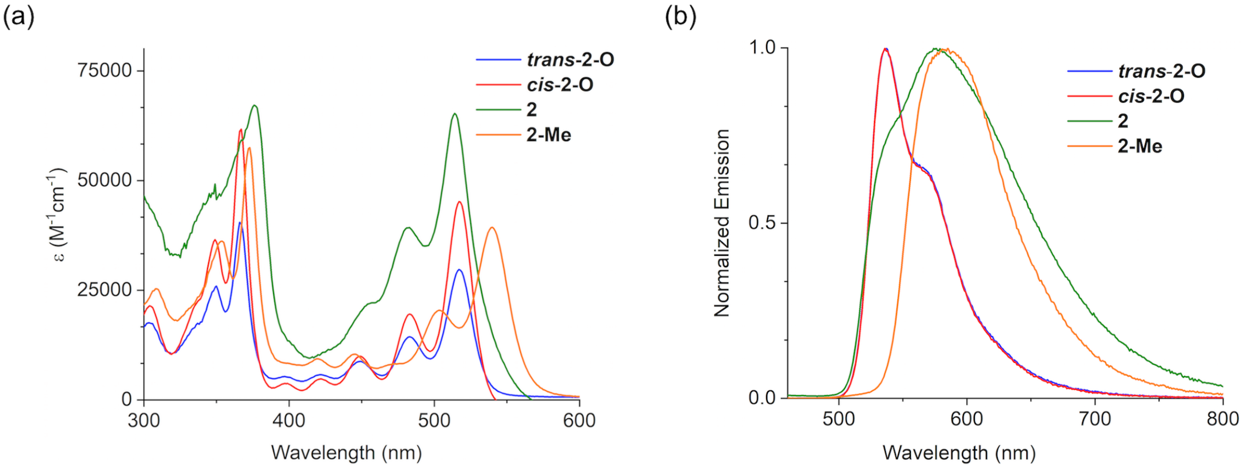

Figure S9. Absorption (a) and emission (b) spectra of thieno-fused species in CH_2Cl_2 from isomers ***trans*-2-O**, and ***cis*-2-O** and the isomeric mixtures of P-reduced **2**, and P-methylated **2-Me**.

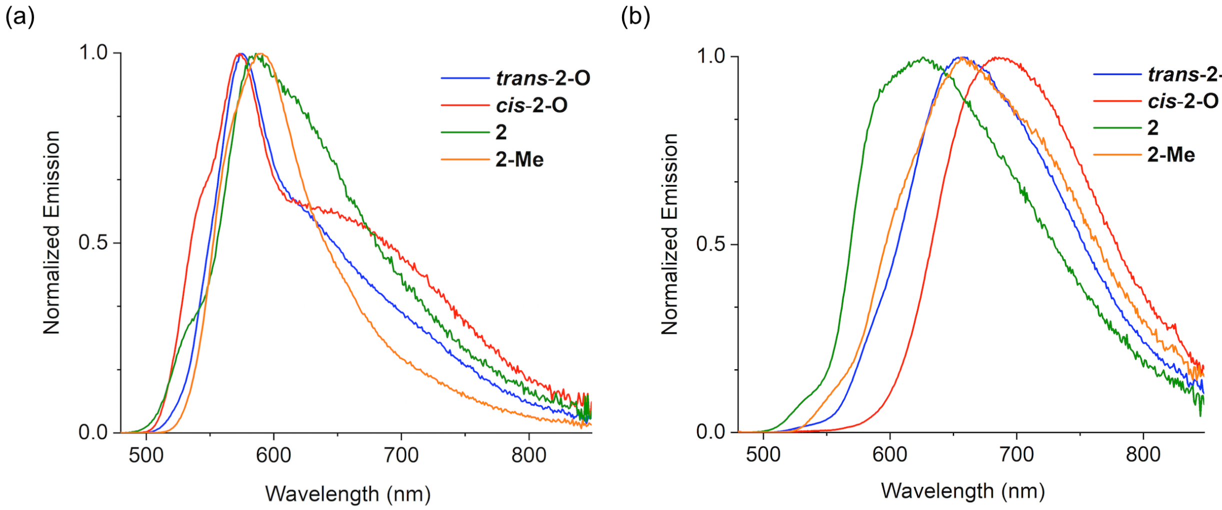

Figure S10. Emission spectra of (a) 0.2 wt%, and (b) 1.2 wt% PMMA films of thieno-fused species; separated isomers ***trans*-2-O**, and ***cis*-2-O**, along with isomeric mixtures of P-reduced **2**, and P-methylated **2-Me**.

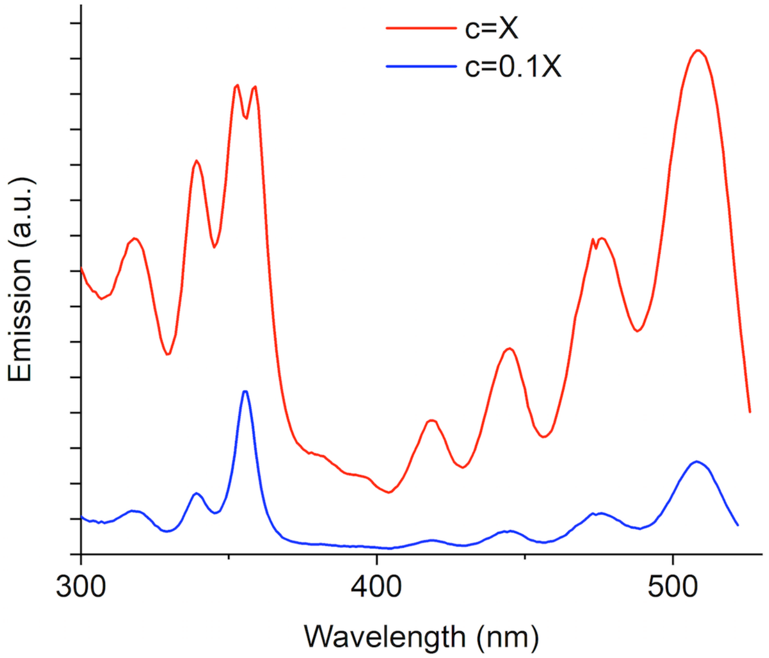

Figure S11. Excitation spectra of ***trans*-1-O** in different concentrations (X = 6.4 × 10^{-6} M).

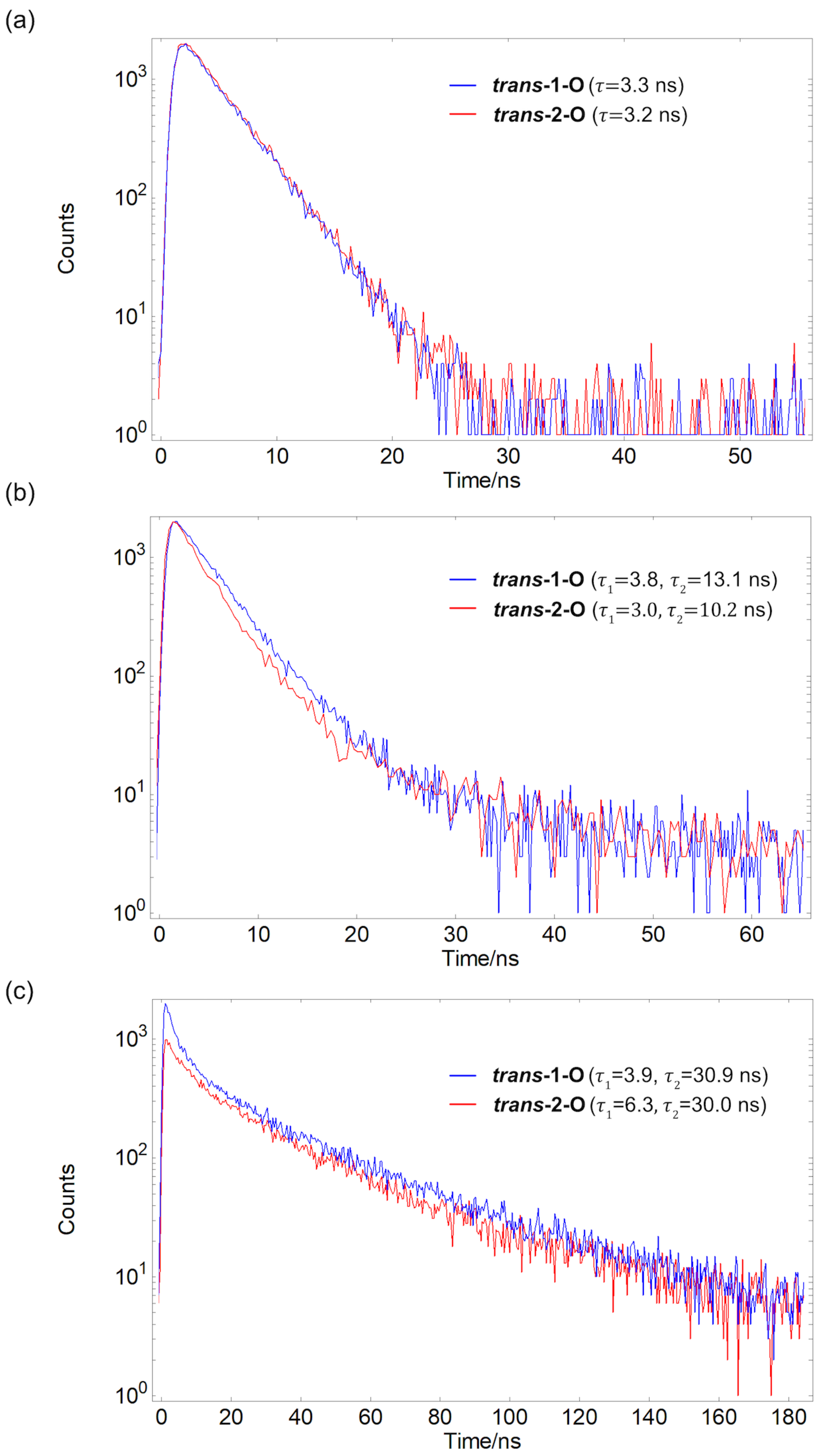

Figure S12. Excited-state lifetimes of ***trans*-1-O** and ***trans*-2-O** from a) DCM solutions, b) 0.2 wt% films in PMMA and c) 1.2 wt % films in PMMA.

6. Electrochemical characterization

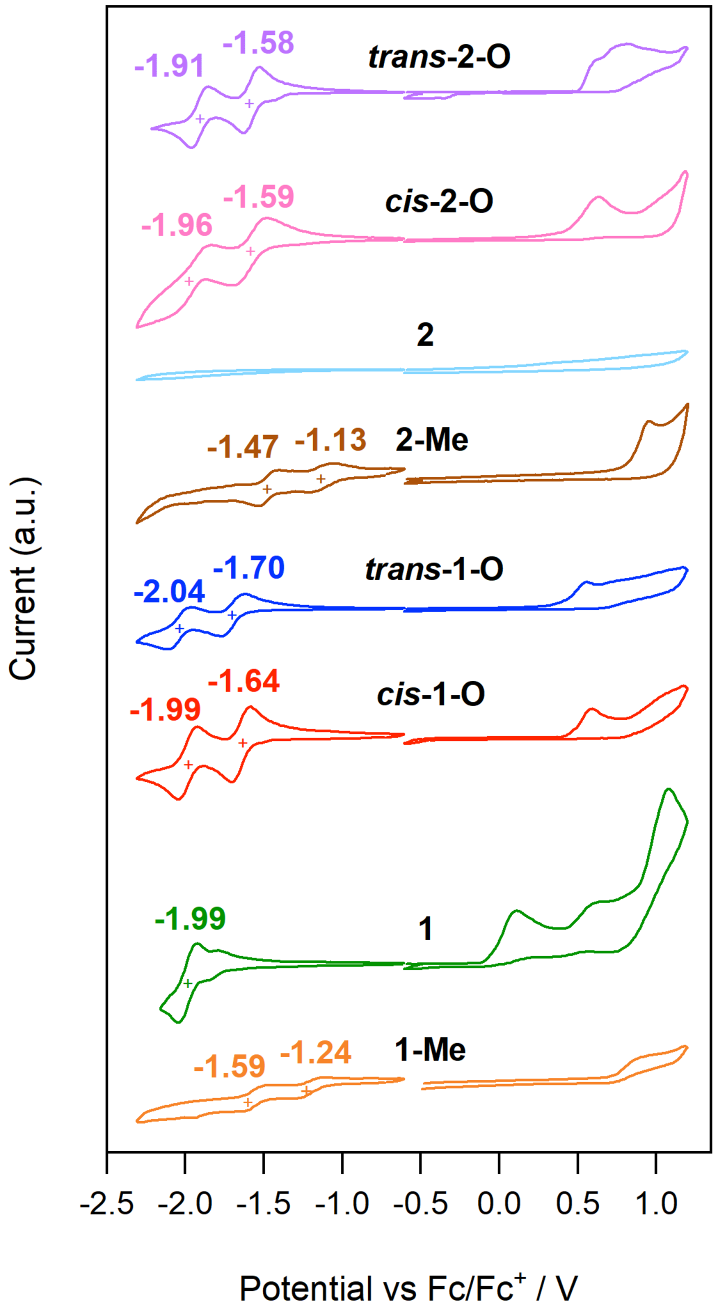

Figure S13. Cyclic voltammograms of ***trans*-2-O**, ***cis*-2-O**, **2**, **2-Me**, and ***trans*-1-O**, ***cis*-1-O**, **1**, **1-Me**, with scan rate 100 mV s^{-1}, in CH_2Cl_2 with 0.1 M NBu_4PF_6, vs. Fc/Fc^+.

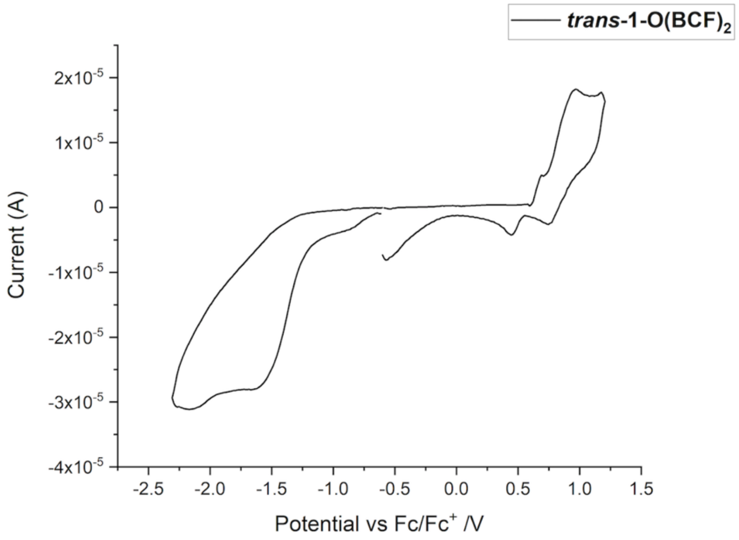

Figure S14. Cyclic voltammograms of ***trans*-1-O(BCF)$_2$**, with scan rate 100 mV s^{-1}, in CH_2Cl_2 with 0.1 M NBu_4PF_6, vs. Fc/Fc^+.

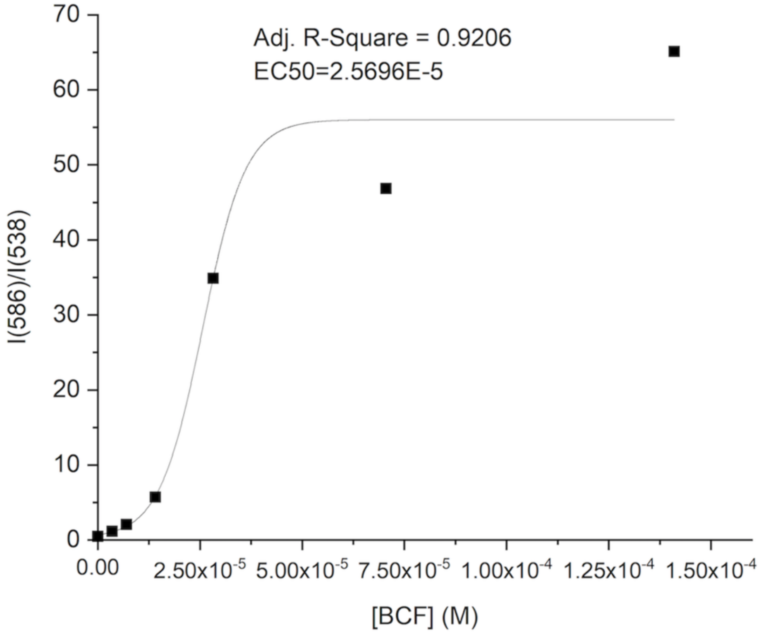

Figure S15. Sigmoidal fit of BCF titration of the ***cis*-2-O**. I(586) and I(538) is the intensity of the **cis-2-O(BCF)$_2$** and ***cis*-2-O** at their λ_{max} wavelength. The binding constant (K_{total}) for a full conversion was calculated to be **1.14×10^9 (M^{-1})** based on a method used in our previous work and developed by Lakowicz.[S11, S12]

7. NMR Spectra

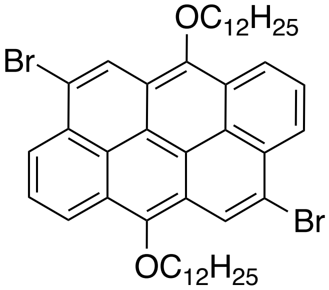

^{1}H NMR spectrum of **S1** measured in $CDCl_3$ (700 MHz).

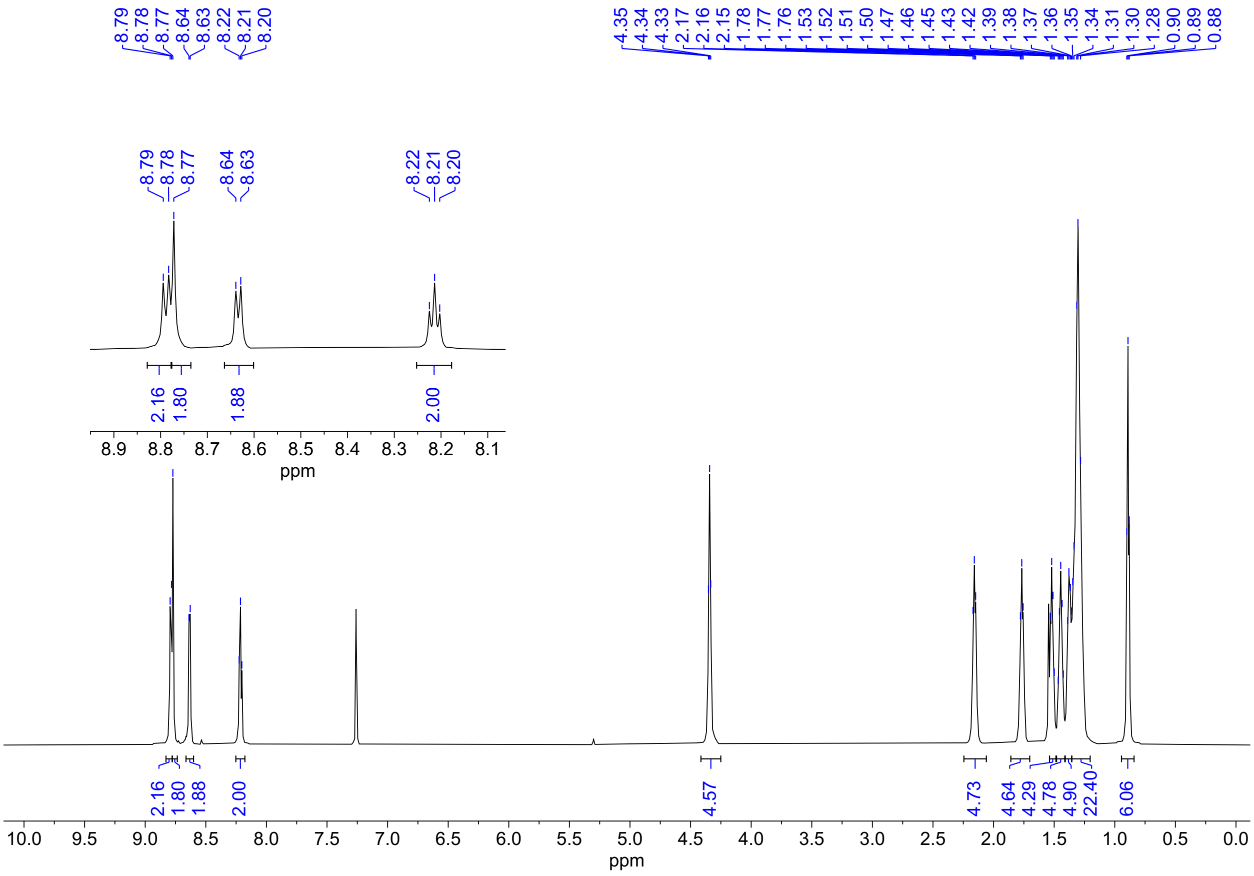

$^{13}C\{^1H\}$ NMR spectrum of **S1** measured in $CDCl_3$ (101 MHz).

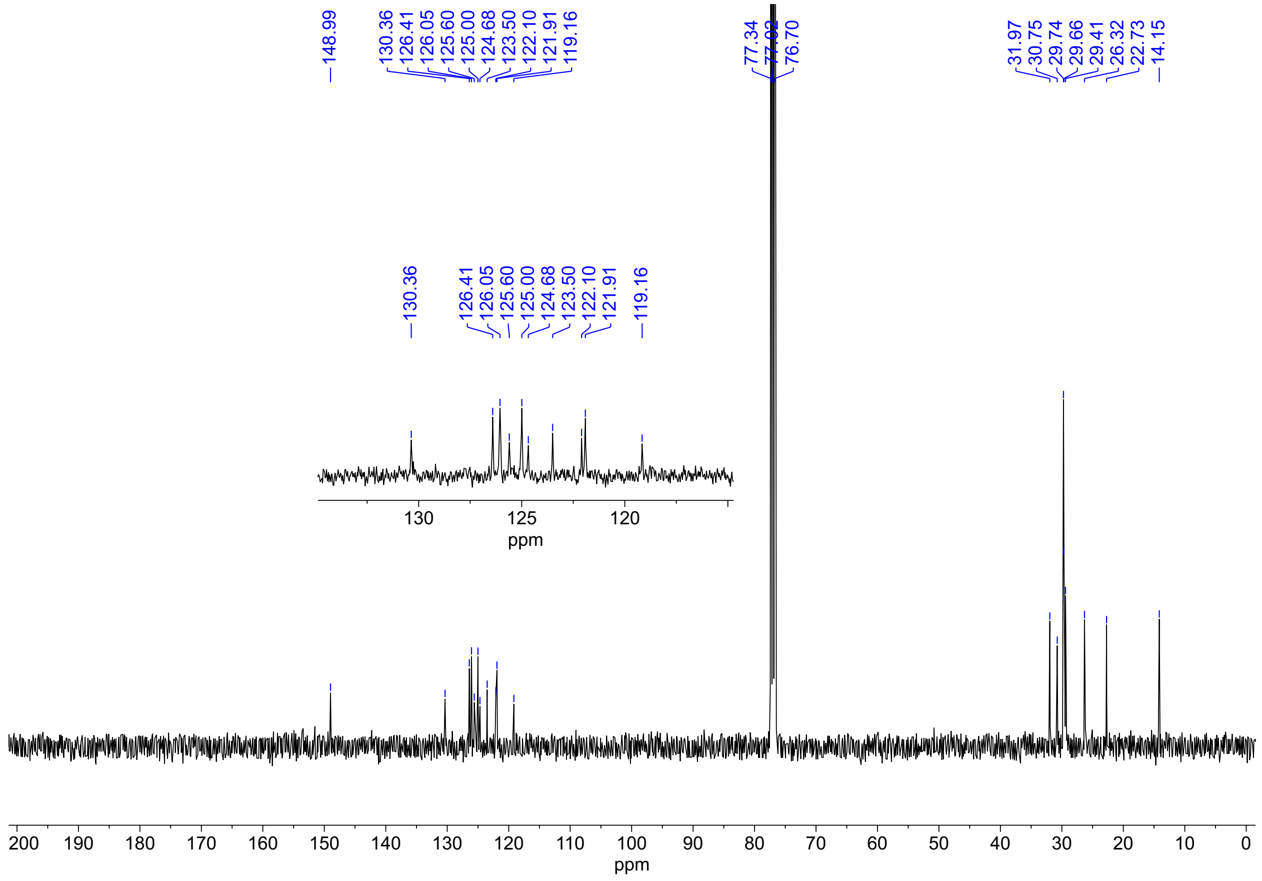

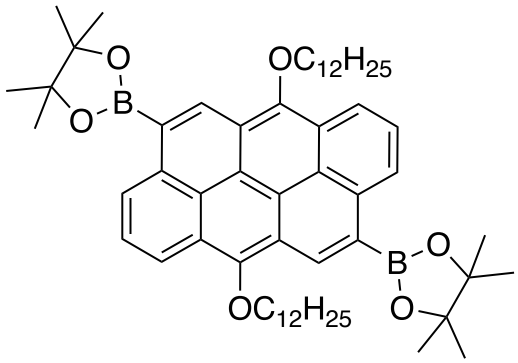

^{1}H NMR spectrum of **S2** measured in $CDCl_3$ (400 MHz).

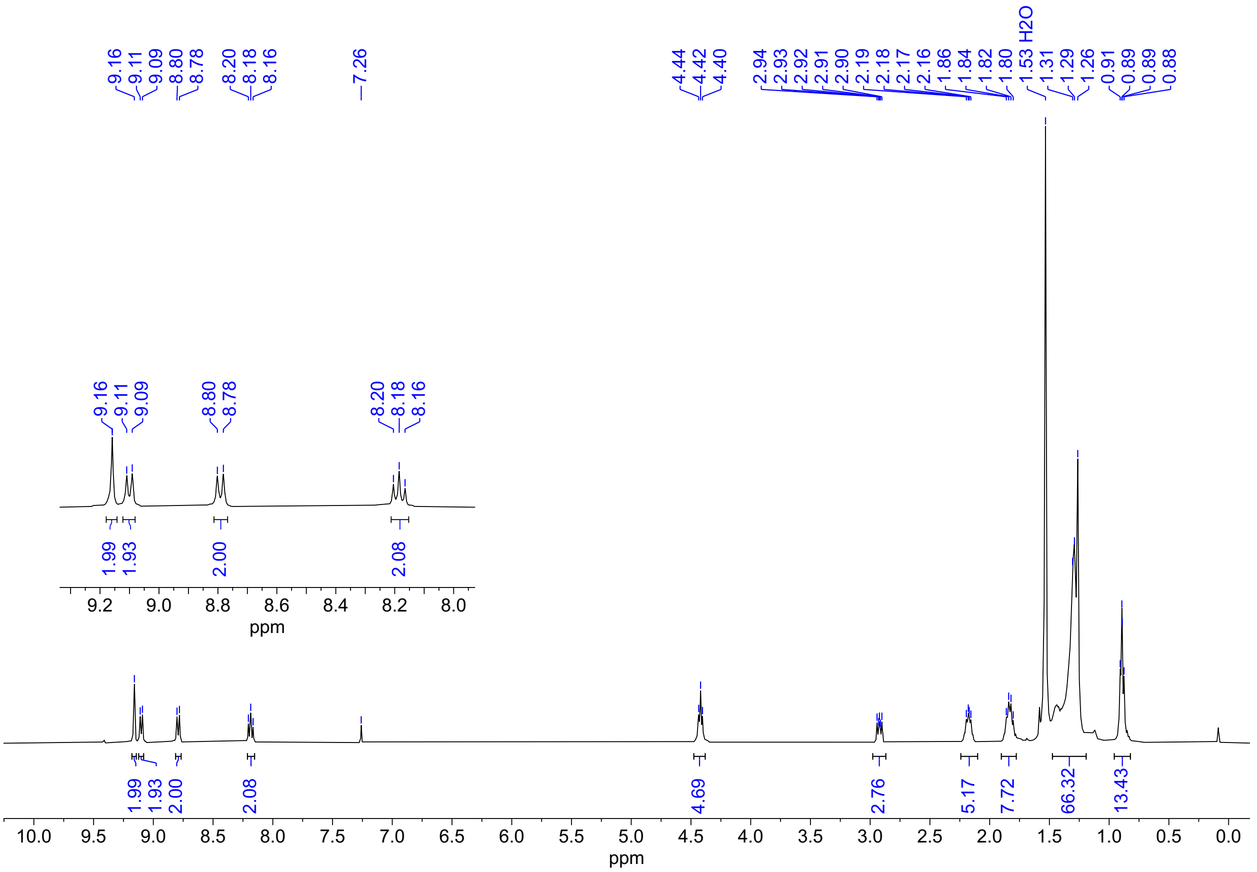

$^{13}C\{^1H\}$ NMR spectrum of **S2** measured in $CDCl_3$ (176 MHz).

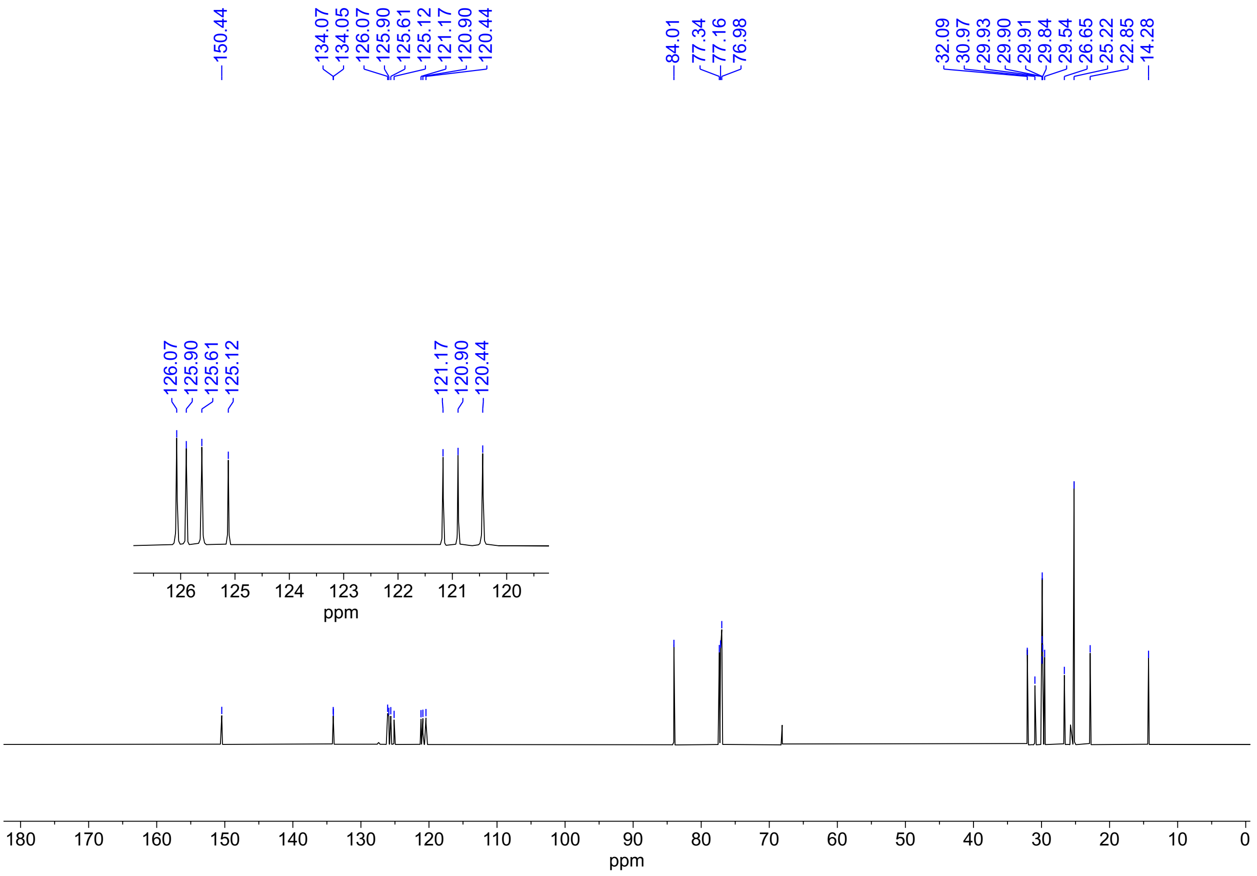

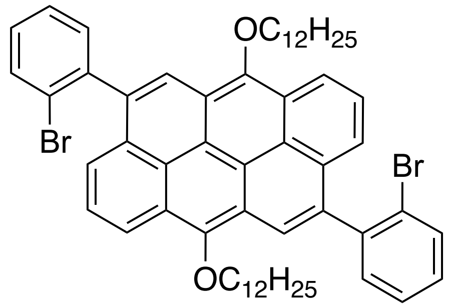

^{1}H NMR spectrum of **S3** measured in $CDCl_3$ (400 MHz).

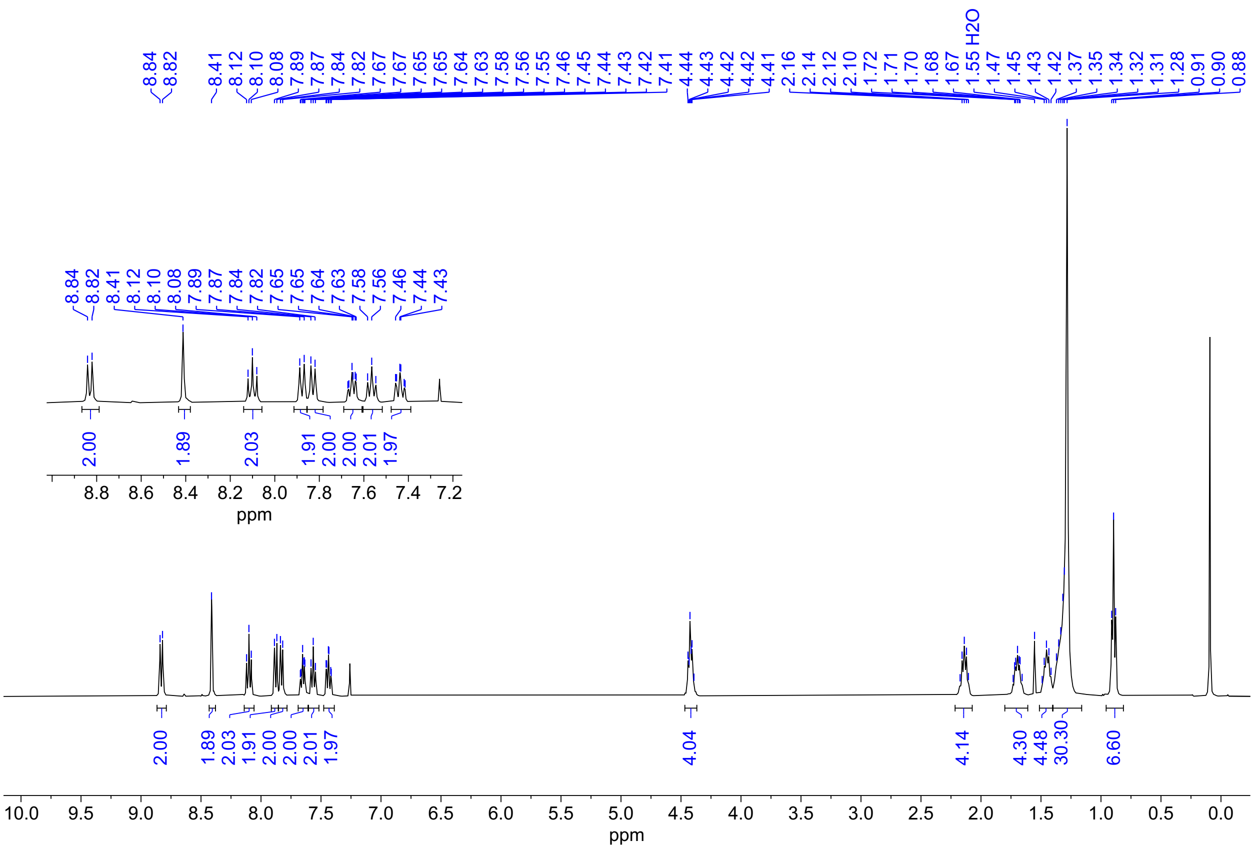

$^{13}C\{^{1}H\}$ NMR spectrum of **S3** measured in $CDCl_3$ (176 MHz).

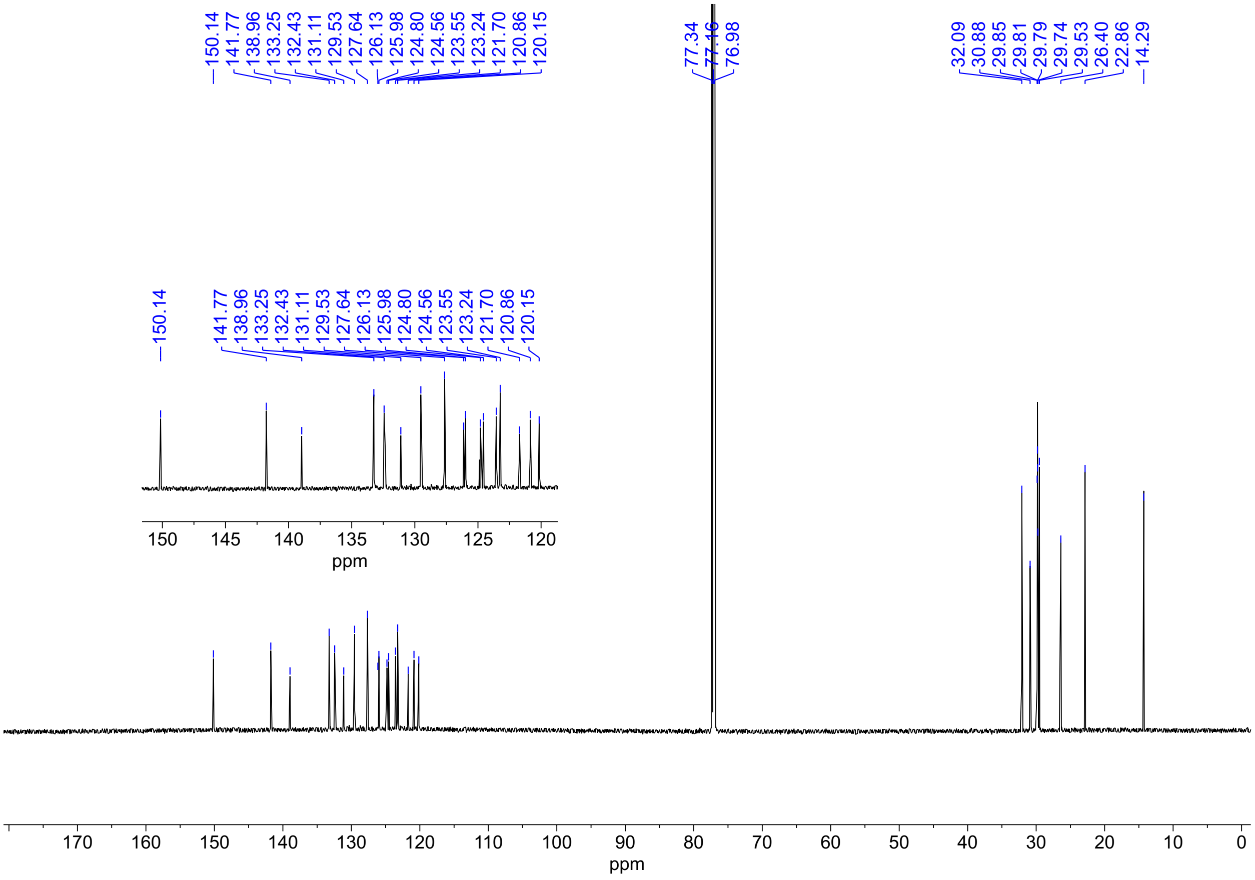

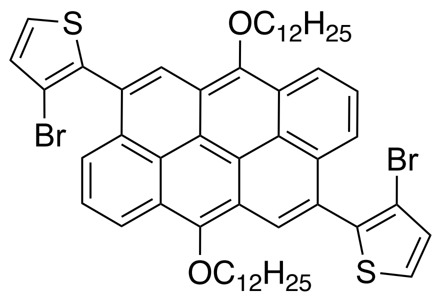

^{1}H NMR spectrum of **S4** measured in $CDCl_3$ (700 MHz).

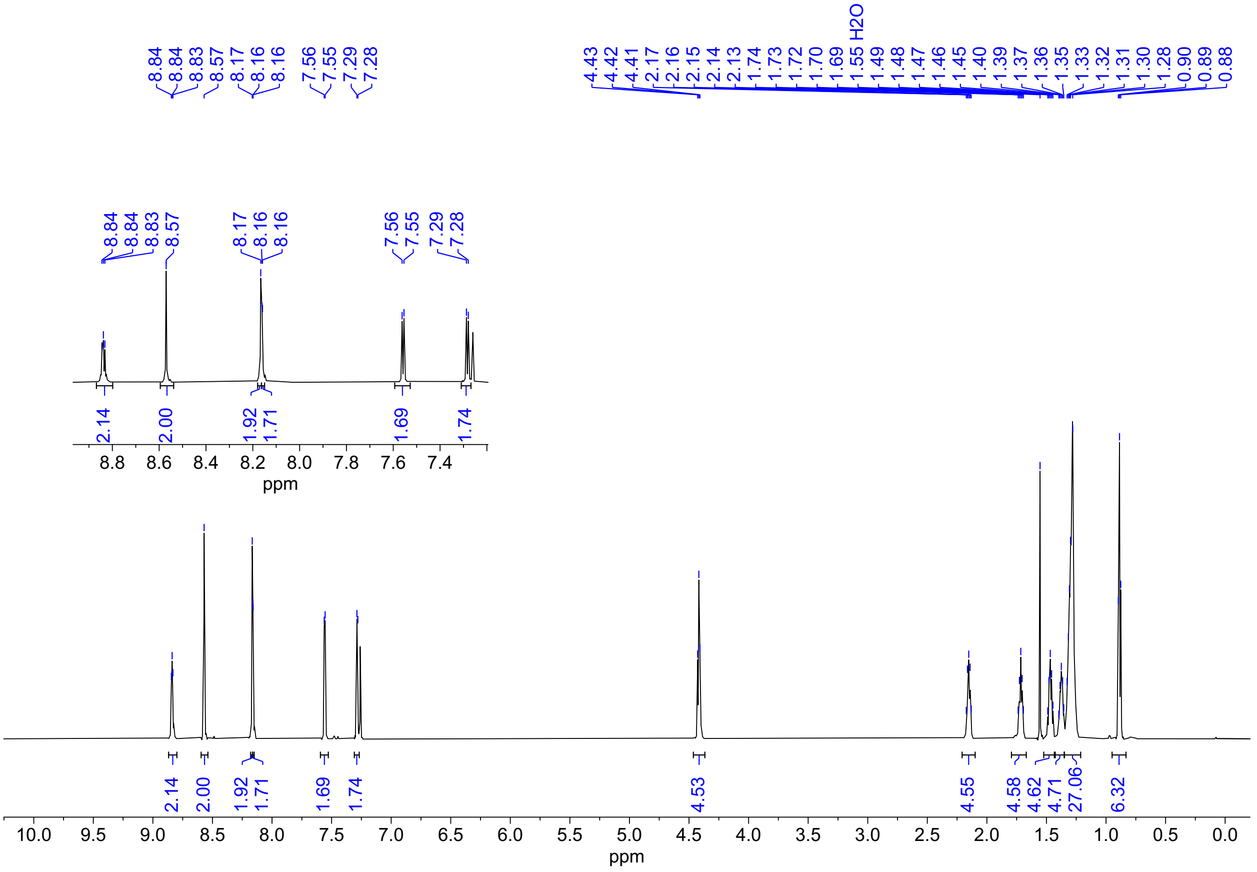

$^{13}C\{^{1}H\}$ NMR spectrum of **S4** measured in $CDCl_3$ (176 MHz).

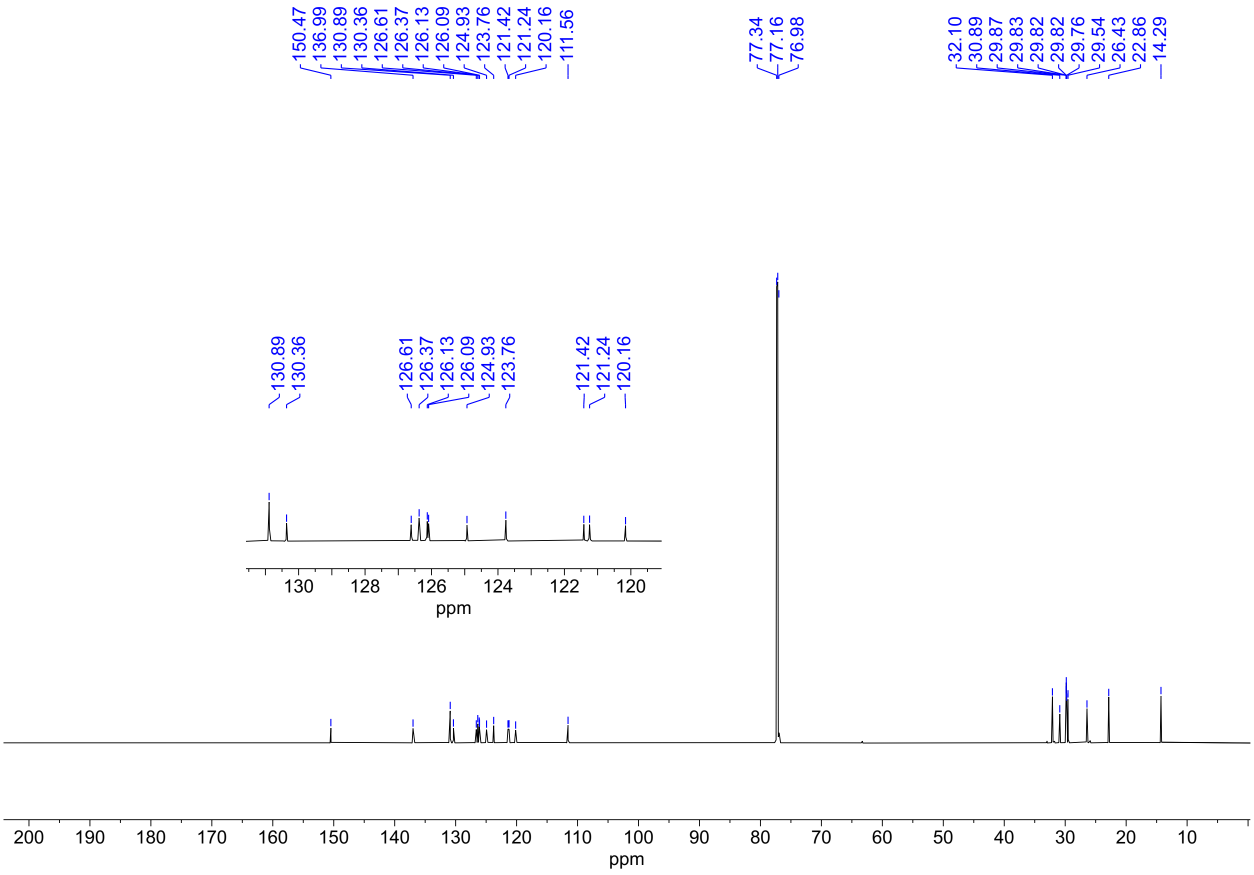

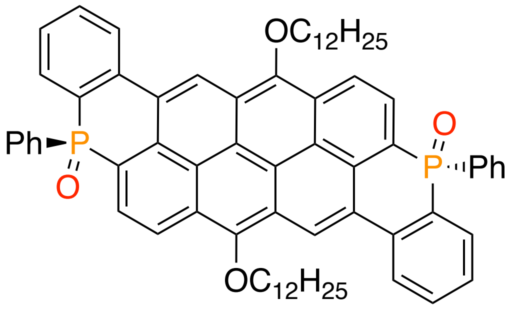

^{1}H NMR spectrum of ***trans*-1-O** measured in $CDCl_3$ (700 MHz).

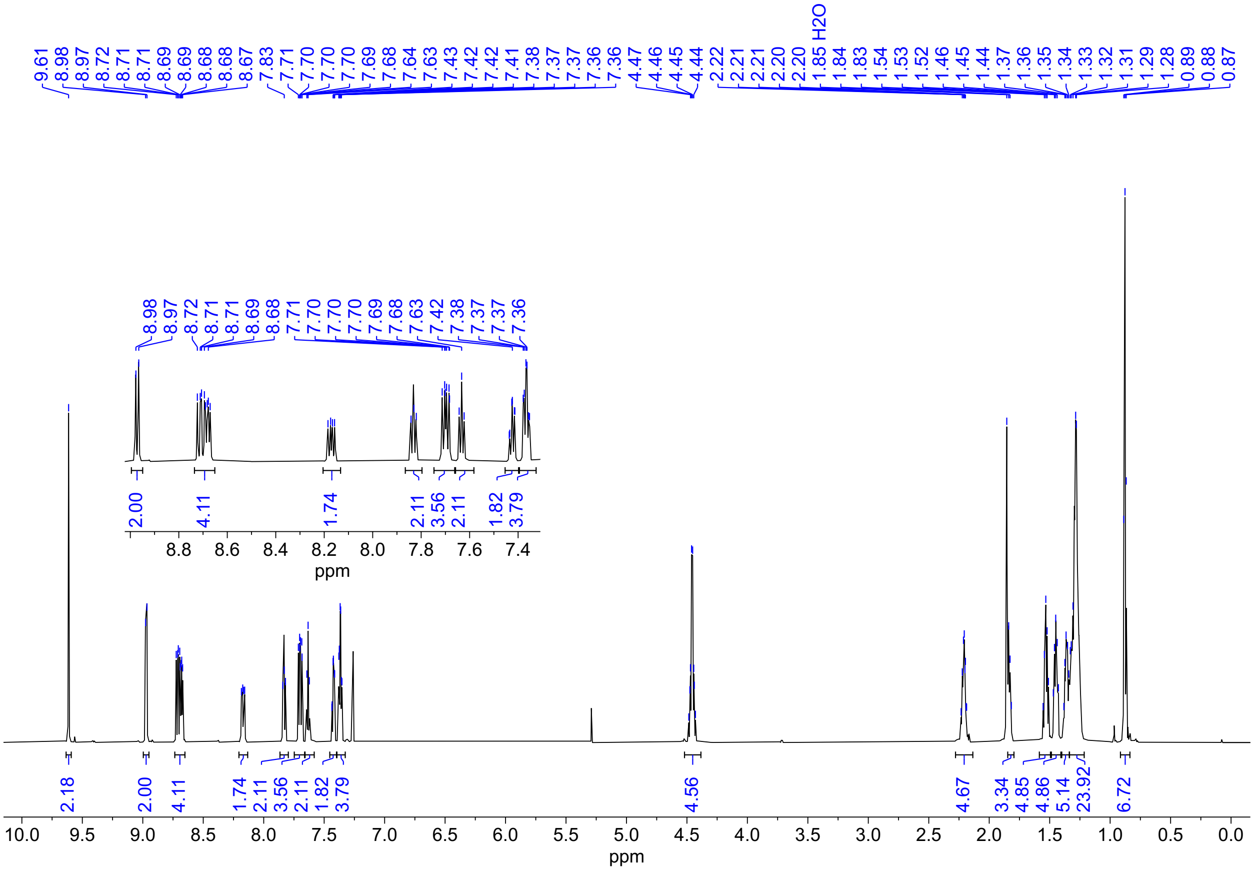

$^{13}C\{^{1}H\}$ NMR spectrum of ***trans*-1-O** measured in $CDCl_3$ (176 MHz).

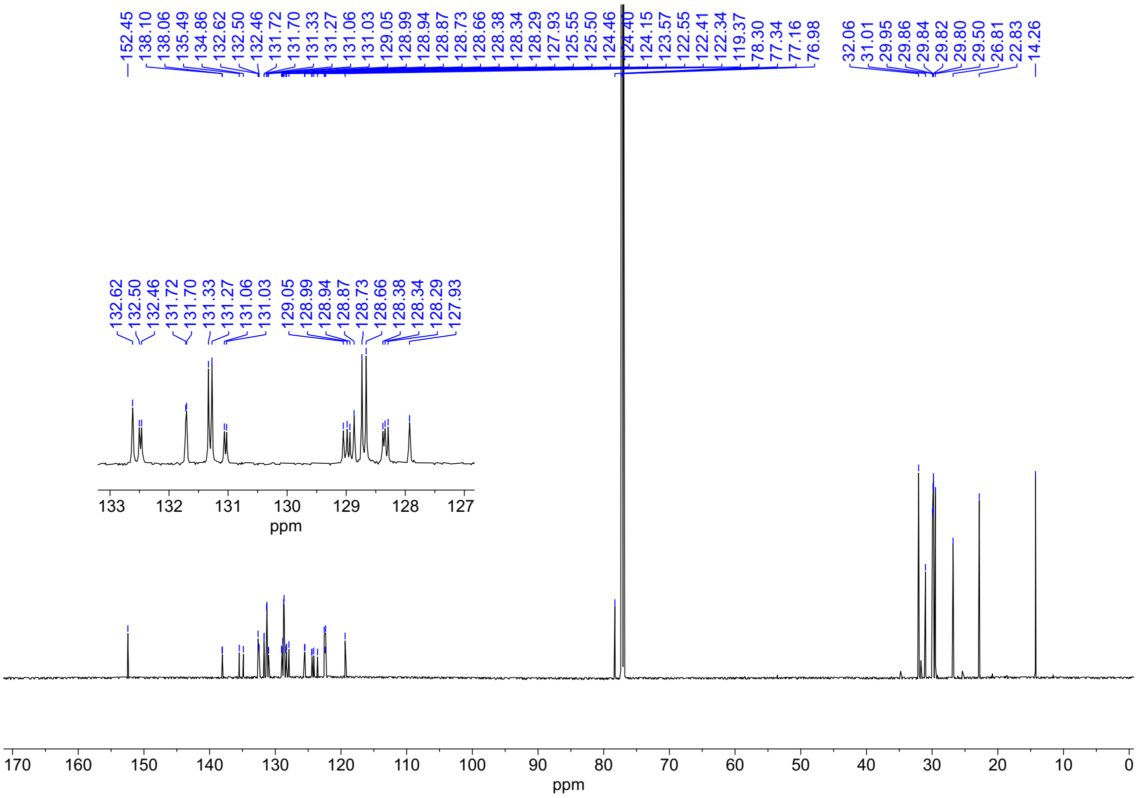

$^{31}P\{^{1}H\}$ NMR spectrum of ***trans*-1-O** measured in $CDCl_3$ (162 MHz).

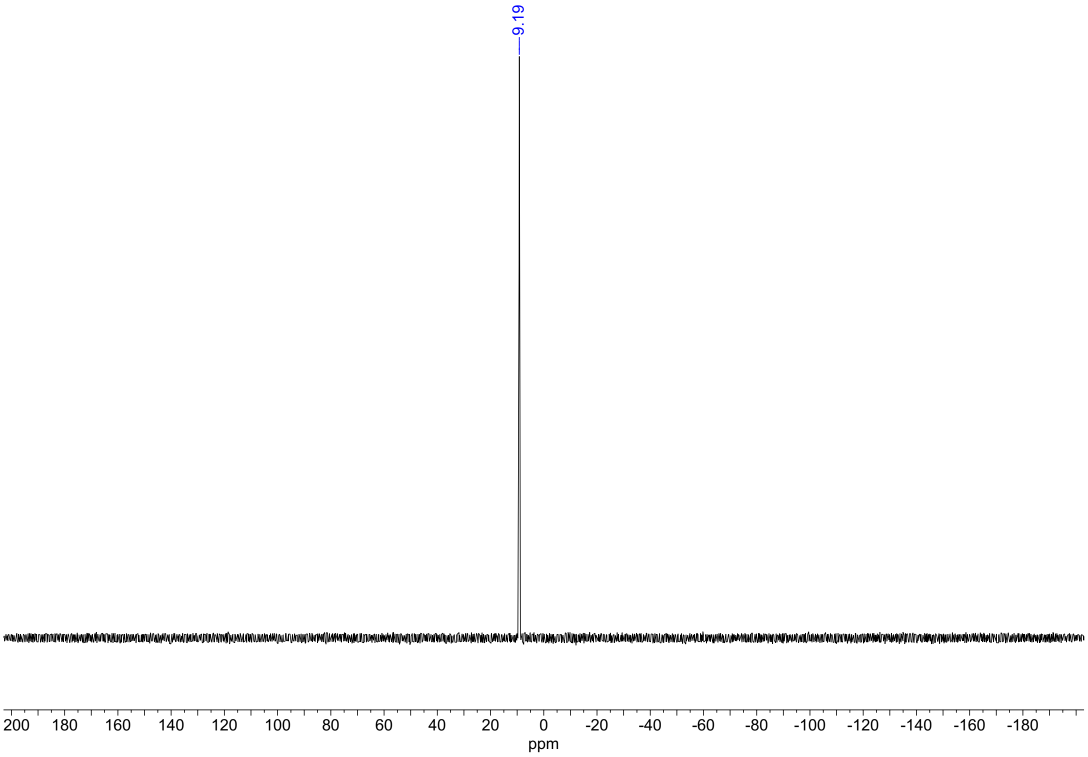

$^{13}C\{^{1}H\}$ DEPT 135 NMR spectrum of ***trans*-1-O** measured in $CDCl_3$ (176 MHz).

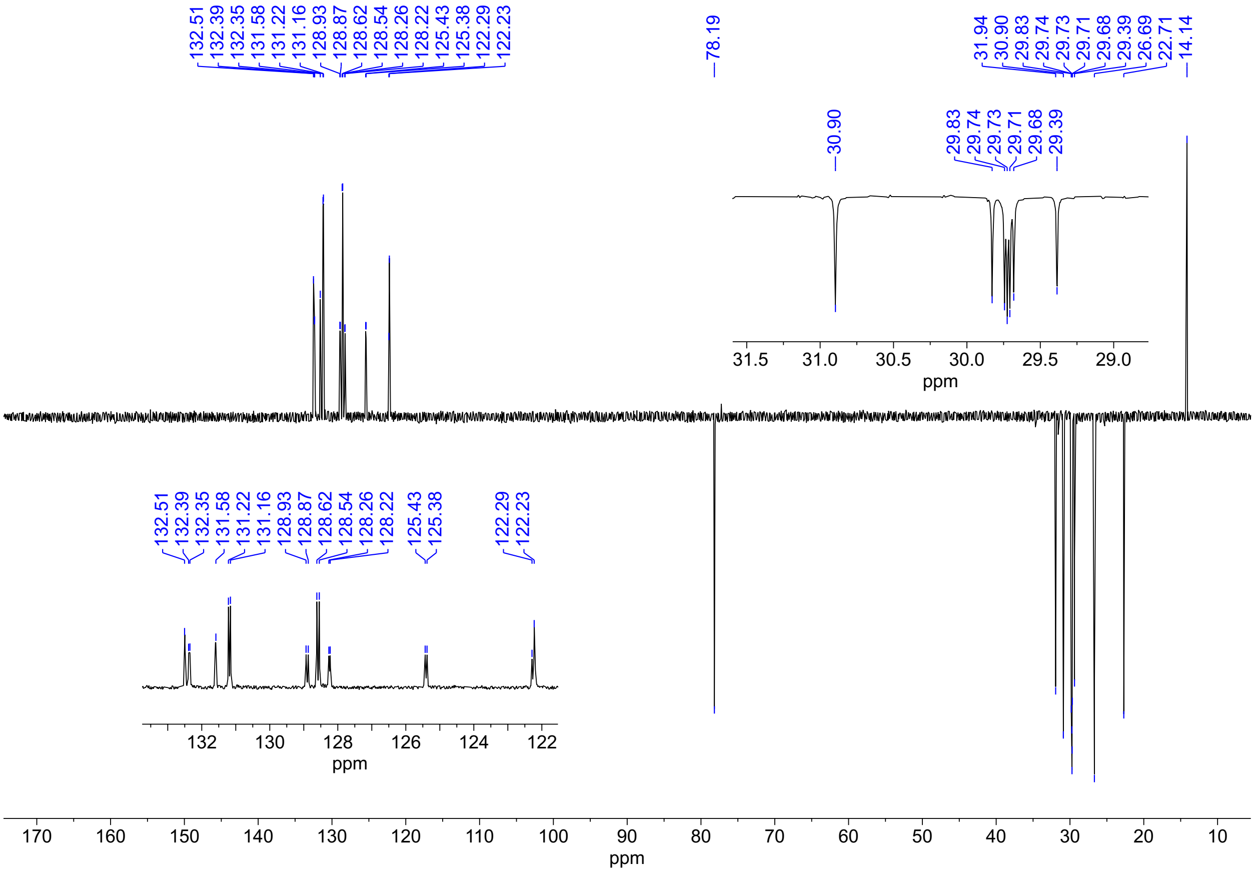

2D HSQC-DEPT 135 NMR spectrum of ***trans*-1-O** measured in $CDCl_3$ (700 MHz).

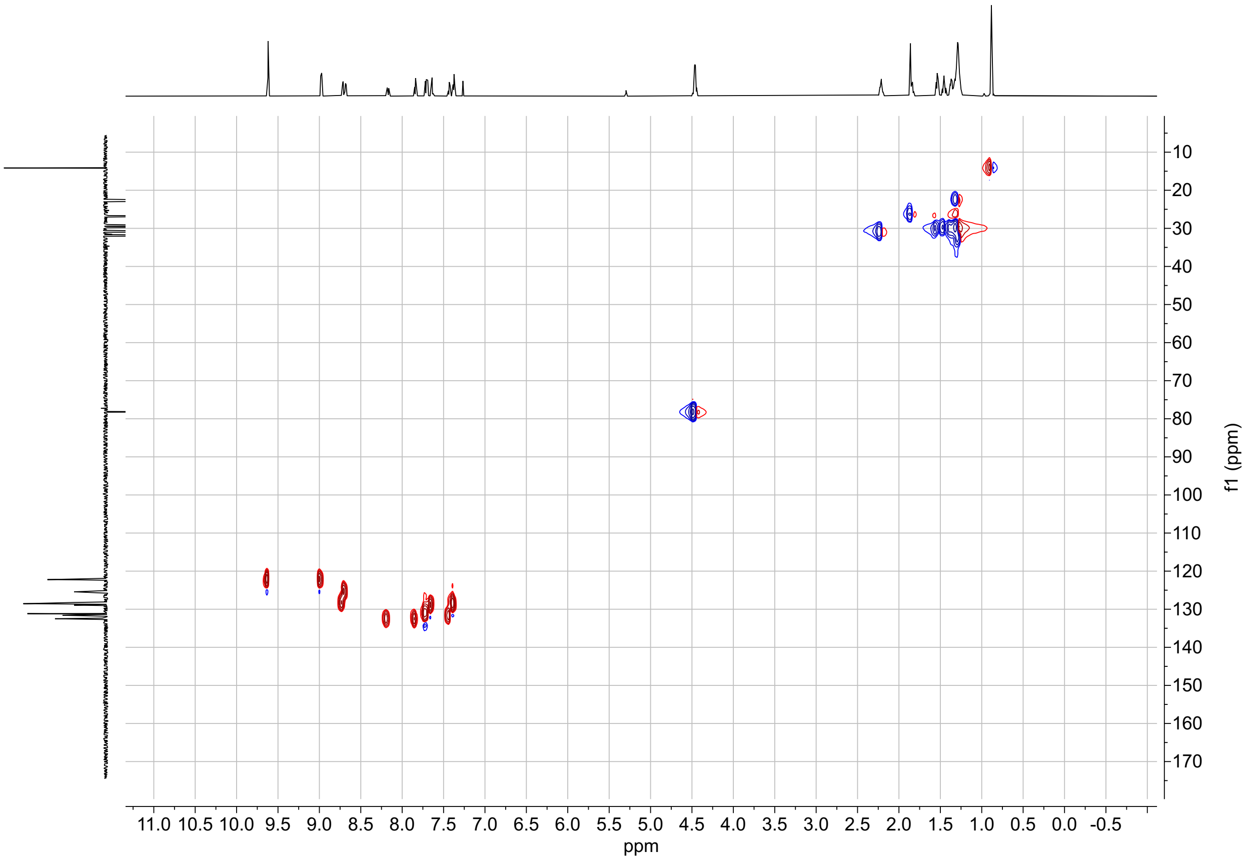

2D HMBC NMR spectrum of ***trans*-1-O** measured in $CDCl_3$ (700 MHz).

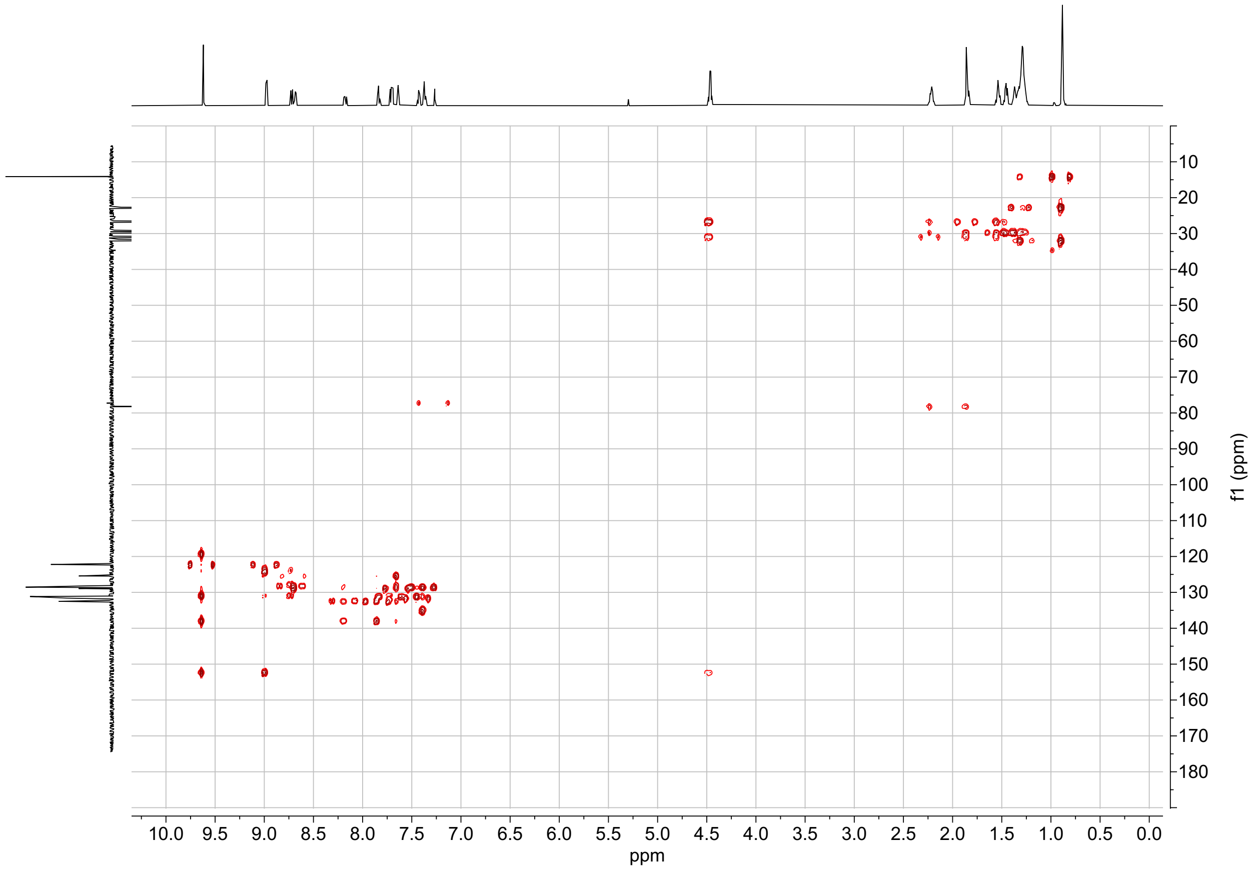

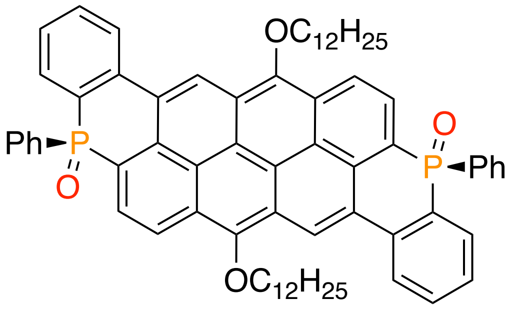

^{1}H NMR spectrum of ***cis*-1-O** measured in $CDCl_3$ (400 MHz).

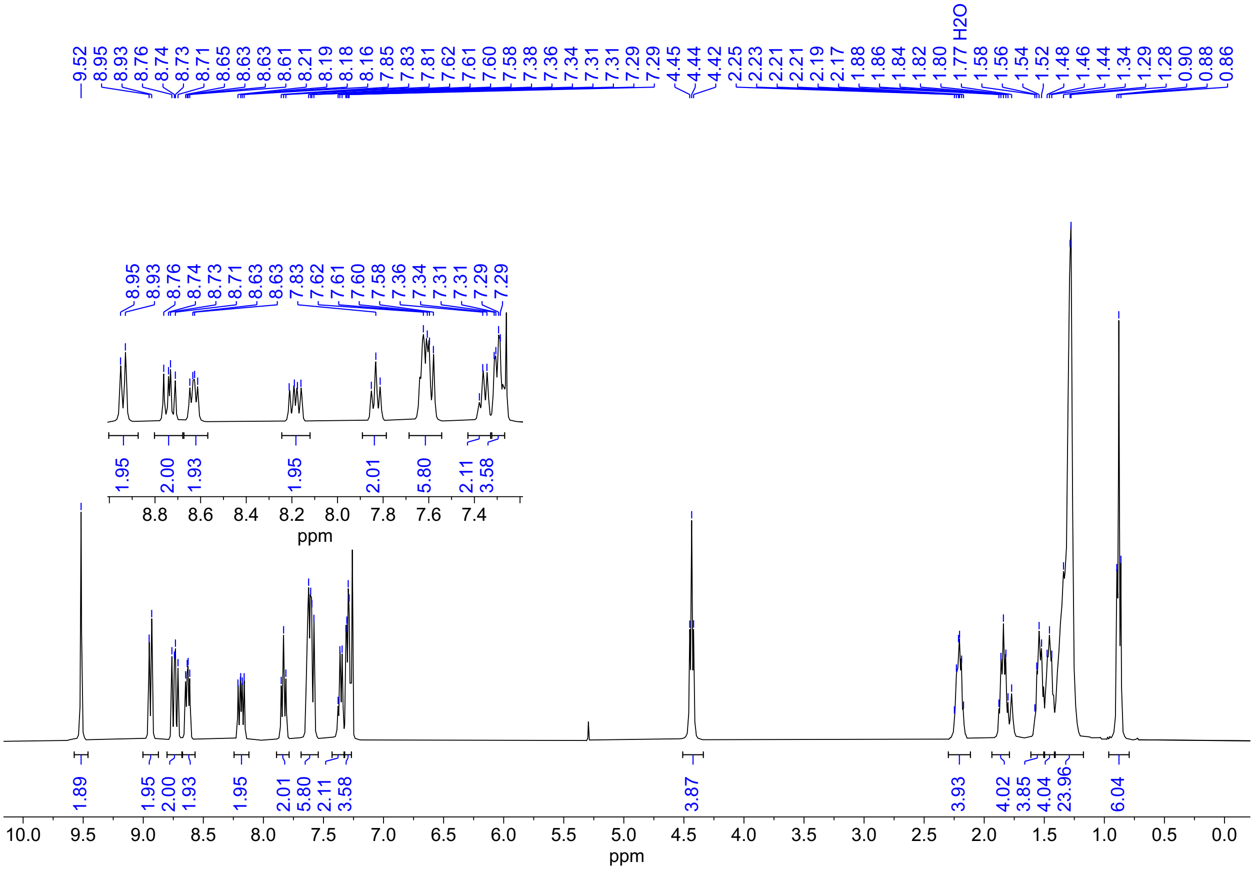

$^{13}C\{^1H\}$ NMR spectrum of ***cis*-1-O** measured in $CDCl_3$ (101 MHz).

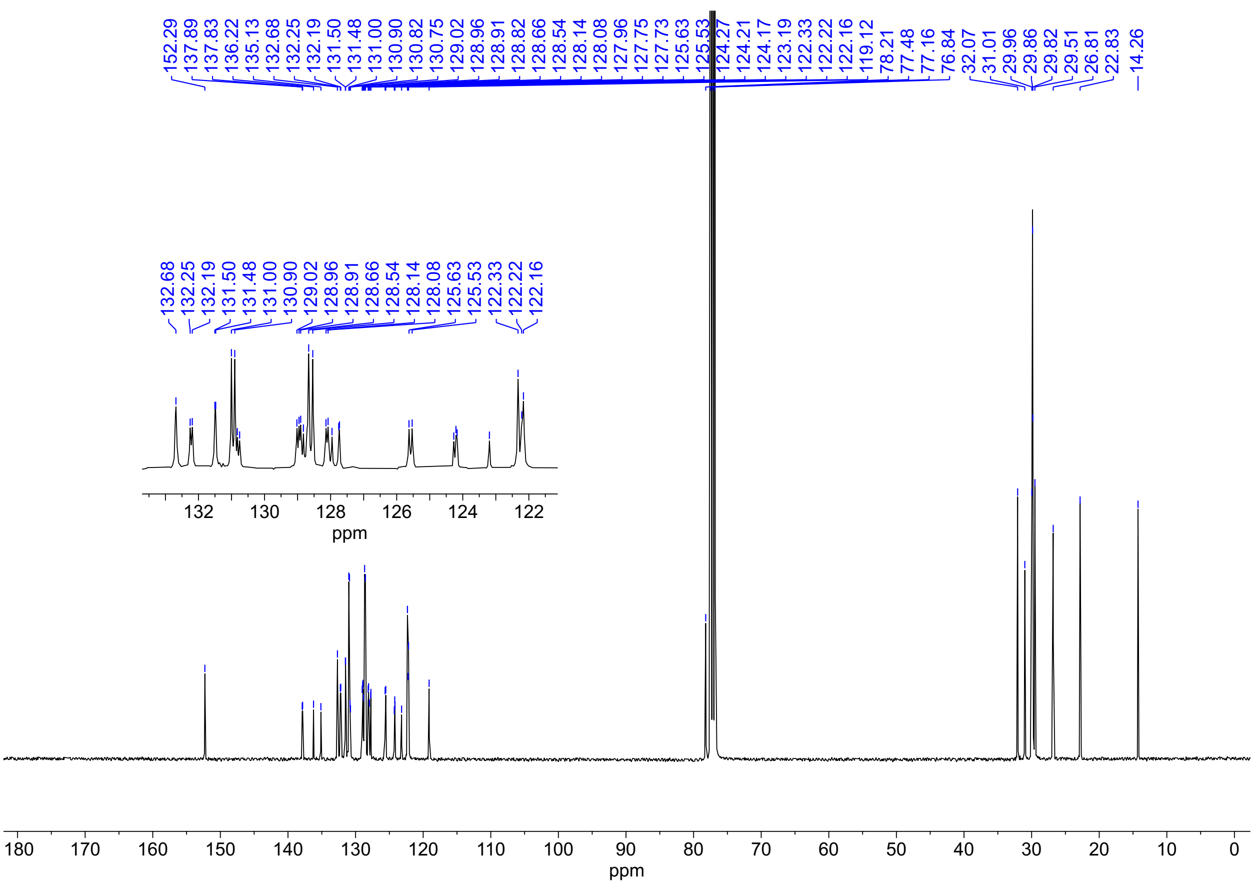

$^{31}P\{^{1}H\}$ NMR spectrum of ***cis*-1-O** measured in $CDCl_3$ (162 MHz).

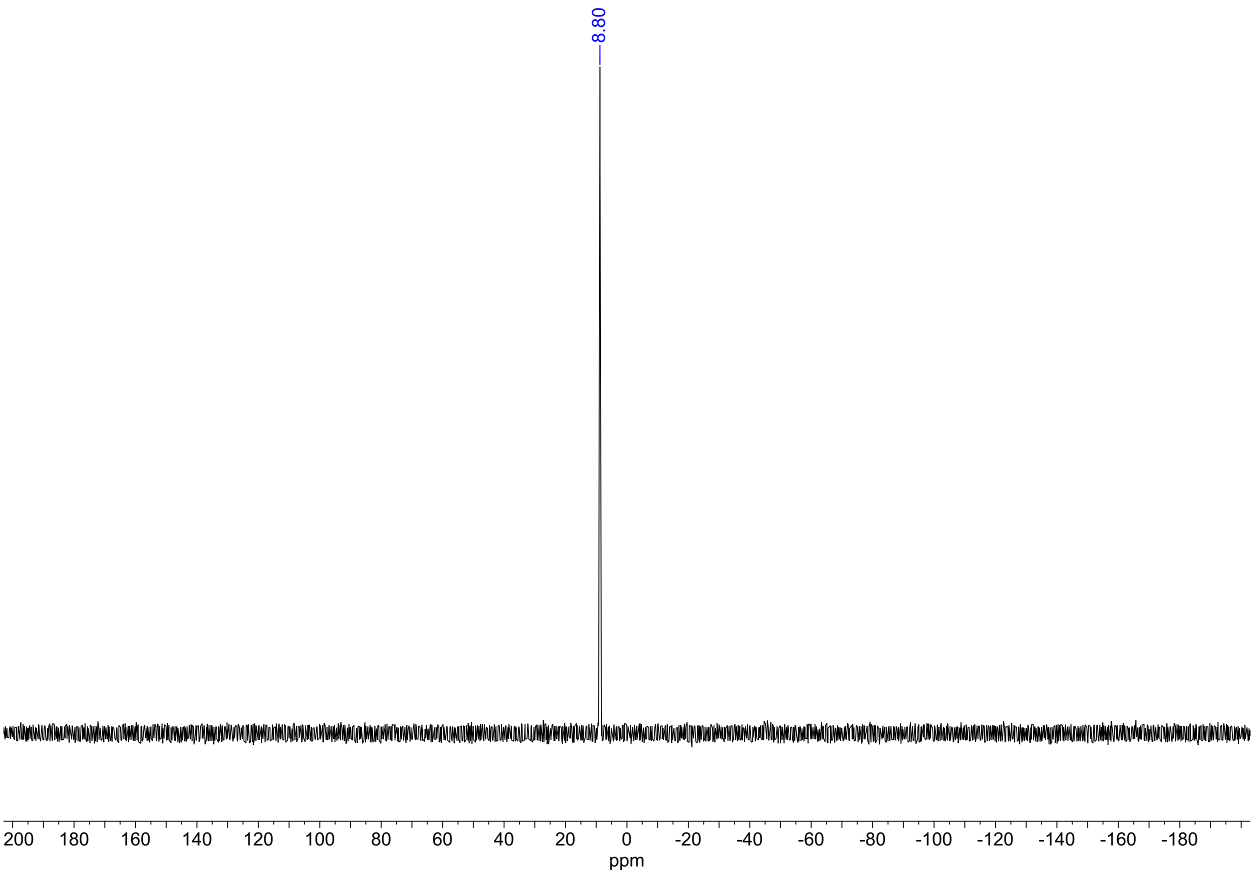

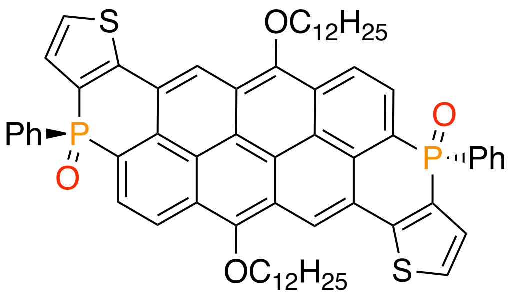

^{1}H NMR spectrum of ***trans*-2-O** measured in $CDCl_3$ (400 MHz).

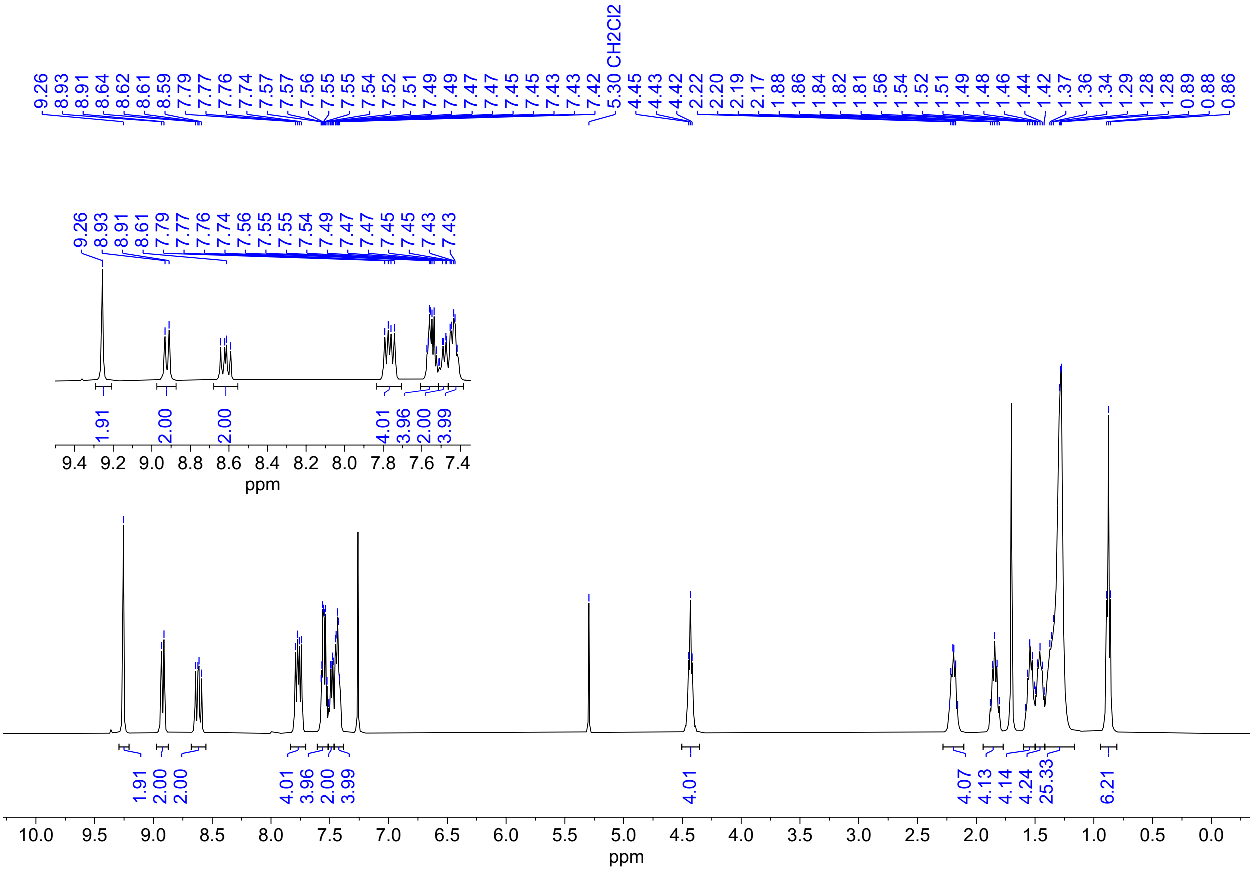

$^{13}C\{^{1}H\}$ NMR spectrum of ***trans*-2-O** measured in $CDCl_3$ (101 MHz).

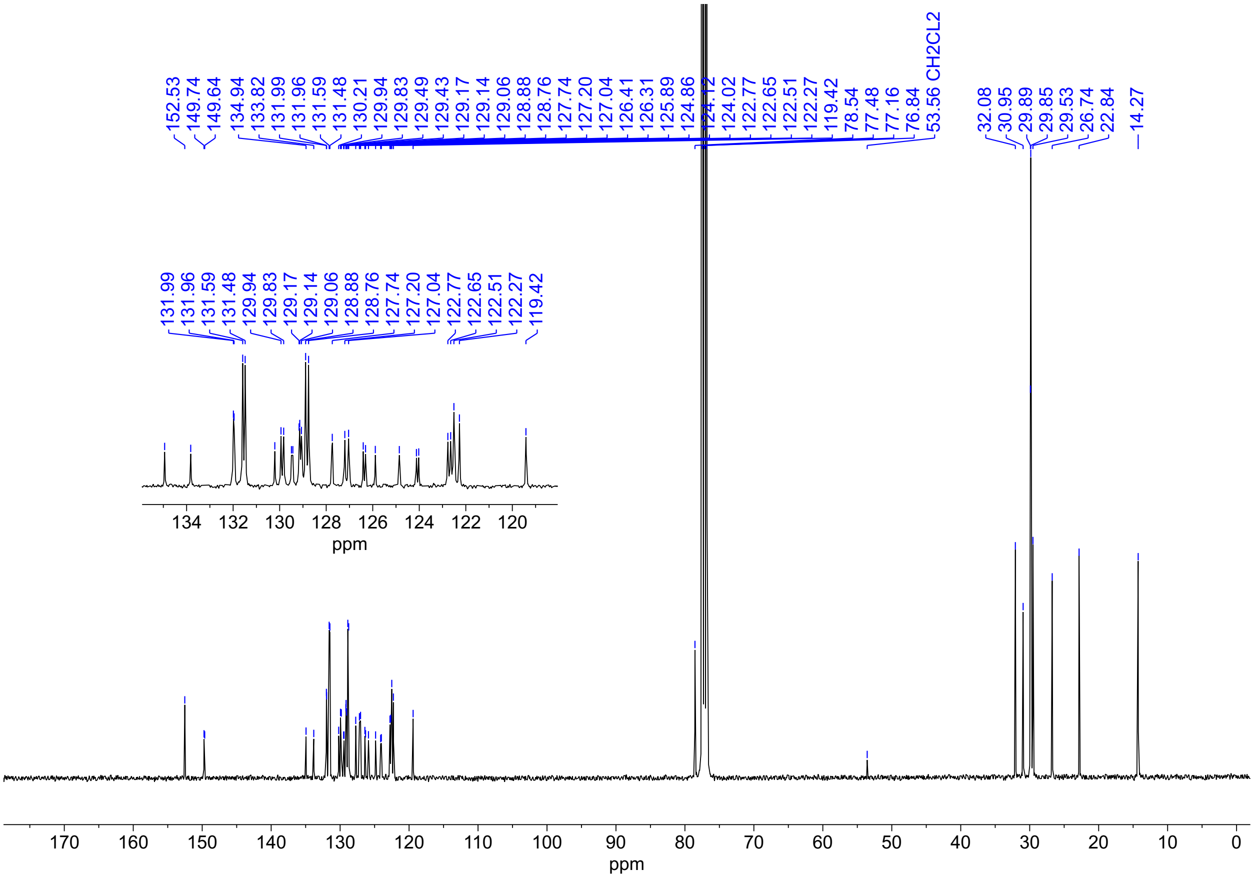

$^{31}P\{^{1}H\}$ NMR spectrum of ***trans*-2-O** measured in $CDCl_3$ (162 MHz).

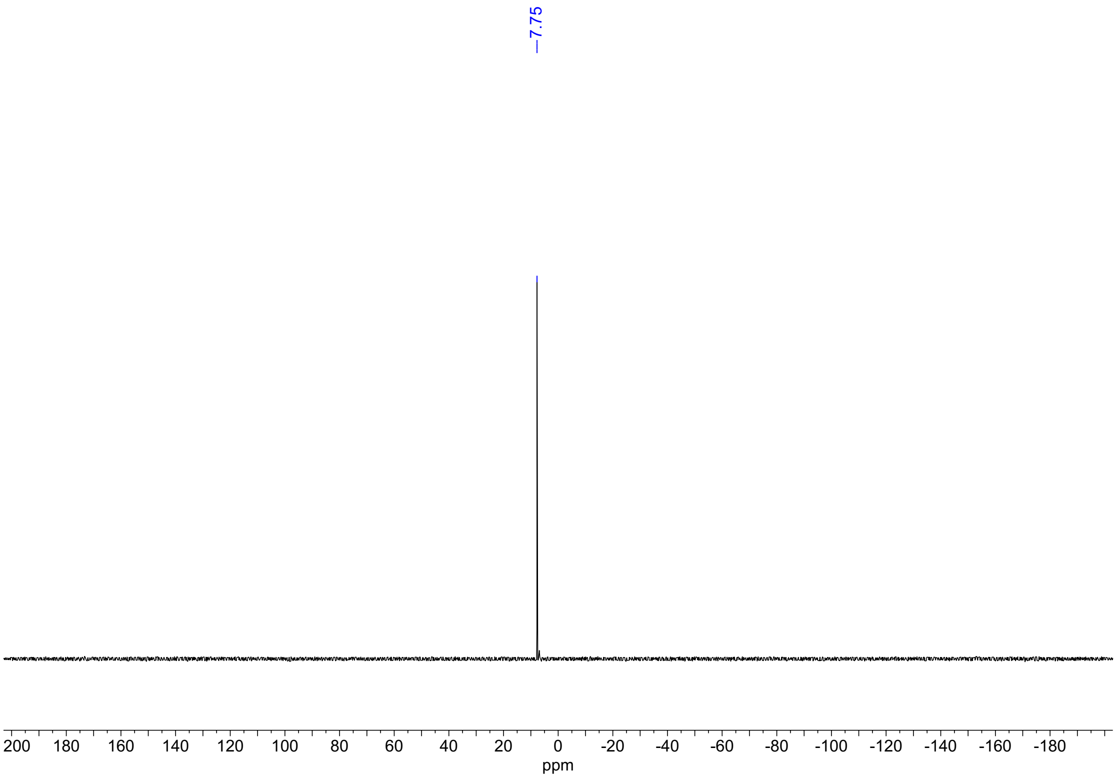

$^{13}C\{^1H\}$ DEPT 135 NMR spectrum of ***trans*-2-O** measured in $CDCl_3$ (101 MHz).

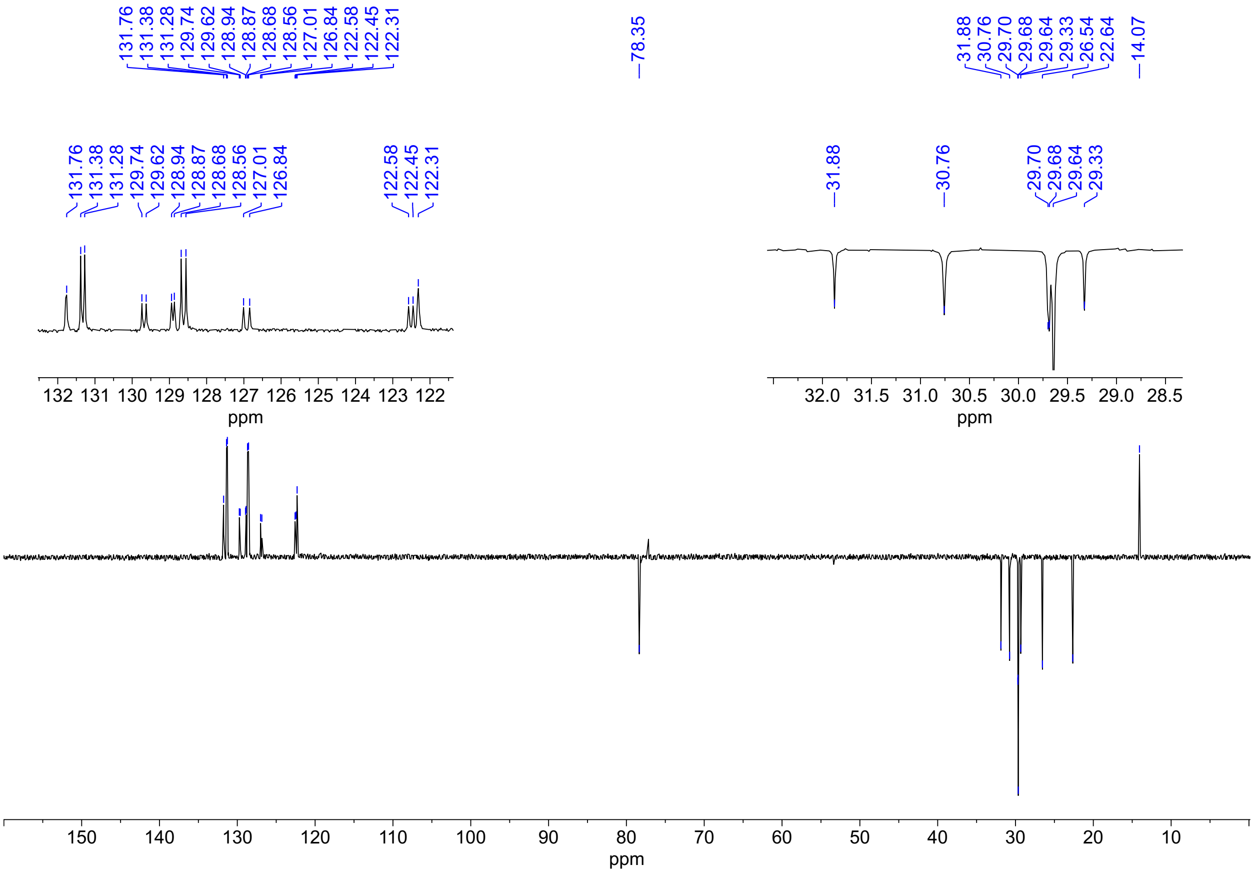

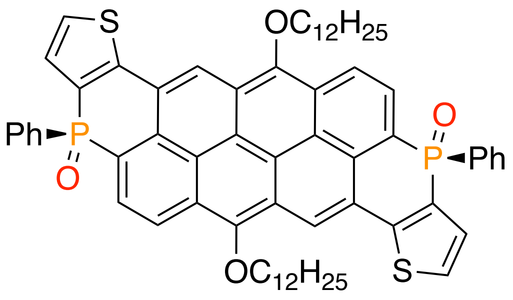

^{1}H NMR spectrum of ***cis*-2-O** measured in $CDCl_3$ (400 MHz).

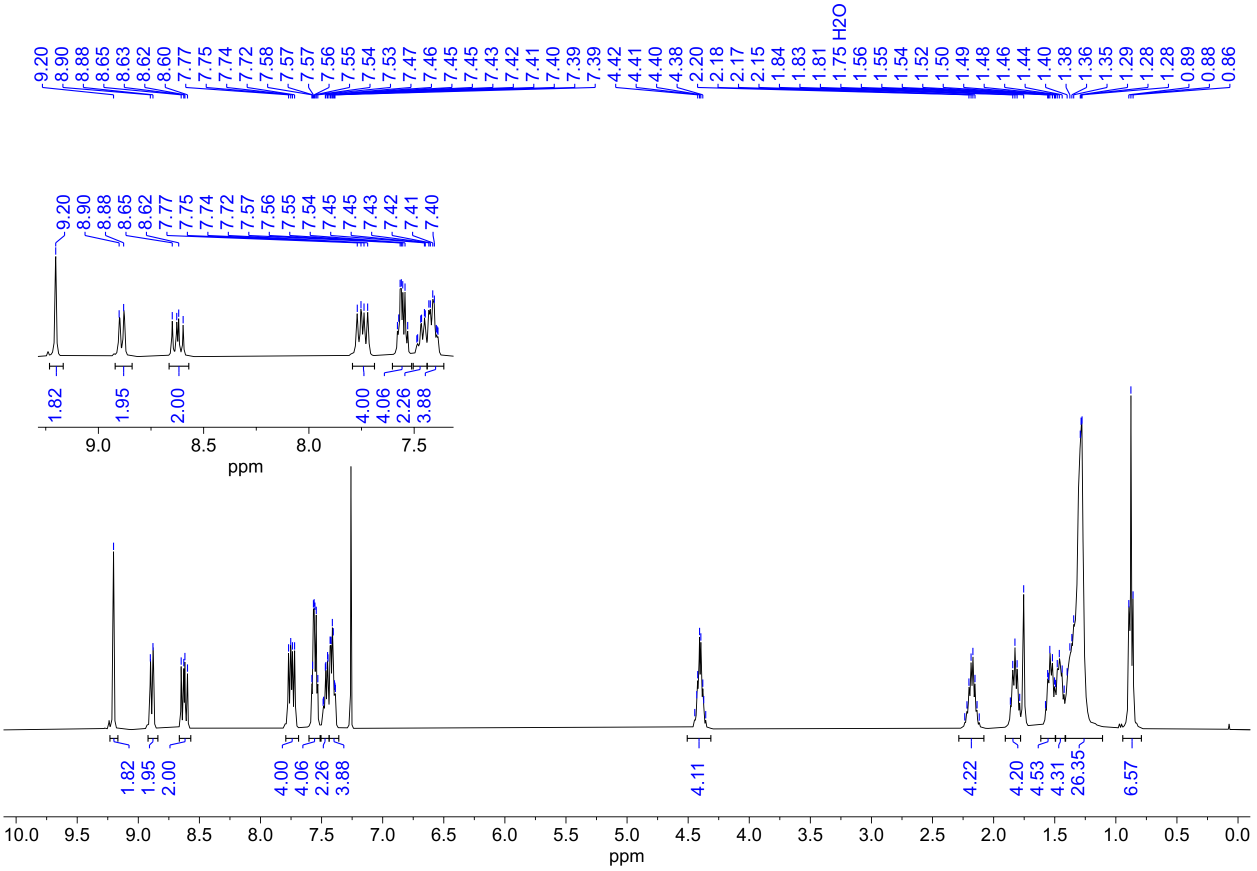

$^{13}C\{^{1}H\}$ NMR spectrum of ***cis*-2-O** measured in $CDCl_3$ (101 MHz).

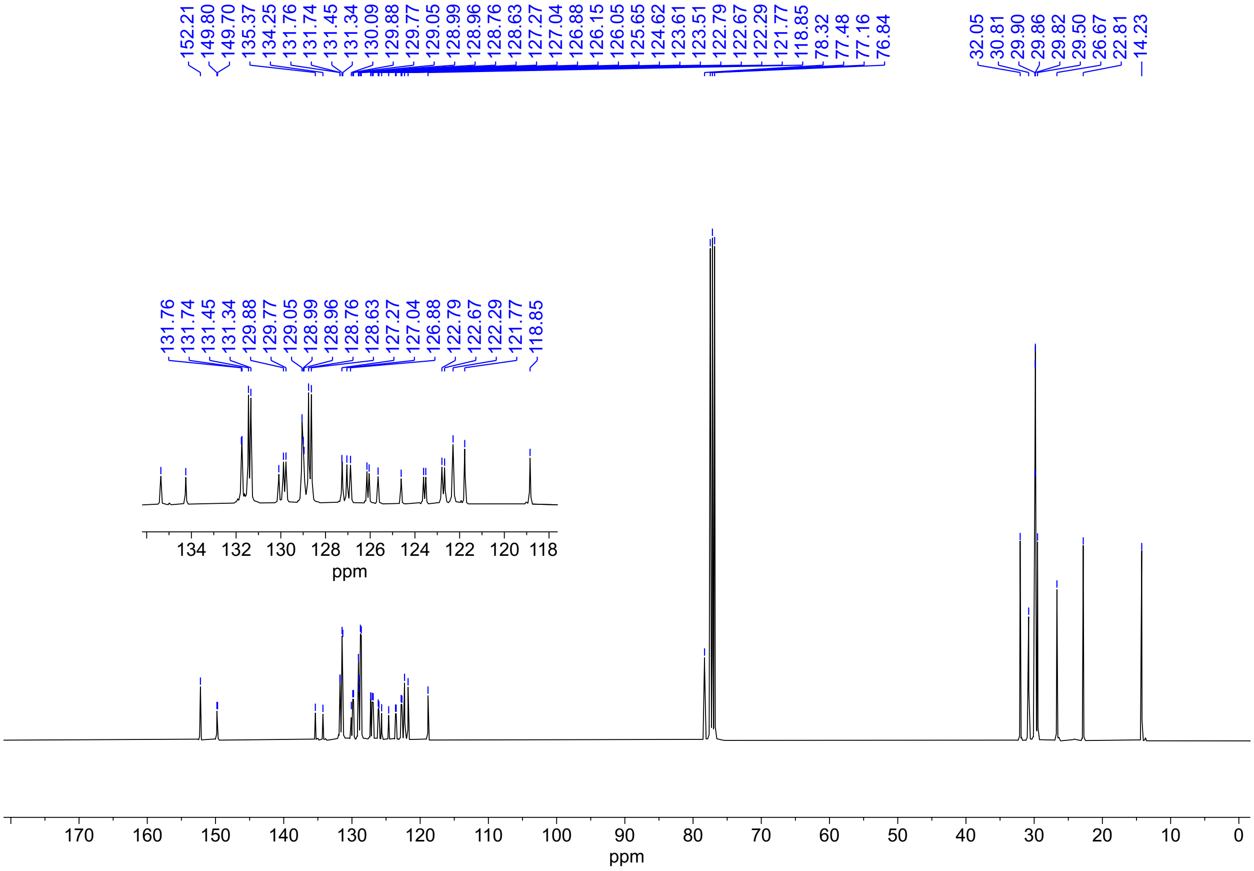

$^{31}P\{^{1}H\}$ NMR spectrum of ***cis*-2-O** measured in $CDCl_3$ (162 MHz).

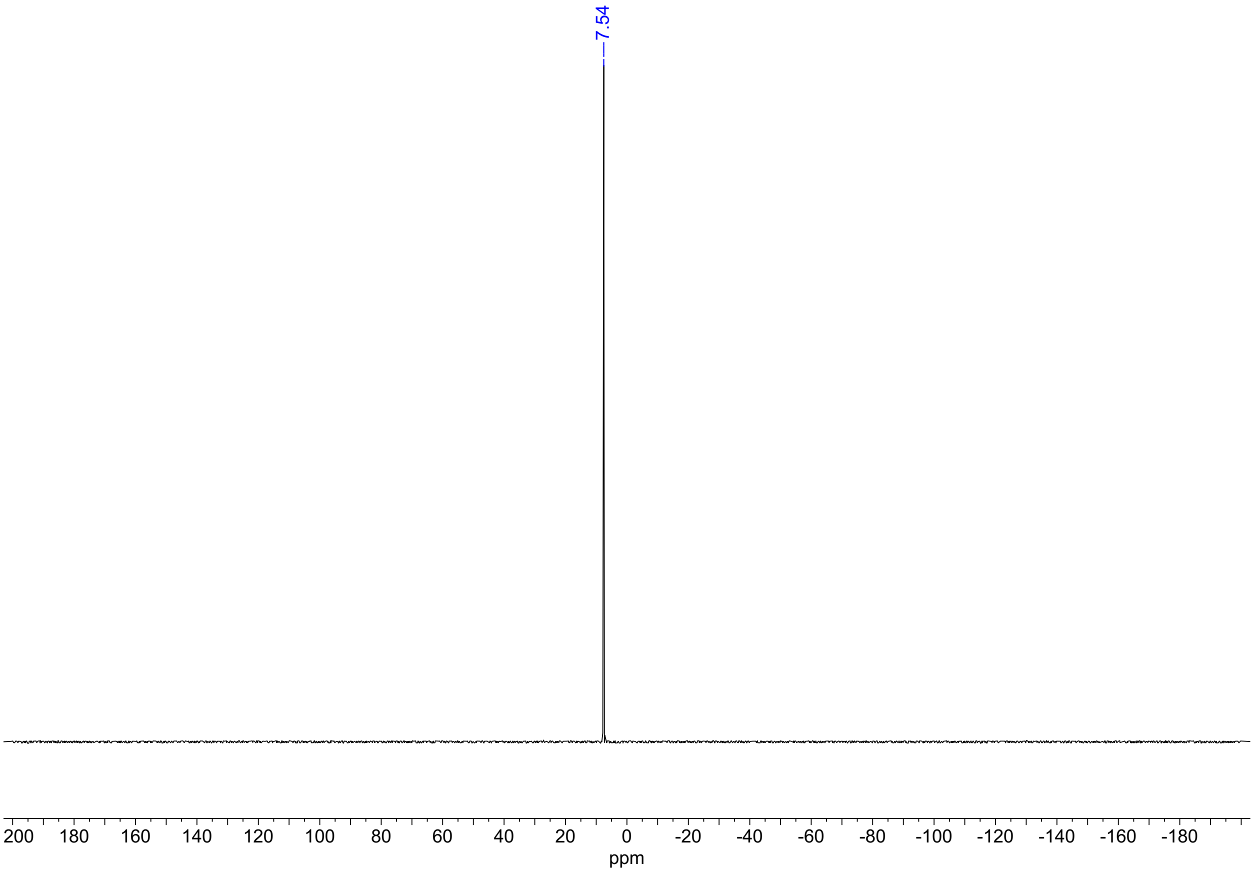

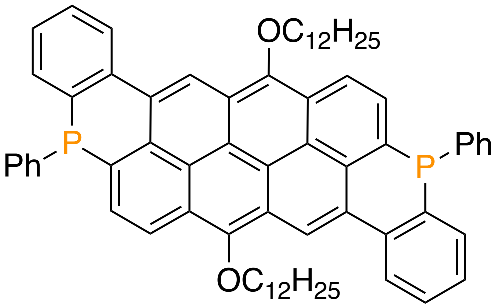

^{1}H NMR spectrum of **1** measured in $CDCl_3$ (400MHz).

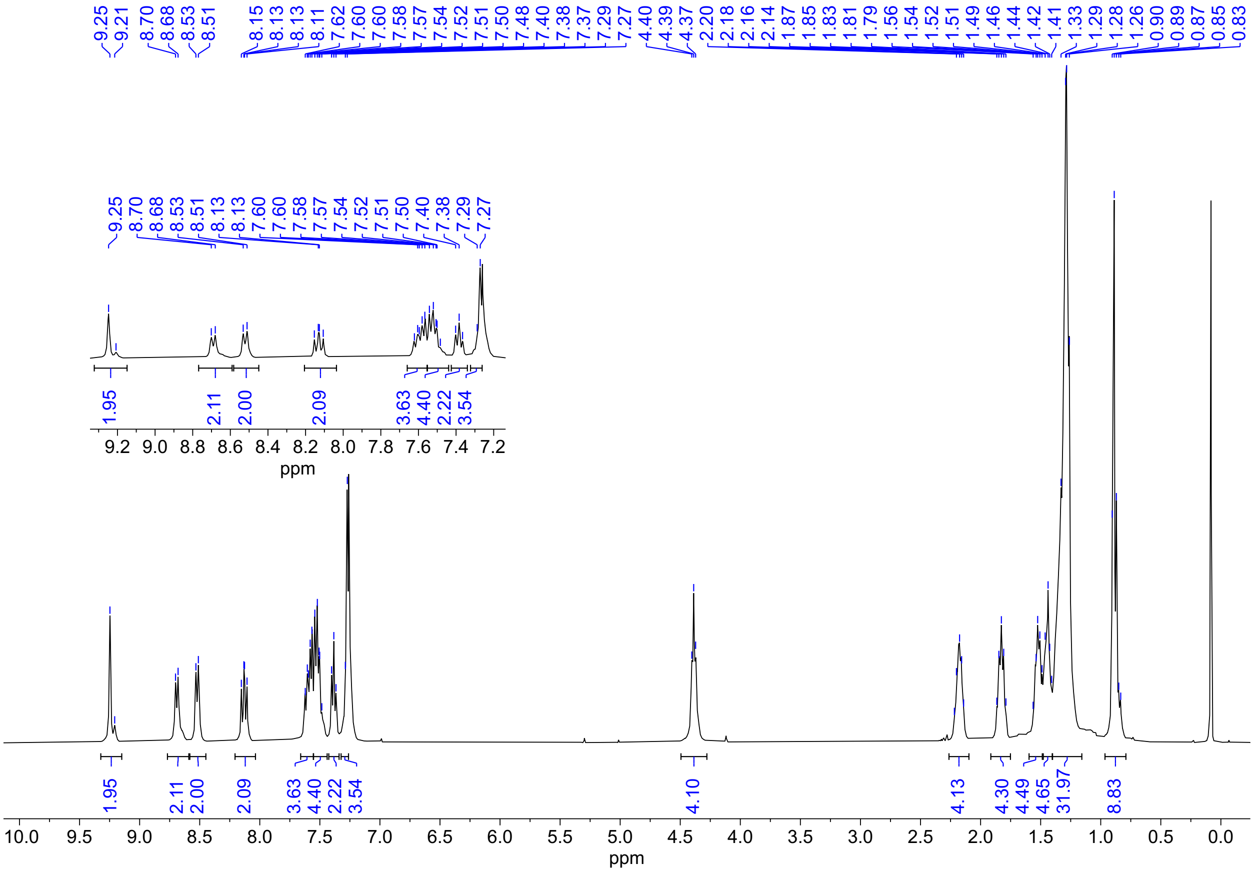

$^{13}C\{^1H\}$ NMR spectrum of **1** measured in $CDCl_3$ (101 MHz).

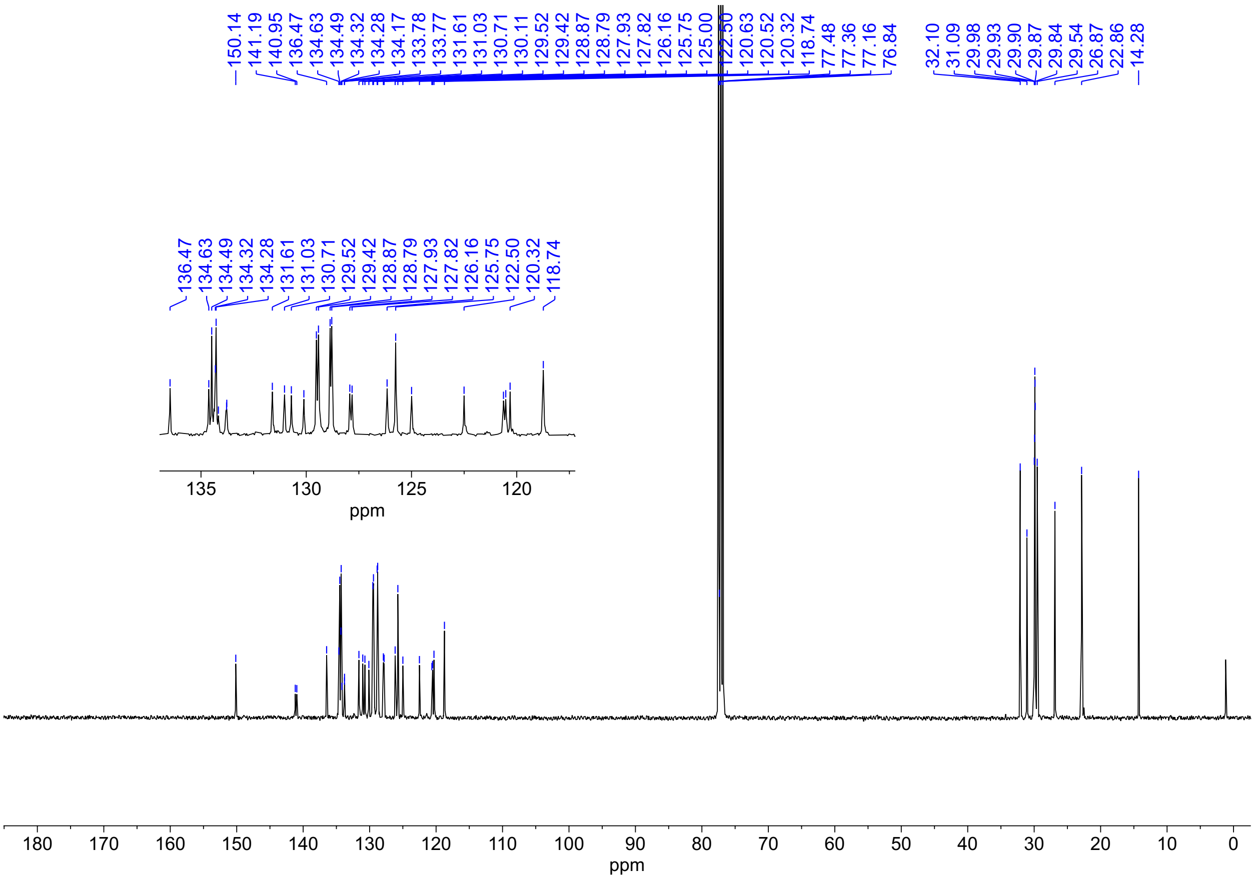

$^{31}P\{^1H\}$ NMR spectrum of **1** measured in $CDCl_3$ (162 MHz).

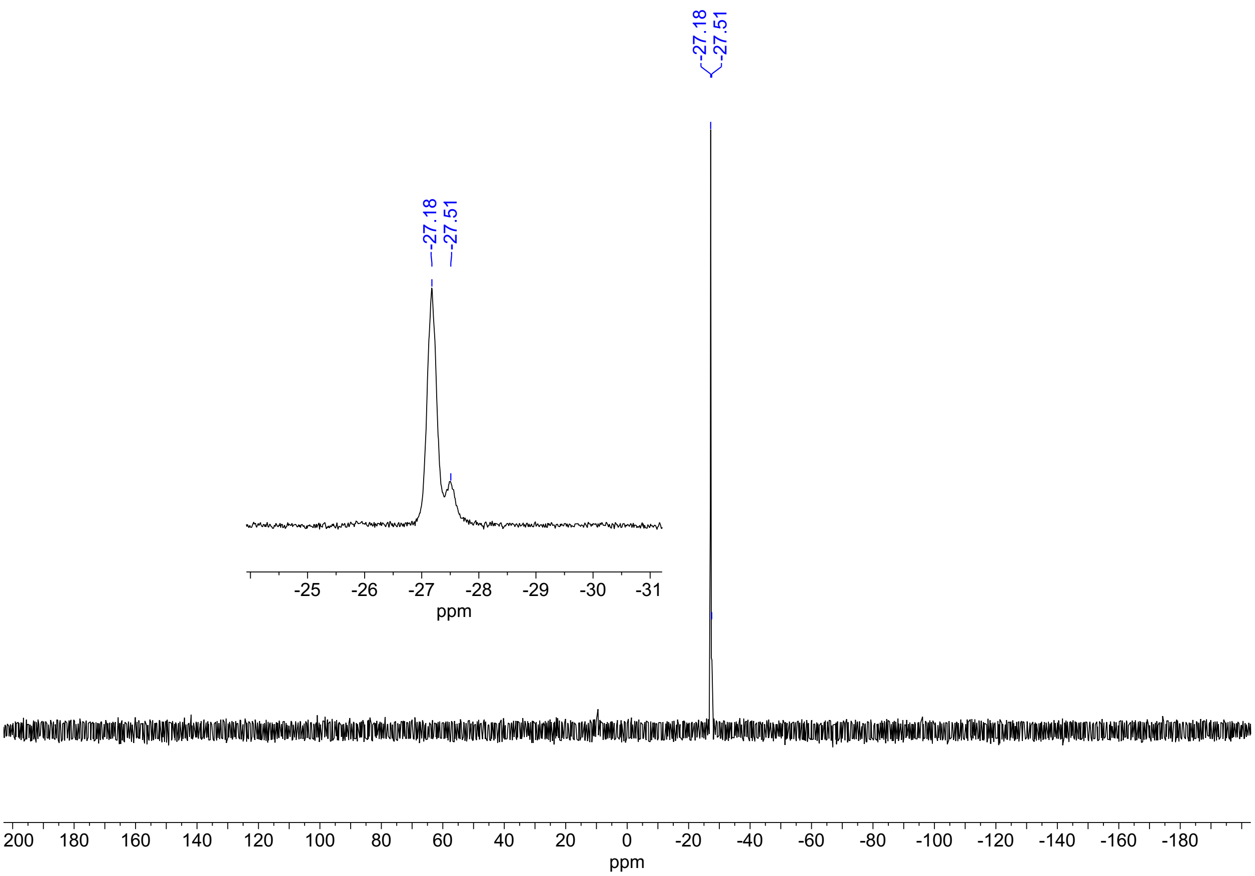

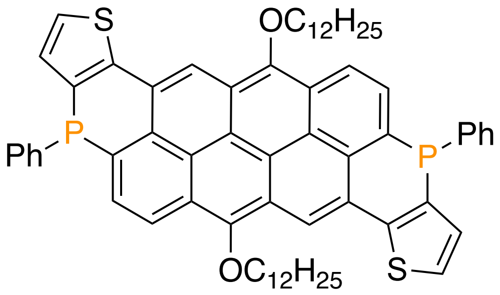

^{1}H NMR spectrum of **2** measured in $CDCl_3$ (400 MHz).

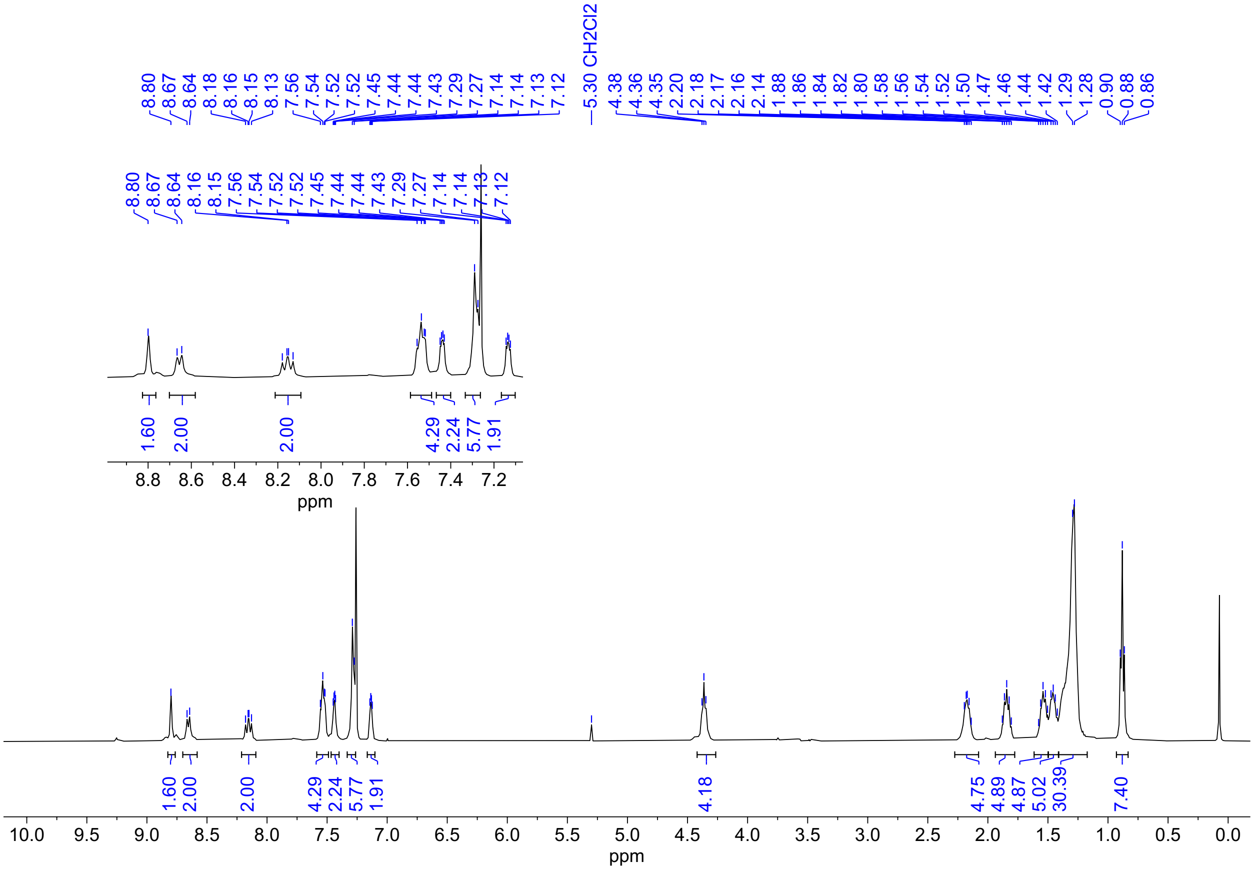

$^{13}C\{^{1}H\}$ NMR spectrum of **2** measured in $CDCl_3$ (101 MHz).

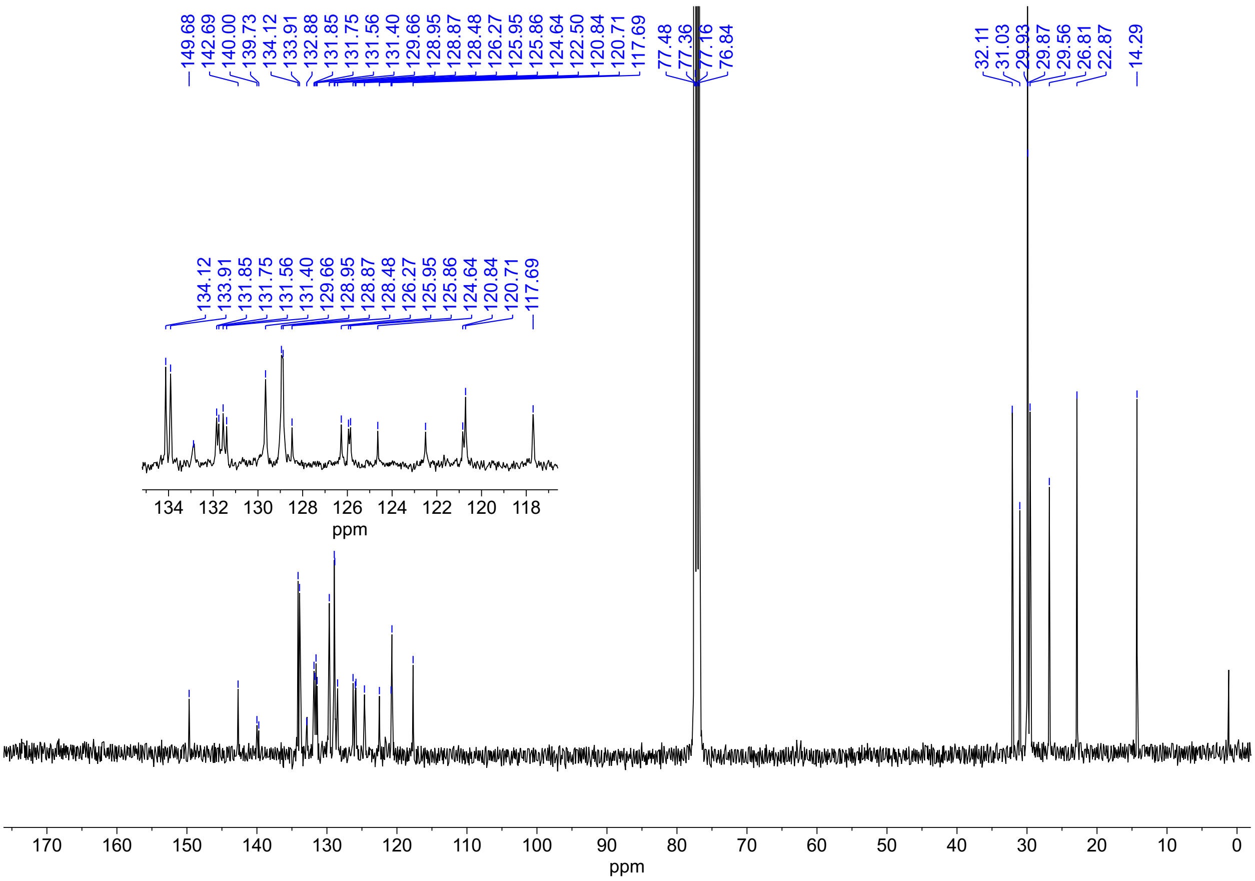

$^{31}P\{^{1}H\}$ NMR spectrum of **2** measured in $CDCl_3$ (162 MHz).

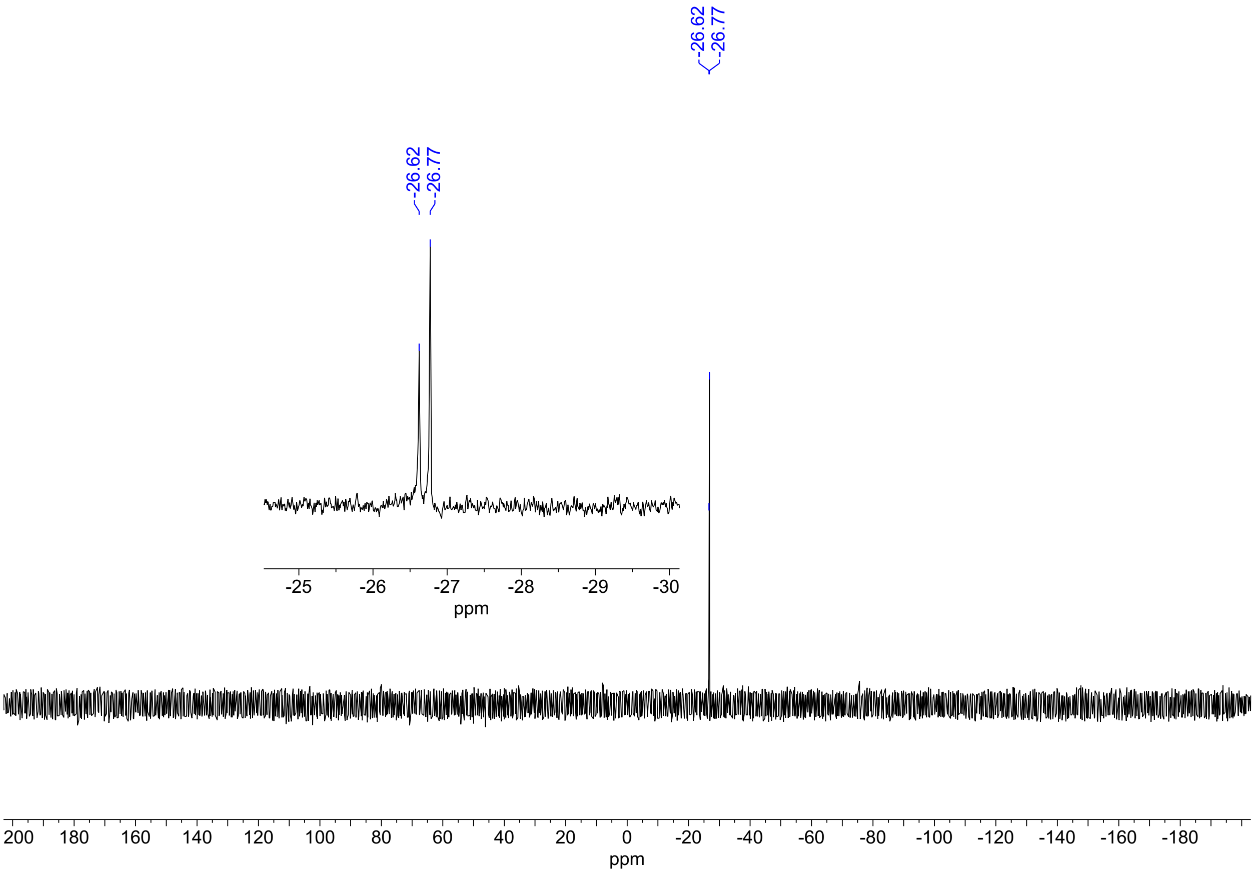

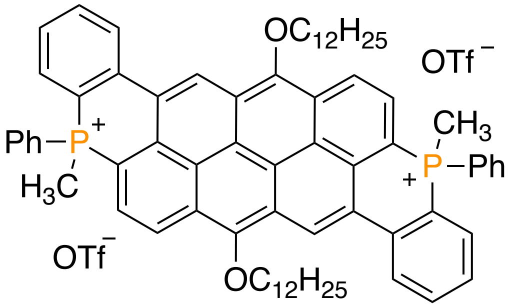

^{1}H NMR spectrum of **1-Me** measured in $CDCl_3$ (400 MHz).

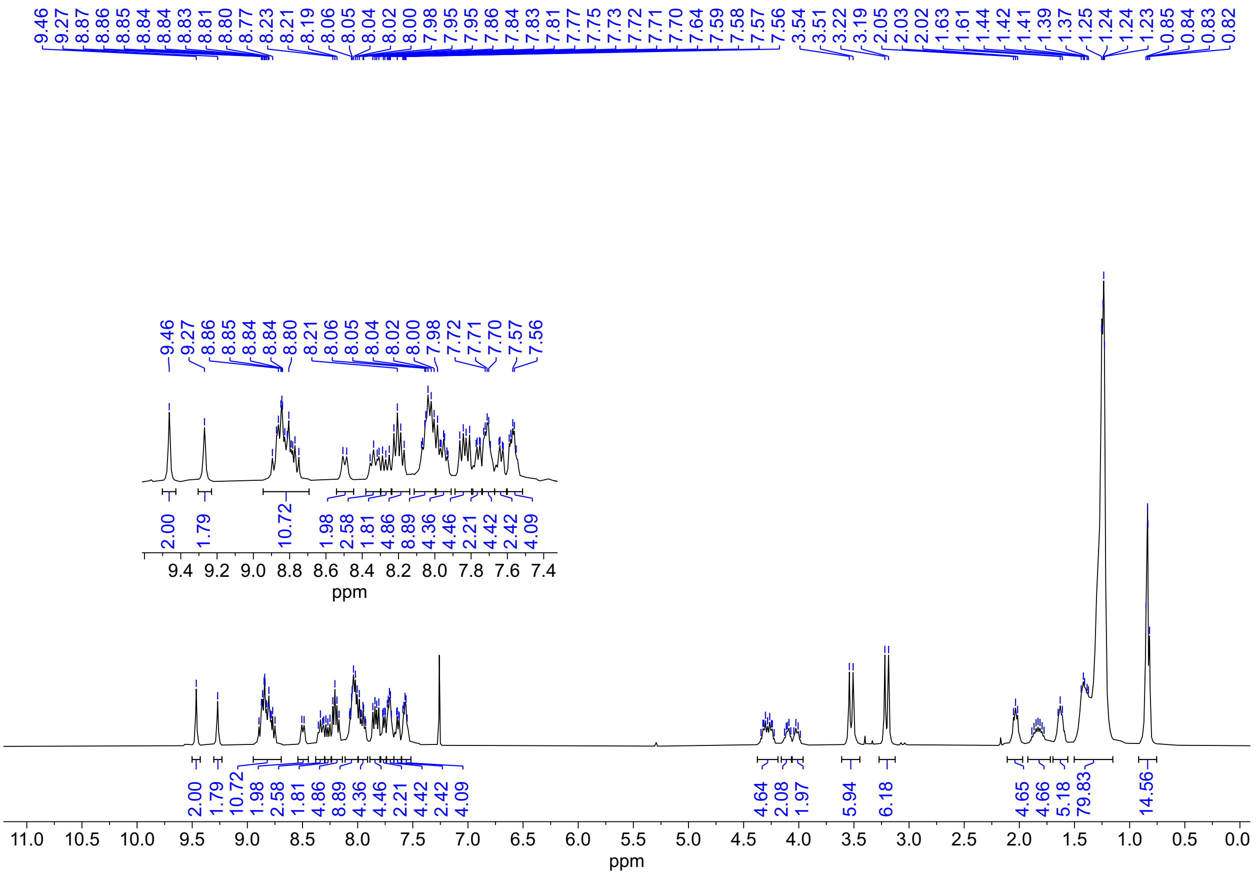

$^{13}C\{^{1}H\}$ NMR spectrum of **1-Me** measured in $CDCl_3$ (101 MHz).

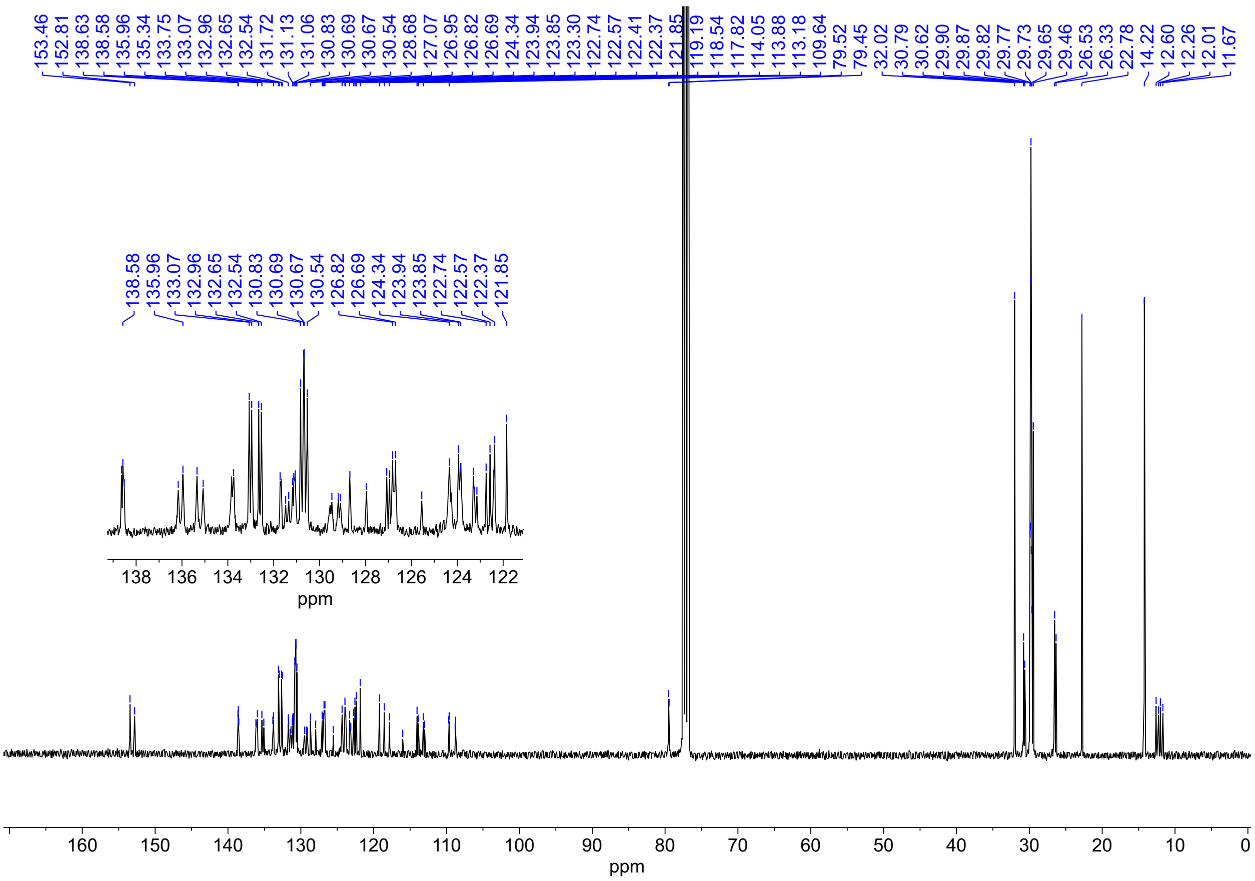

$^{31}P\{^{1}H\}$ NMR spectrum of **1-Me** measured in $CDCl_3$ (162 MHz).

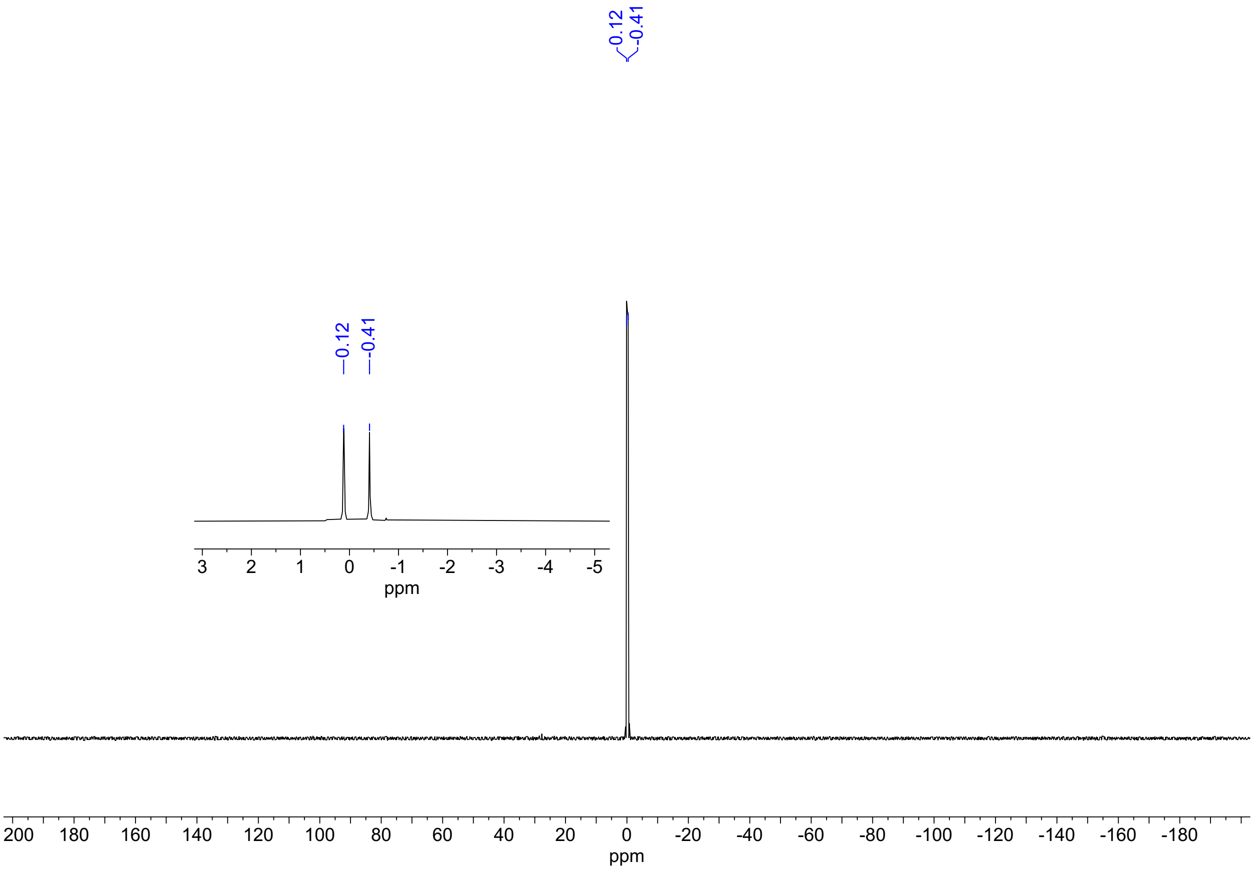

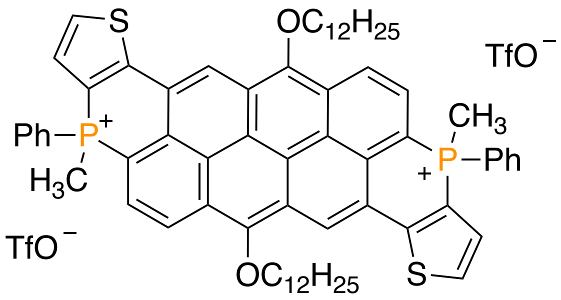

^{1}H NMR spectrum of **2-Me** measured in $CDCl_3$ (400 MHz).

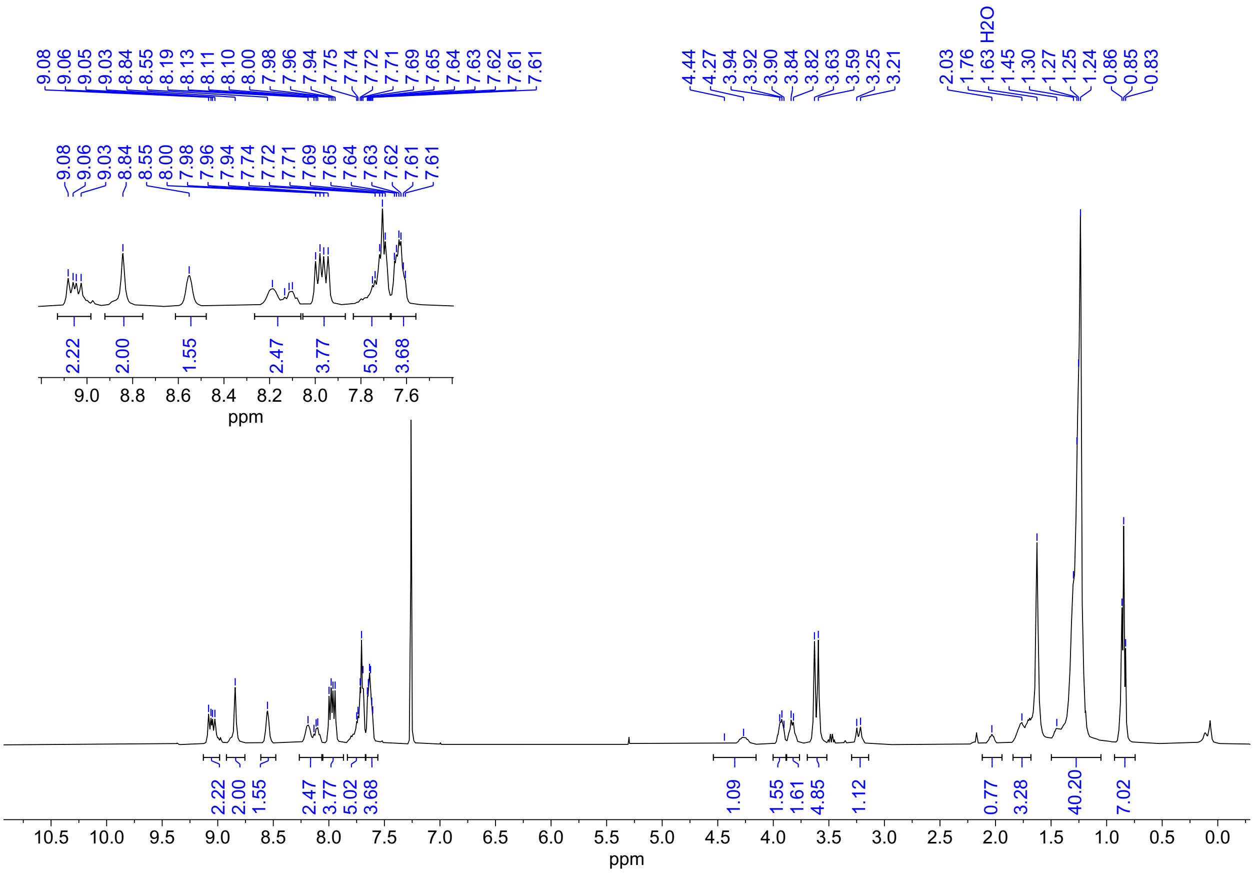

$^{13}C\{^{1}H\}$ NMR spectrum of **2-Me** measured in $CDCl_3$ (101 MHz).

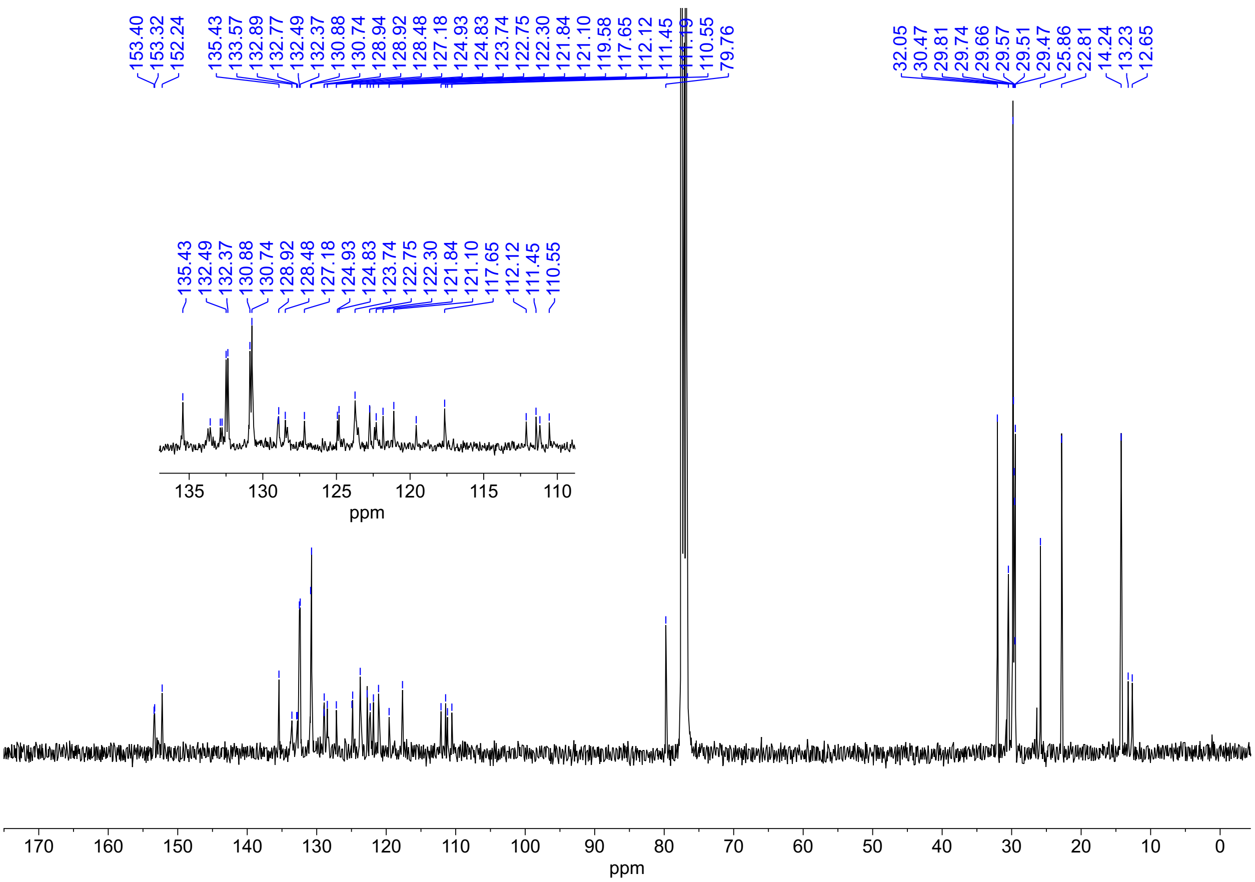

$^{31}P\{^{1}H\}$ NMR spectrum of **2-Me** measured in $CDCl_3$ (162 MHz).

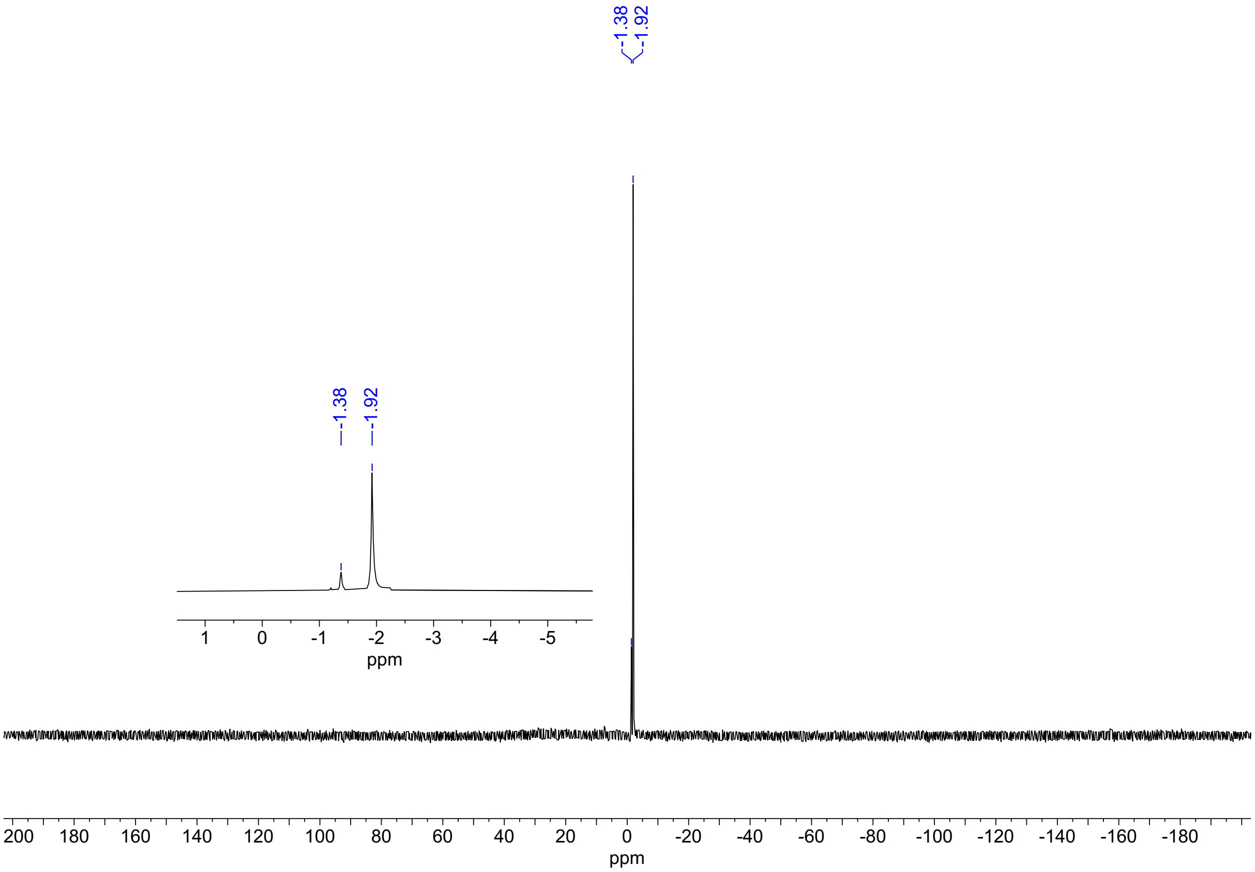

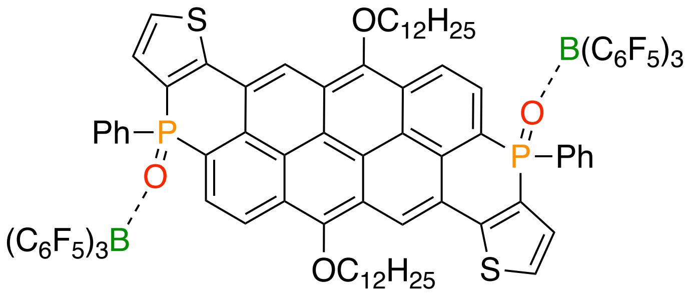

^{1}H NMR spectrum of ***cis*-2-O(BCF)₂** measured in $CDCl_3$ (400 MHz).

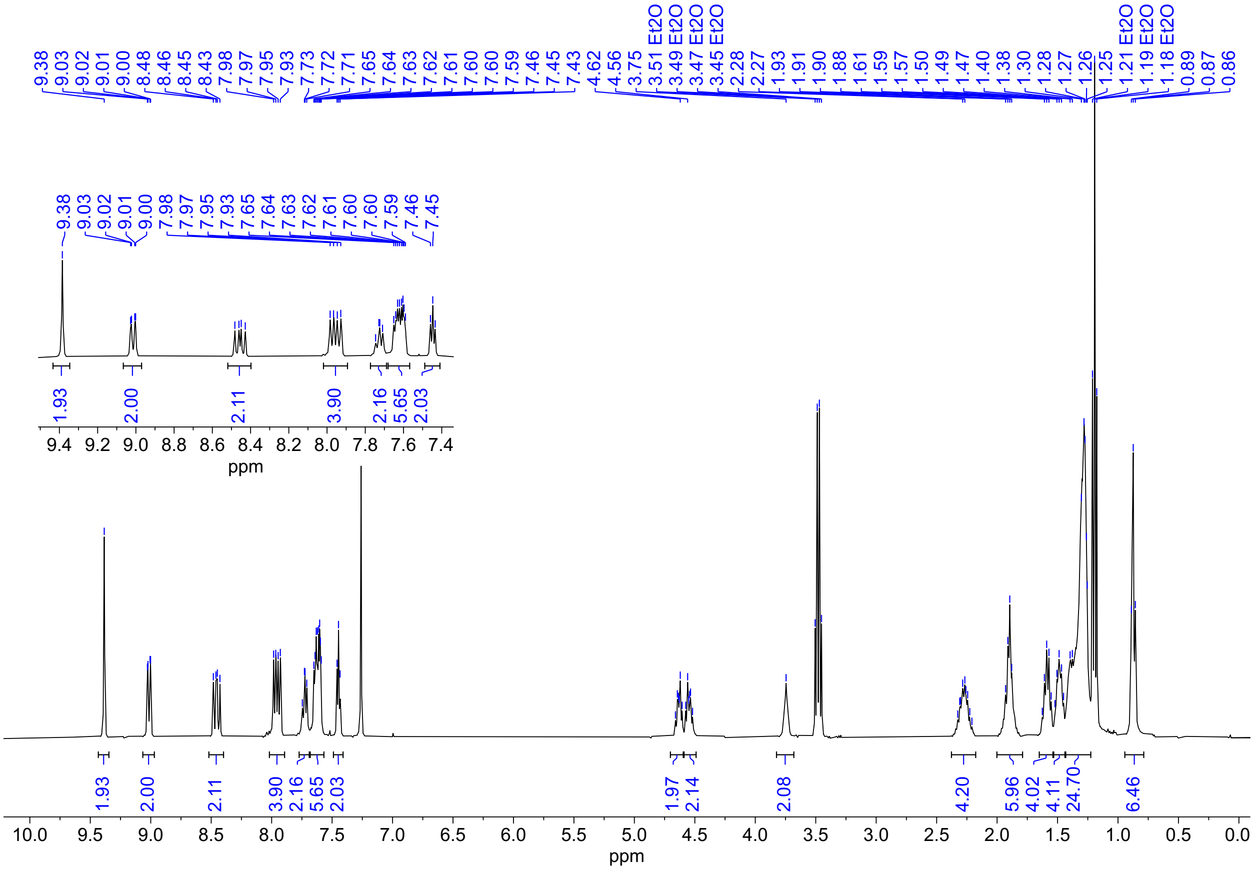

$^{31}P\{^1H\}$ NMR spectrum of ***cis*-2-O(BCF)$_2$** measured in $CDCl_3$ (162 MHz).

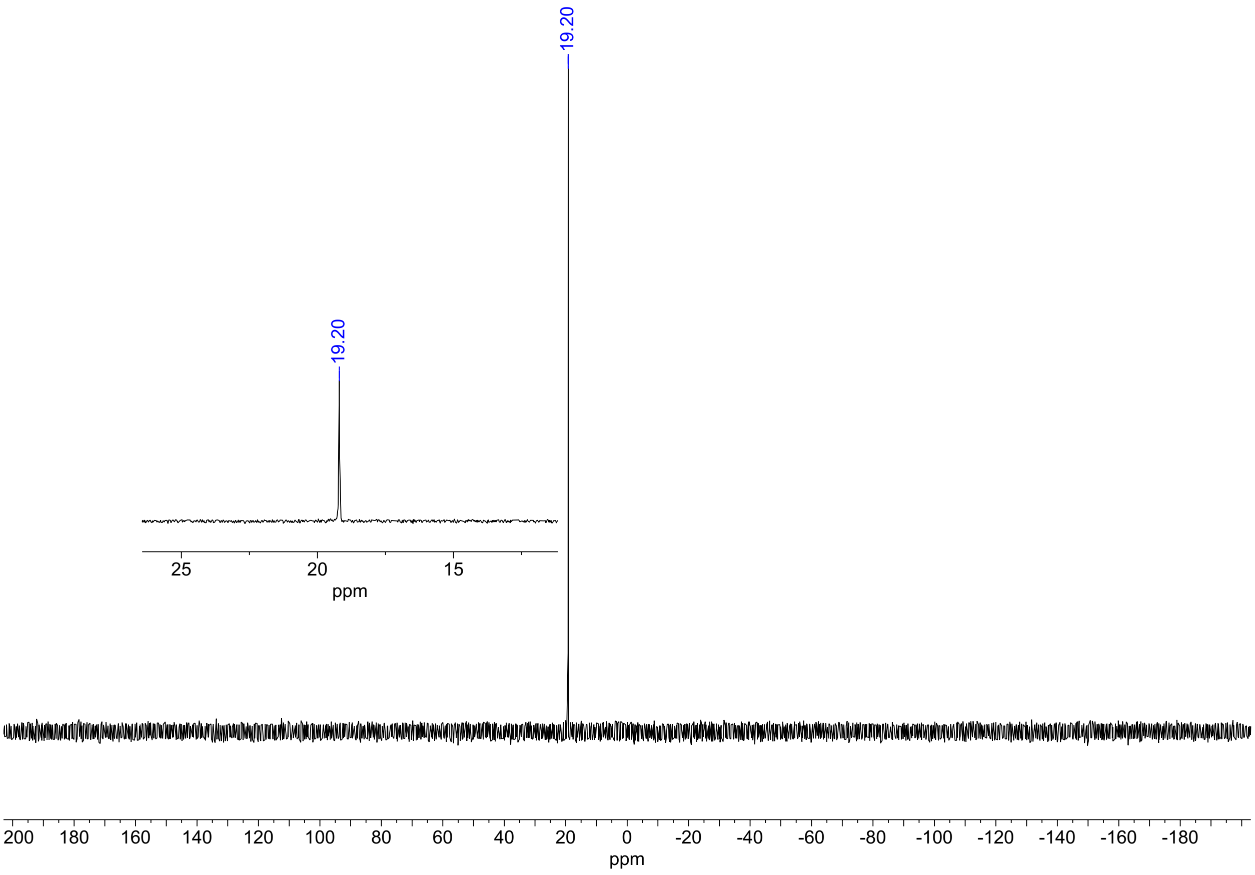

8. Coordinates (in Å) of optimized geometries of all 12 vat compounds and *cis*-2-O(BCF)$_2$

***Trans*-1-O**

C	-3.932480	0.889211	-0.675614
C	-2.837620	0.016246	-0.668045
C	-1.523909	0.589798	-0.532183
C	-1.365834	2.009157	-0.447798
C	-2.506513	2.839426	-0.490818
C	-3.753818	2.283995	-0.597439
C	-0.372357	-0.231601	-0.462902
C	-0.064543	2.557047	-0.308193
C	1.061308	1.746653	-0.223341
C	0.911404	0.331968	-0.304329
C	2.062985	-0.489353	-0.233752
C	3.376966	0.084669	-0.102370
C	3.503923	1.537165	0.016120
C	2.366272	2.289671	-0.046903
H	-2.387551	3.911498	-0.419015
H	-4.623946	2.928755	-0.620095
H	2.418659	3.361088	0.049381
C	1.904439	-1.908984	-0.313059
C	4.472222	-0.788040	-0.090552
C	3.045110	-2.739053	-0.262726
H	2.926017	-3.811568	-0.328046
C	4.292707	-2.183394	-0.158187
H	5.162565	-2.828372	-0.132247
C	0.603158	-2.456805	-0.456397
C	-0.522353	-1.646174	-0.545977
C	-2.964362	-1.435531	-0.795524
C	-1.826886	-2.188369	-0.730219
H	-1.878801	-3.259370	-0.830830
P	-5.639119	0.336602	-0.719453
C	-4.275355	-2.113333	-1.009159
C	-5.506721	-1.430575	-0.984867
C	-6.715664	-2.105426	-1.200931
C	-6.738772	-3.468035	-1.433391
P	6.179399	-0.232649	-0.071479
C	4.815386	2.216872	0.218037
C	6.047253	1.535646	0.184555
C	7.280573	3.576579	0.612921
C	7.257254	2.212942	0.386564
O	0.069451	3.915087	-0.185356
O	0.469348	-3.814981	-0.577910

C	0.361544	4.641066	-1.394401
H	-0.517063	4.678634	-2.042012
H	0.639280	5.646428	-1.084157
H	1.191076	4.178703	-1.932818
C	0.167406	-4.538823	0.630037
H	-0.107893	-5.544734	0.319314
H	1.040844	-4.575272	1.284662
H	-0.666470	-4.075639	1.161010
H	-7.642598	-1.544582	-1.193480
H	-7.676438	-3.981537	-1.603051
H	8.219054	4.092154	0.771240
H	8.184907	1.653469	0.372380
C	6.796555	-0.509453	-1.760602
C	6.216386	0.110107	-2.872586
C	7.848891	-1.408273	-1.942721
C	6.680480	-0.175726	-4.150176
H	5.400589	0.812690	-2.746663
C	8.309999	-1.696169	-3.224018
H	8.299883	-1.879172	-1.079411
C	7.722670	-1.086783	-4.328290
H	6.228037	0.306278	-5.007770
H	9.125792	-2.395948	-3.357410
H	8.079567	-1.316303	-5.324873
O	7.052418	-0.934315	0.920919
O	-6.495672	1.047050	-1.719411
C	-6.285536	0.599704	0.960862
C	-5.688201	0.015530	2.082571
C	-7.388467	1.439526	1.125259
C	-6.184131	0.279145	3.353366
H	-4.833250	-0.641141	1.969623
C	-7.883522	1.702438	2.399086
H	-7.852910	1.883724	0.254919
C	-7.278523	1.128045	3.512366
H	-5.717280	-0.174919	4.218605
H	-8.739702	2.354676	2.519784
H	-7.662169	1.338881	4.503304
C	4.872968	3.603883	0.462657
C	6.072249	4.269507	0.647025
H	3.968586	4.188459	0.526614
H	6.061580	5.335843	0.836335
C	-5.531203	-4.162695	-1.457675
C	-4.332792	-3.499572	-1.258762
H	-5.520270	-5.228371	-1.650808
H	-3.428945	-4.085715	-1.315027

***Cis*-1-O**

C	-4.07639	0.75923	-1.00964
C	-2.92536	0.00610	-0.74726
C	-1.66964	0.70731	-0.67671
C	-1.62104	2.11817	-0.90506
C	-2.81544	2.82135	-1.16768
C	-4.00713	2.15032	-1.21687
C	-0.46690	0.02442	-0.37451
C	-0.37561	2.79639	-0.84642
C	0.80017	2.12786	-0.52882
C	0.75760	0.72119	-0.29360
C	1.96371	0.03364	-0.01880
C	3.22107	0.73254	0.02725
C	3.23003	2.18753	-0.12931
C	2.04681	2.80929	-0.41193
H	-2.78389	3.89108	-1.31570
H	-4.91826	2.70050	-1.41787
H	2.01260	3.87625	-0.56025
C	1.91931	-1.38065	0.19055
C	4.38160	-0.02857	0.21024
C	3.12224	-2.09156	0.38695
H	3.09329	-3.16342	0.52097
C	4.31773	-1.42523	0.38162
H	5.23505	-1.98564	0.51510
C	0.66948	-2.05292	0.17084
C	-0.50946	-1.38038	-0.12920
C	-2.93905	-1.44222	-0.53601
C	-1.75926	-2.05838	-0.22781
H	-1.72848	-3.12051	-0.04897
P	-5.73085	0.06576	-1.03180
C	-4.17993	-2.25974	-0.65857
C	-5.45657	-1.69463	-0.84688
C	-6.59992	-2.49744	-0.94926
C	-6.51488	-3.87452	-0.86379
P	6.03064	0.66757	0.09514
C	4.46854	3.00422	0.01860
C	5.74996	2.43594	0.15673
C	6.79785	4.61763	0.28095
C	6.89081	3.23856	0.28469
O	-0.35219	4.14972	-1.06728
O	0.64314	-3.40671	0.39294
C	-0.28489	4.54499	-2.45205
H	-1.06986	4.06081	-3.03462

H	-0.42540	5.62373	-2.47256
H	0.69016	4.28512	-2.87194
C	0.60180	-3.80408	1.77813
H	0.65610	-4.89055	1.78866
H	1.44706	-3.38739	2.32885
H	-0.33108	-3.47167	2.24025
H	-7.56269	-2.02664	-1.10736
H	-7.40421	-4.48662	-0.93976
H	7.68535	5.22884	0.38208
H	7.86118	2.76751	0.38910
C	6.86945	0.19562	1.63814
C	7.98799	-0.63797	1.58779
C	6.37410	0.61003	2.87877
C	8.60278	-1.05756	2.76546
H	8.37162	-0.95921	0.62799
C	6.98868	0.19136	4.05154
H	5.50580	1.25621	2.93369
C	8.10221	-0.64541	3.99537
H	9.46824	-1.70717	2.72061
H	6.59803	0.51538	5.00833
H	8.57617	-0.97680	4.91131
O	6.78813	0.20834	-1.11300
O	-6.53940	0.44080	-2.23335
C	-6.51477	0.67165	0.49282
C	-6.08217	0.24559	1.75161
C	-7.52534	1.63050	0.40068
C	-6.65199	0.77739	2.90160
H	-5.30132	-0.50114	1.83951
C	-8.09308	2.16523	1.55444
H	-7.86274	1.95600	-0.57495
C	-7.65503	1.74021	2.80424
H	-6.31141	0.44086	3.87307
H	-8.87422	2.91139	1.47486
H	-8.09401	2.15711	3.70219
C	4.40526	4.41264	0.03543
C	5.53785	5.19999	0.15748
H	3.45492	4.91934	-0.03671
H	5.43496	6.27832	0.16502
C	-5.25963	-4.45456	-0.69051
C	-4.12439	-3.66733	-0.59771
H	-5.16242	-5.53188	-0.63201
H	-3.17898	-4.17496	-0.48303

***Trans*-1**

C	-3.830484	0.912218	-0.503612
C	-2.739011	0.039012	-0.577738
C	-1.415402	0.592055	-0.434739
C	-1.242922	1.987210	-0.173582
C	-2.381611	2.815074	-0.078024
C	-3.630530	2.286206	-0.262430
C	-0.263202	-0.225506	-0.547505
C	0.063772	2.513686	-0.022986
C	1.191896	1.712633	-0.138250
C	1.031754	0.321508	-0.406487
C	2.183991	-0.496014	-0.519234
C	3.507638	0.056866	-0.375315
C	3.645692	1.496086	-0.151714
C	2.508987	2.240912	-0.016991
H	-2.259715	3.867165	0.137945
H	-4.493351	2.940983	-0.212747
H	2.572582	3.293573	0.200056
C	2.011620	-1.890966	-0.781650
C	4.599142	-0.816284	-0.450485
C	3.150295	-2.718699	-0.878254
H	3.028371	-3.770564	-1.095335
C	4.399238	-2.190004	-0.693413
H	5.262067	-2.844731	-0.743576
C	0.704942	-2.417524	-0.931942
C	-0.423273	-1.616666	-0.815655
C	-2.876980	-1.400404	-0.799948
C	-1.740391	-2.145169	-0.935931
H	-1.804049	-3.197776	-1.153461
P	-5.556633	0.433258	-0.790726
C	-4.203216	-2.077926	-0.865935
C	-5.423669	-1.377825	-0.850388
C	-6.640478	-2.069512	-0.937362
C	-6.684543	-3.449850	-1.005209
P	6.324836	-0.337766	-0.161072
C	4.972054	2.173619	-0.084321
C	6.192399	1.473405	-0.100659
C	7.453791	3.545106	0.054367
C	7.409445	2.164793	-0.014242
O	0.208337	3.843914	0.295930
O	0.560257	-3.747634	-1.251466
C	0.363616	4.745625	-0.811226
H	-0.552762	4.798070	-1.403397
H	0.580146	5.721180	-0.379573
H	1.191204	4.436201	-1.453602

C	0.407058	-4.650011	-0.144498
H	0.191131	-5.625617	-0.576356
H	1.324008	-4.701792	0.446828
H	-0.420285	-4.341650	0.498691
H	-7.566606	-1.505328	-0.946791
H	-7.633872	-3.967279	-1.065909
H	8.403247	4.062349	0.114661
H	8.335520	1.600460	-0.005917
C	7.064833	-0.631451	-1.833669
C	6.500791	-0.111120	-3.003991
C	8.206925	-1.428925	-1.927012
C	7.063652	-0.393600	-4.241626
H	5.614497	0.509776	-2.950173
C	8.774361	-1.711523	-3.169045
H	8.653453	-1.835223	-1.026965
C	8.200253	-1.198199	-4.325858
H	6.615842	0.010632	-5.141549
H	9.659646	-2.332647	-3.229341
H	8.634545	-1.423904	-5.292243
C	-6.293976	0.728161	0.882794
C	-5.729526	0.207470	2.052752
C	-7.433857	1.528731	0.977167
C	-6.289663	0.492767	3.291010
H	-4.844664	-0.415389	1.998311
C	-7.998482	1.814257	2.219817
H	-7.880417	1.935596	0.077406
C	-7.423867	1.300534	3.376229
H	-5.841550	0.088280	4.190669
H	-8.881963	2.437854	2.280955
H	-7.856088	1.528335	4.343066
C	5.048195	3.581256	-0.017557
C	6.254559	4.255301	0.048704
H	4.149624	4.178039	-0.028671
H	6.259221	5.337427	0.099222
C	-5.485213	-4.159889	-0.998096
C	-4.279069	-3.485589	-0.931609
H	-5.489656	-5.242050	-1.047987
H	-3.380346	-4.082136	-0.919506

***Cis*-1**

C	-4.06286	0.75861	-0.94423
C	-2.90405	0.00511	-0.71704
C	-1.64492	0.70528	-0.65240
C	-1.59919	2.12369	-0.82904

C	-2.80125	2.83232	-1.03519
C	-3.98884	2.15688	-1.10068
C	-0.43127	0.01437	-0.40910
C	-0.35379	2.79889	-0.78979
C	0.83249	2.12469	-0.53384
C	0.79746	0.71011	-0.34407
C	2.01340	0.01665	-0.12263
C	3.27403	0.71549	-0.08044
C	3.27291	2.17551	-0.19448
C	2.08262	2.80293	-0.43210
H	-2.77611	3.90735	-1.14296
H	-4.90184	2.71400	-1.27770
H	2.04132	3.87385	-0.54526
C	1.97049	-1.40254	0.04565
C	4.44294	-0.04260	0.05969
C	3.18088	-2.11504	0.18713
H	3.15850	-3.19177	0.28070
C	4.37435	-1.44652	0.17197
H	5.29520	-2.01259	0.25529
C	0.72172	-2.07377	0.04180
C	-0.46722	-1.39836	-0.20591
C	-2.91176	-1.44768	-0.53157
C	-1.72199	-2.07191	-0.27822
H	-1.68546	-3.13727	-0.12124
P	-5.73891	0.07683	-1.09976
C	-4.16619	-2.25483	-0.59999
C	-5.44139	-1.68173	-0.77352
C	-6.58776	-2.48905	-0.80463
C	-6.51317	-3.86138	-0.65929
P	6.12407	0.63608	-0.02215
C	4.51461	2.98887	-0.04243
C	5.79461	2.41745	0.08575
C	6.83257	4.61030	0.23425
C	6.92887	3.23167	0.21292
O	-0.34107	4.16323	-0.96913
O	0.70591	-3.43632	0.23398
C	-0.31037	4.59481	-2.34175
H	-1.10511	4.11892	-2.91887
H	-0.46120	5.67275	-2.33268
H	0.65599	4.35672	-2.79422
C	0.73200	-3.85707	1.61050
H	0.86997	-4.93671	1.60372
H	1.55660	-3.38613	2.14859
H	-0.21099	-3.60494	2.10323

H	-7.55409	-2.02089	-0.95666
H	-7.41065	-4.46667	-0.67964
H	7.72051	5.22114	0.33746
H	7.90428	2.76451	0.29378
C	6.77041	0.20244	1.65644
C	7.90105	-0.61429	1.72756
C	6.14946	0.60733	2.84375
C	8.40115	-1.03130	2.95945
H	8.38975	-0.93301	0.81436
C	6.64843	0.19407	4.07218
H	5.27123	1.24130	2.80914
C	7.77304	-0.62929	4.13229
H	9.27584	-1.66899	3.00006
H	6.15926	0.51077	4.98554
H	8.15667	-0.95535	5.09156
C	-6.51112	0.69206	0.46753
C	-6.11854	0.24757	1.73401
C	-7.49083	1.68281	0.37266
C	-6.68826	0.79232	2.87825
H	-5.36306	-0.52334	1.83000
C	-8.05836	2.23548	1.52021
H	-7.81126	2.03022	-0.60304
C	-7.65629	1.79071	2.77396
H	-6.37211	0.44047	3.85311
H	-8.81268	3.00800	1.43085
H	-8.09452	2.21735	3.66783
C	4.44650	4.39837	-0.01257
C	5.57189	5.19314	0.11925
H	3.49368	4.90059	-0.08375
H	5.46427	6.27095	0.13521
C	-5.25758	-4.44709	-0.50863
C	-4.11841	-3.66121	-0.48569
H	-5.16462	-5.52213	-0.41239
H	-3.17266	-4.16910	-0.37555

***Trans*-1-Me**

C	-3.917325	0.907495	-0.210578
C	-2.805883	0.017504	-0.427129
C	-1.486359	0.570919	-0.356708
C	-1.285828	1.973787	-0.047058
C	-2.438565	2.805013	0.172434
C	-3.686316	2.280248	0.084464
C	-0.341216	-0.244694	-0.581984
C	-0.002666	2.476636	0.034994

C	1.150201	1.674377	-0.188103
C	0.974785	0.296444	-0.504064
C	2.119987	-0.521389	-0.721257
C	3.440287	0.024693	-0.621646
C	3.588184	1.423303	-0.338323
C	2.442497	2.188733	-0.113177
H	-2.296338	3.847089	0.420467
H	-4.539006	2.930040	0.256119
H	2.533555	3.226088	0.161732
C	1.919578	-1.920239	-1.048371
C	4.553118	-0.868322	-0.821993
C	3.072975	-2.748155	-1.277946
H	2.930512	-3.782601	-1.555741
C	4.321855	-2.232130	-1.156062
H	5.175062	-2.881095	-1.327876
C	0.636374	-2.421909	-1.135532
C	-0.516194	-1.622824	-0.897459
C	-2.952751	-1.382703	-0.703511
C	-1.807279	-2.141777	-0.952600
H	-1.897221	-3.179527	-1.225596
P	-5.556397	0.395761	-0.375588
C	-4.264047	-2.052706	-0.717718
C	-5.499668	-1.365961	-0.618781
C	-6.723774	-2.054930	-0.654553
C	-6.769245	-3.428474	-0.773006
P	6.181225	-0.380424	-0.525893
C	4.902397	2.085990	-0.293590
C	6.134698	1.388778	-0.333389
C	7.412982	3.445389	-0.180362
C	7.362241	2.069539	-0.270569
O	0.191589	3.804228	0.398295
O	0.440856	-3.745941	-1.511973
C	0.306041	4.721739	-0.693407
H	-0.642888	4.814712	-1.229224
H	0.577595	5.686165	-0.263962
H	1.082970	4.405616	-1.394721
C	0.347117	-4.677667	-0.430280
H	0.075947	-5.638368	-0.868225
H	1.303644	-4.771200	0.091660
H	-0.422398	-4.375002	0.284889
H	-7.651762	-1.501588	-0.571608
H	-7.718162	-3.948552	-0.798460
H	8.364123	3.959965	-0.133591
H	8.287934	1.507307	-0.308946

C	7.297815	-0.787028	-1.910757
C	6.813972	-0.622738	-3.213805
C	8.634798	-1.146974	-1.719740
C	7.657482	-0.791598	-4.303737
H	5.776574	-0.354261	-3.371956
C	9.479533	-1.307406	-2.814035
H	9.031668	-1.297620	-0.725000
C	8.994503	-1.125297	-4.105194
H	7.271100	-0.657756	-5.306603
H	10.515724	-1.579113	-2.655948
H	9.653948	-1.248127	-4.955352
C	-6.559314	0.751308	1.111128
C	-5.930532	0.669628	2.357970
C	-7.935599	0.996099	1.055368
C	-6.665520	0.815495	3.527953
H	-4.863832	0.487980	2.410982
C	-8.671096	1.129010	2.229638
H	-8.445988	1.080999	0.105296
C	-8.039031	1.034586	3.465568
H	-6.166416	0.751179	4.486898
H	-9.737273	1.311913	2.176660
H	-8.613501	1.138079	4.377792
C	5.000464	3.496636	-0.224167
C	6.210702	4.158254	-0.166542
H	4.104712	4.097258	-0.231834
H	6.221850	5.240704	-0.117180
C	-5.563320	-4.131678	-0.844211
C	-4.356562	-3.462347	-0.815468
H	-5.568672	-5.212728	-0.918233
H	-3.458110	-4.057154	-0.856815
C	-6.435462	1.194367	-1.757475
H	-7.438865	0.789298	-1.889644
H	-6.500473	2.267947	-1.574604
H	-5.853721	1.014352	-2.661495
C	6.933223	-1.143243	0.951423
H	7.026360	-2.220194	0.805657
H	7.913037	-0.717946	1.169017
H	6.264593	-0.948181	1.789842

***Cis*-1-Me**

C	-4.001629	0.752322	-0.408660
C	-2.835894	-0.018377	-0.291831
C	-1.590572	0.690802	-0.191561
C	-1.578253	2.119837	-0.143026

C	-2.794581	2.831601	-0.180780
C	-3.975587	2.157938	-0.336477
C	-0.361315	-0.007888	-0.152233
C	-0.339008	2.807078	-0.057940
C	0.867131	2.117434	-0.033623
C	0.860285	0.693542	-0.082429
C	2.092229	-0.001323	-0.075727
C	3.341060	0.712754	-0.036385
C	3.326785	2.175045	0.007918
C	2.114172	2.801877	0.022969
H	-2.791231	3.908104	-0.094696
H	-4.900356	2.716790	-0.397442
H	2.051868	3.875507	0.084764
C	2.077889	-1.430688	-0.121883
C	4.513342	-0.055130	-0.041199
C	3.296580	-2.140944	-0.144780
H	3.290175	-3.218990	-0.210338
C	4.482936	-1.462434	-0.096291
H	5.409314	-2.022862	-0.101255
C	0.836884	-2.120446	-0.164582
C	-0.370877	-1.432278	-0.194575
C	-2.829303	-1.479843	-0.274087
C	-1.620172	-2.114741	-0.248137
H	-1.567109	-3.191077	-0.271481
P	-5.556452	-0.013059	-0.801263
C	-4.080480	-2.287723	-0.234120
C	-5.367324	-1.732351	-0.390131
C	-6.527359	-2.511485	-0.311725
C	-6.443728	-3.873442	-0.091971
P	6.124208	0.689738	0.047762
C	4.569057	2.998039	0.036684
C	5.864086	2.445113	0.066455
C	6.913964	4.621399	0.091309
C	7.014393	3.244396	0.099242
O	-0.345799	4.167558	0.057251
O	0.840724	-3.482767	-0.251743
C	-0.440994	4.926364	-1.166166
H	-1.233963	4.539035	-1.807355
H	-0.670875	5.947131	-0.869015
H	0.508695	4.895950	-1.704377
C	0.985941	-4.214621	0.983773
H	1.225010	-5.237586	0.701822
H	1.790231	-3.800105	1.592936
H	0.051731	-4.188897	1.548944

H	-7.498364	-2.045133	-0.423706
H	-7.340879	-4.474631	-0.029772
H	7.803395	5.236503	0.113382
H	7.992643	2.780844	0.134989
C	6.963954	0.156028	1.551273
C	8.044433	-0.728931	1.515148
C	6.470076	0.611746	2.779429
C	8.626620	-1.157383	2.704045
H	8.439446	-1.088286	0.574971
C	7.055874	0.178953	3.959533
H	5.635651	1.301698	2.817771
C	8.131719	-0.706033	3.922000
H	9.464600	-1.841844	2.674512
H	6.673109	0.533455	4.907838
H	8.584664	-1.043524	4.845681
C	-6.891180	0.782637	0.105777
C	-6.733545	1.026074	1.476309
C	-8.063223	1.180959	-0.543782
C	-7.736958	1.671646	2.183580
H	-5.827289	0.725344	1.987497
C	-9.065893	1.823172	0.174409
H	-8.203936	1.000359	-1.601022
C	-8.903646	2.069285	1.532811
H	-7.606172	1.871090	3.239496
H	-9.969813	2.135603	-0.332291
H	-9.684139	2.575329	2.086891
C	4.507849	4.405890	0.030764
C	5.645357	5.195321	0.054894
H	3.557100	4.913854	-0.000175
H	5.538939	6.272918	0.045422
C	-5.184290	-4.449079	0.057252
C	-4.036906	-3.675198	-0.001576
H	-5.094477	-5.512527	0.240181
H	-3.093110	-4.173303	0.156311
C	7.097212	0.207827	-1.391901
H	7.192928	-0.876777	-1.443075
H	6.574215	0.565256	-2.278259
H	8.087623	0.658811	-1.343018
C	-5.859575	0.141781	-2.572329
H	-6.776297	-0.378169	-2.849750
H	-5.926945	1.193142	-2.852232
H	-5.018997	-0.320540	-3.089220

***Trans*-2-O**

C	-3.969847	1.140785	-0.646081
C	-2.897233	0.242203	-0.726830
C	-1.561598	0.760401	-0.652773
C	-1.350920	2.171789	-0.561268
C	-2.464812	3.038261	-0.536853
C	-3.736665	2.527375	-0.567912
C	-0.440933	-0.109030	-0.650307
C	-0.025895	2.670310	-0.477704
C	1.069272	1.816399	-0.431466
C	0.865860	0.405186	-0.523647
C	1.985201	-0.465096	-0.488048
C	3.318169	0.049089	-0.349204
C	3.491486	1.493816	-0.238112
C	2.396874	2.306964	-0.281637
H	-2.305696	4.105388	-0.468230
H	-4.582437	3.202912	-0.530804
H	2.518352	3.376691	-0.179817
C	1.774561	-1.875128	-0.591029
C	4.388084	-0.855335	-0.319710
C	2.886760	-2.743896	-0.569407
H	2.731034	-3.809021	-0.669177
C	4.154277	-2.239797	-0.431788
H	4.995364	-2.922335	-0.406605
C	0.451750	-2.371787	-0.717241
C	-0.644885	-1.519221	-0.756193
C	-3.071367	-1.197468	-0.895424
C	-1.974144	-2.008744	-0.905612
H	-2.095939	-3.074886	-1.040266
P	-5.704142	0.643542	-0.606750
C	-4.405732	-1.758390	-1.072279
C	-5.613850	-1.101787	-0.964711
C	-6.742057	-1.945998	-1.180599
C	-6.384921	-3.228187	-1.461951
S	-4.677675	-3.430523	-1.471835
P	6.119643	-0.380254	-0.114436
C	4.821449	2.065757	-0.072534
C	6.024377	1.396123	0.004249
C	6.790793	3.573509	0.200493
C	7.147248	2.260781	0.164034
S	5.090699	3.780644	0.050637
O	0.168175	4.021344	-0.367437
O	0.261582	-3.720159	-0.857841
C	0.431332	4.727053	-1.595842
H	-0.472023	4.785292	-2.206707

H	0.751814	5.726339	-1.308459
H	1.222843	4.233414	-2.162907
C	-0.006944	-4.455899	0.351266
H	-0.316947	-5.450529	0.037435
H	0.891118	-4.521837	0.969019
H	-0.807270	-3.981468	0.922069
H	-7.767832	-1.608451	-1.134405
H	-7.025736	-4.069266	-1.674411
H	7.428257	4.436283	0.312382
H	8.168431	1.916169	0.246670
C	6.941020	-0.739382	-1.697125
C	6.429587	-0.240229	-2.899061
C	8.098603	-1.519837	-1.710627
C	7.065159	-0.526337	-4.100094
H	5.533116	0.368472	-2.902369
C	8.736544	-1.802203	-2.916293
H	8.494709	-1.903422	-0.779328
C	8.218837	-1.308693	-4.108658
H	6.661226	-0.140753	-5.027989
H	9.634869	-2.406875	-2.922176
H	8.711437	-1.534560	-5.046521
O	6.808709	-1.064538	1.023853
O	-6.596713	1.418168	-1.524035
C	-6.260514	0.794923	1.119441
C	-5.387510	0.664747	2.203272
C	-7.620199	1.018634	1.356801
C	-5.867545	0.765137	3.504605
H	-4.331022	0.491790	2.038412
C	-8.099183	1.105673	2.659370
H	-8.299124	1.133735	0.521478
C	-7.222703	0.983874	3.733585
H	-5.183776	0.672907	4.339215
H	-9.154017	1.278242	2.834965
H	-7.595655	1.061151	4.747630

***Cis*-2-O**

C	-4.191320	1.031072	-0.453401
C	-3.117921	0.135800	-0.540410
C	-1.782747	0.659338	-0.493031
C	-1.575293	2.068463	-0.371279
C	-2.691279	2.929879	-0.300947
C	-3.960847	2.416125	-0.342720
C	-0.658272	-0.201617	-0.562747
C	-0.251304	2.572290	-0.314912

C	0.850705	1.727397	-0.362630
C	0.650236	0.319688	-0.498763
C	1.774717	-0.540769	-0.573390
C	3.110083	-0.018247	-0.516891
C	3.282858	1.421424	-0.351537
C	2.181964	2.223690	-0.276370
H	-2.535154	3.994874	-0.200745
H	-4.806998	3.090552	-0.288091
H	2.303563	3.288601	-0.133881
C	1.567029	-1.948503	-0.708666
C	4.183539	-0.912180	-0.616745
C	2.682589	-2.807641	-0.803165
H	2.526055	-3.870631	-0.922019
C	3.952056	-2.293995	-0.760317
H	4.796978	-2.967134	-0.843906
C	0.243199	-2.453552	-0.754467
C	-0.858868	-1.609709	-0.695028
C	-3.290908	-1.305792	-0.688622
C	-2.190167	-2.107719	-0.766774
H	-2.312435	-3.173605	-0.901020
P	-5.928315	0.544084	-0.499974
C	-4.626092	-1.886122	-0.756538
C	-5.834197	-1.228288	-0.657423
C	-6.963950	-2.094422	-0.745448
C	-6.607550	-3.396953	-0.910708
S	-4.899481	-3.592256	-0.966315
P	5.920513	-0.417997	-0.620607
C	4.618339	2.000340	-0.270516
C	5.827105	1.342310	-0.360921
C	6.598971	3.510373	-0.098943
C	6.956243	2.208203	-0.265222
S	4.890745	3.706001	-0.055429
O	-0.063446	3.917438	-0.145723
O	0.054701	-3.798171	-0.927506
C	0.168153	4.687780	-1.340593
H	-0.749413	4.773477	-1.926411
H	0.490572	5.672192	-1.007560
H	0.948175	4.230102	-1.951551
C	-0.164038	-4.574587	0.265529
H	-0.490196	-5.557274	-0.069071
H	0.759595	-4.663802	0.841277
H	-0.937523	-4.120359	0.887385
H	-7.989536	-1.757478	-0.689812
H	-7.248981	-4.258182	-1.011263

H	7.239818	4.371432	0.006479
H	7.982078	1.872056	-0.321454
C	6.641683	-1.139430	0.884897
C	6.174583	-0.760903	2.147551
C	7.648710	-2.100239	0.783766
C	6.710511	-1.336228	3.292152
H	5.392967	-0.015770	2.241083
C	8.183779	-2.678208	1.933249
H	8.011935	-2.392047	-0.193202
C	7.714657	-2.297233	3.185910
H	6.344041	-1.034604	4.265768
H	8.964749	-3.423830	1.848342
H	8.130502	-2.746035	4.079605
O	6.666760	-0.836284	-1.848335
O	-6.719630	1.232471	-1.566011
C	-6.614986	0.907644	1.142037
C	-6.030881	0.401727	2.307368
C	-7.746662	1.720611	1.232107
C	-6.570068	0.715318	3.548330
H	-5.153848	-0.232368	2.249951
C	-8.286721	2.031113	2.476817
H	-8.197065	2.107121	0.327376
C	-7.696676	1.532185	3.633267
H	-6.112872	0.324220	4.448629
H	-9.165018	2.661407	2.540936
H	-8.114356	1.779501	4.601558

***Trans*-2**

C	-4.331647	0.886495	-0.337134
C	-3.254407	0.004486	-0.498536
C	-1.917144	0.530179	-0.496737
C	-1.702249	1.929744	-0.304817
C	-2.818055	2.778835	-0.139598
C	-4.087429	2.265300	-0.174432
C	-0.795332	-0.321207	-0.680259
C	-0.379459	2.434478	-0.289446
C	0.719308	1.605075	-0.467224
C	0.514881	0.204751	-0.670196
C	1.636689	-0.646661	-0.853629
C	2.973973	-0.120981	-0.851692
C	3.143601	1.317179	-0.675742
C	2.050924	2.108007	-0.471596
H	-2.661690	3.837477	0.014880
H	-4.930060	2.938340	-0.061993

H	2.182073	3.167842	-0.304593
C	1.421890	-2.046229	-1.045573
C	4.051327	-1.002916	-1.013048
C	2.537768	-2.895253	-1.210758
H	2.381458	-3.953886	-1.365355
C	3.807148	-2.381686	-1.175854
H	4.649744	-3.054776	-1.288310
C	0.099079	-2.550962	-1.060917
C	-0.999691	-1.721549	-0.883171
C	-3.424058	-1.433721	-0.674396
C	-2.331357	-2.224509	-0.878696
H	-2.462531	-3.284320	-1.045834
P	-6.096753	0.418550	-0.409851
C	-4.751592	-2.025589	-0.599299
C	-5.951431	-1.379253	-0.404786
C	-7.064918	-2.271378	-0.343435
C	-6.702232	-3.576397	-0.481193
S	-5.006266	-3.748813	-0.703221
P	5.816491	-0.534755	-0.940254
C	4.471081	1.909085	-0.750668
C	5.671029	1.262969	-0.945298
C	6.421331	3.460255	-0.868639
C	6.784312	2.155332	-1.006560
S	4.725396	3.632281	-0.646151
O	-0.181623	3.770279	-0.032416
O	-0.098815	-3.886745	-1.317987
C	-0.044434	4.625769	-1.179142
H	-0.998197	4.730667	-1.701412
H	0.276764	5.594004	-0.799124
H	0.705498	4.236186	-1.870732
C	-0.236011	-4.742321	-0.171335
H	-0.556909	-5.710609	-0.551475
H	0.717676	-4.847017	0.351114
H	-0.986176	-4.352974	0.520137
H	-8.087269	-1.949346	-0.199132
H	-7.335037	-4.449850	-0.471431
H	7.053963	4.333830	-0.878384
H	7.806729	1.833556	-1.150960
C	6.401251	-0.938193	-2.647137
C	5.816977	-0.405977	-3.802279
C	7.458432	-1.841507	-2.772887
C	6.280871	-0.779752	-5.057026
H	4.995928	0.297037	-3.722391
C	7.923378	-2.218201	-4.031077

H	7.919058	-2.254795	-1.883424
C	7.332149	-1.689393	-5.172813
H	5.821424	-0.365277	-5.946301
H	8.742717	-2.921594	-4.116485
H	7.689720	-1.983819	-6.152178
C	-6.681627	0.821804	1.297070
C	-6.097424	0.289481	2.452196
C	-7.738969	1.724923	1.422854
C	-6.561511	0.662940	3.706973
H	-5.276222	-0.413354	2.372264
C	-8.204099	2.101292	2.681070
H	-8.199541	2.138323	0.533411
C	-7.612978	1.572378	3.822816
H	-6.102127	0.248345	4.596224
H	-9.023584	2.804513	2.766483
H	-7.970758	1.866579	4.802166

***Cis*-2**

C	-4.279073	0.881021	-0.493050
C	-3.192614	-0.004967	-0.516185
C	-1.858066	0.526969	-0.472222
C	-1.656770	1.938913	-0.378904
C	-2.782425	2.790661	-0.333793
C	-4.046858	2.269275	-0.408629
C	-0.724868	-0.327729	-0.525767
C	-0.337665	2.452340	-0.339831
C	0.772893	1.620350	-0.396485
C	0.582137	0.206815	-0.498877
C	1.714715	-0.646543	-0.579674
C	3.048911	-0.111654	-0.584104
C	3.208343	1.332807	-0.444602
C	2.102907	2.128426	-0.352727
H	-2.636543	3.858732	-0.247605
H	-4.895378	2.944401	-0.398668
H	2.221155	3.196329	-0.231059
C	1.512998	-2.059168	-0.662863
C	4.132296	-0.992636	-0.710717
C	2.636346	-2.908652	-0.762382
H	2.488290	-3.977418	-0.834946
C	3.898937	-2.380118	-0.800777
H	4.745227	-3.049658	-0.906207
C	0.194801	-2.575077	-0.662922
C	-0.915206	-1.742470	-0.612567
C	-3.350081	-1.454546	-0.588878

C	-2.244105	-2.252219	-0.645568
H	-2.362088	-3.323677	-0.730118
P	-6.034745	0.408304	-0.665496
C	-4.681332	-2.043498	-0.586545
C	-5.890520	-1.387407	-0.566203
C	-7.010242	-2.272725	-0.556204
C	-6.645075	-3.584127	-0.555417
S	-4.935465	-3.770004	-0.582523
P	5.885360	-0.498915	-0.871531
C	4.542208	1.916181	-0.391952
C	5.749351	1.265324	-0.517736
C	6.511303	3.442400	-0.244573
C	6.871141	2.144925	-0.442822
S	4.803435	3.626028	-0.159746
O	-0.159741	3.806012	-0.185171
O	0.015719	-3.931885	-0.789415
C	0.045715	4.562929	-1.390049
H	-0.880919	4.634460	-1.964211
H	0.364155	5.555327	-1.075767
H	0.820544	4.106787	-2.009213
C	-0.174196	-4.663218	0.433891
H	-0.496175	-5.662224	0.145267
H	0.759682	-4.722034	0.997800
H	-0.941310	-4.194104	1.053358
H	-8.039608	-1.940887	-0.547503
H	-7.280946	-4.455319	-0.544469
H	7.150520	4.305206	-0.142615
H	7.898230	1.818452	-0.534526
C	6.613830	-1.185141	0.686072
C	6.199999	-0.768489	1.955461
C	7.591387	-2.176611	0.583802
C	6.751012	-1.337604	3.096455
H	5.443734	0.001264	2.054405
C	8.142774	-2.751714	1.728714
H	7.924241	-2.505156	-0.394171
C	7.722256	-2.332316	2.985129
H	6.420286	-1.006046	4.073619
H	8.898045	-3.522696	1.635358
H	8.148670	-2.775944	3.876469
C	-6.734521	0.852522	0.988435
C	-6.155330	0.438288	2.192767
C	-7.887486	1.639562	1.024505
C	-6.718702	0.809900	3.406465
H	-5.260958	-0.173277	2.183080

C	-8.454929	2.011020	2.242115
H	-8.343729	1.965701	0.097117
C	-7.868931	1.598614	3.432833
H	-6.261997	0.484932	4.333598
H	-9.349303	2.621754	2.257226
H	-8.304771	1.890056	4.380722

***Trans*-2-Me**

C	-4.381921	0.802115	-0.144956
C	-3.258952	-0.071716	-0.349567
C	-1.950521	0.499687	-0.413971
C	-1.767711	1.929018	-0.263552
C	-2.926167	2.754388	-0.050628
C	-4.164696	2.200917	0.003157
C	-0.805268	-0.324000	-0.622337
C	-0.494077	2.460240	-0.318359
C	0.659193	1.657722	-0.531219
C	0.498166	0.244742	-0.686022
C	1.643492	-0.578898	-0.894361
C	2.951978	-0.007499	-0.958246
C	3.074795	1.414190	-0.819088
C	1.941468	2.197606	-0.600211
H	-2.794795	3.819152	0.081078
H	-5.017703	2.850653	0.173207
H	2.052545	3.264798	-0.464692
C	1.460742	-2.008189	-1.045097
C	4.075103	-0.881238	-1.162342
C	2.619299	-2.833424	-1.258116
H	2.487987	-3.898147	-1.390228
C	3.857886	-2.279976	-1.311170
H	4.710945	-2.929712	-1.480974
C	0.187065	-2.539433	-0.990428
C	-0.966230	-1.736989	-0.777153
C	-3.381796	-1.493435	-0.488406
C	-2.248511	-2.276851	-0.707768
H	-2.359647	-3.344067	-0.843067
P	-6.025901	0.242838	-0.095293
C	-4.674289	-2.119135	-0.372906
C	-5.908621	-1.511080	-0.172378
C	-6.997101	-2.433117	-0.068389
C	-6.598137	-3.725290	-0.194016
S	-4.893202	-3.849088	-0.438702
P	5.719206	-0.321993	-1.209784
C	4.367338	2.039916	-0.933638

C	5.601856	1.431917	-1.133122
C	6.291280	3.646136	-1.110693
C	6.690396	2.353995	-1.236192
S	4.586085	3.769868	-0.867844
O	-0.306551	3.816861	-0.091554
O	-0.000491	-3.896013	-1.217521
C	-0.273154	4.639642	-1.263211
H	-1.262872	4.705474	-1.723738
H	0.044539	5.630409	-0.938261
H	0.438102	4.252486	-1.997439
C	-0.033959	-4.719087	-0.046062
H	-0.351940	-5.709695	-0.371216
H	0.955790	-4.785298	0.414347
H	-0.745039	-4.331926	0.688333
H	-8.024324	-2.140543	0.101725
H	-7.201372	-4.618221	-0.154096
H	6.894526	4.539091	-1.149883
H	7.717790	2.061448	-1.405292
C	6.584481	-0.841003	-2.730275
C	6.017048	-0.472031	-3.955364
C	7.753721	-1.605828	-2.720071
C	6.612887	-0.857479	-5.147904
H	5.104165	0.111271	-3.975778
C	8.346736	-1.995224	-3.918202
H	8.209794	-1.907764	-1.787496
C	7.777565	-1.622830	-5.130327
H	6.166540	-0.565218	-6.090172
H	9.250790	-2.590739	-3.900759
H	8.237761	-1.930170	-6.061234
C	-6.888830	0.761689	1.426610
C	-6.320308	0.391143	2.650736
C	-8.057026	1.528112	1.418401
C	-6.914144	0.776540	3.844272
H	-5.408163	-0.193364	2.669581
C	-8.648011	1.917483	2.617537
H	-8.513785	1.831373	0.486596
C	-8.077807	1.543458	3.828677
H	-6.467068	0.482982	4.785798
H	-9.551260	2.514261	2.601658
H	-8.536419	1.850789	4.760370
C	6.734020	-0.946514	0.166925
H	7.745110	-0.538921	0.126924
H	6.252722	-0.627560	1.090928
H	6.778744	-2.036201	0.145144

C	-7.042826	0.867633	-1.470314
H	-8.053841	0.459965	-1.428850
H	-7.087598	1.957310	-1.448172
H	-6.562953	0.548936	-2.395147

***Cis*-2-Me**

C	-4.191120	1.099809	-0.302752
C	-3.096265	0.169003	-0.370426
C	-1.762017	0.675777	-0.295172
C	-1.525572	2.097364	-0.144735
C	-2.658372	2.979276	-0.058873
C	-3.921445	2.488722	-0.143816
C	-0.641968	-0.203564	-0.370711
C	-0.228117	2.566433	-0.086356
C	0.900391	1.709469	-0.185355
C	0.687542	0.302935	-0.328303
C	1.807119	-0.573468	-0.431853
C	3.140235	-0.058589	-0.428098
C	3.316732	1.357794	-0.290445
C	2.206879	2.191138	-0.157336
H	-2.489396	4.038252	0.077580
H	-4.754071	3.182286	-0.079388
H	2.355524	3.253800	-0.024697
C	1.572189	-1.998597	-0.546699
C	4.233308	-0.981380	-0.560733
C	2.705972	-2.878660	-0.640126
H	2.538625	-3.943855	-0.718683
C	3.967462	-2.377060	-0.649583
H	4.800462	-3.069118	-0.724689
C	0.275098	-2.471621	-0.568755
C	-0.853975	-1.611592	-0.501786
C	-3.271638	-1.247294	-0.511990
C	-2.160653	-2.088637	-0.570445
H	-2.307429	-3.152664	-0.696334
P	-5.854294	0.622185	-0.434522
C	-4.596648	-1.805943	-0.589893
C	-5.811533	-1.132094	-0.586989
C	-6.949621	-1.994642	-0.659419
C	-6.605845	-3.307050	-0.717828
S	-4.892975	-3.523568	-0.688565
P	5.898410	-0.485885	-0.591888
C	4.639664	1.926379	-0.302557
C	5.854853	1.265686	-0.439030
C	6.642277	3.441814	-0.299840

C	6.989379	2.136364	-0.438783
S	4.932269	3.641782	-0.166311
O	0.006124	3.917222	0.125190
O	0.043156	-3.829021	-0.736610
C	0.193808	4.700118	-1.059116
H	-0.745948	4.811331	-1.606990
H	0.545411	5.679220	-0.734065
H	0.939119	4.246140	-1.716697
C	-0.147382	-4.570471	0.473927
H	-0.497293	-5.560846	0.182942
H	0.791400	-4.661768	1.027488
H	-0.894379	-4.093842	1.113468
H	-7.974131	-1.648695	-0.661672
H	-7.255640	-4.165510	-0.779089
H	7.289049	4.303986	-0.264302
H	8.012220	1.799680	-0.538017
C	6.857444	-1.215755	0.781593
C	6.510988	-0.815938	2.077814
C	7.839533	-2.193823	0.608818
C	7.135340	-1.383253	3.179754
H	5.745537	-0.063277	2.226806
C	8.460493	-2.767354	1.717521
H	8.126644	-2.522259	-0.381147
C	8.110212	-2.363601	3.000836
H	6.857512	-1.062279	4.176144
H	9.216756	-3.529204	1.574088
H	8.592887	-2.811310	3.860532
C	-6.850958	1.071339	1.030501
C	-6.225719	1.043229	2.281005
C	-8.223523	1.331432	0.957198
C	-6.961666	1.258969	3.439538
H	-5.162091	0.847529	2.346423
C	-8.959946	1.533897	2.120744
H	-8.729845	1.372939	0.002044
C	-8.331413	1.495262	3.361239
H	-6.466263	1.237704	4.402376
H	-10.023333	1.728538	2.055582
H	-8.905773	1.654541	4.265418
C	6.754010	-0.965810	-2.123722
H	6.684896	-2.043701	-2.276697
H	6.248538	-0.458460	-2.944377
H	7.803191	-0.668101	-2.099502
C	-6.710345	1.376171	-1.854799
H	-7.713085	0.965472	-1.976664

H	-6.776856	2.456378	-1.719204
H	-6.121770	1.155459	-2.744793

***Cis*-2-O(BCF)$_2$**

C	-4.055489	0.555530	-1.113006
C	-2.908149	-0.243957	-1.016821
C	-1.633058	0.402261	-1.090379
C	-1.565189	1.809981	-1.291790
C	-2.747877	2.577834	-1.319057
C	-3.966732	1.959814	-1.221107
C	-0.427789	-0.347408	-1.016960
C	-0.296605	2.430591	-1.469124
C	0.875300	1.704870	-1.346556
C	0.817439	0.296910	-1.113493
C	2.023610	-0.448522	-1.031476
C	3.296520	0.198071	-1.156894
C	3.322397	1.651825	-1.322470
C	2.153627	2.338754	-1.433807
H	-2.678228	3.659070	-1.429107
H	-4.880235	2.553745	-1.259980
H	2.158071	3.420771	-1.566705
C	1.956135	-1.859526	-0.850534
C	4.449150	-0.598705	-1.118051
C	3.147681	-2.616029	-0.796566
H	3.088815	-3.696751	-0.676118
C	4.362968	-1.995467	-0.926287
H	5.273699	-2.591782	-0.900809
C	0.684739	-2.495419	-0.754848
C	-0.489017	-1.764354	-0.846334
C	-2.939680	-1.701971	-0.896055
C	-1.771912	-2.397478	-0.808855
H	-1.781766	-3.485162	-0.739680
P	-5.696549	-0.128345	-1.292530
C	-4.217996	-2.411483	-0.916507
C	-5.476930	-1.865809	-1.069766
C	-6.526891	-2.835432	-1.088262
C	-6.046377	-4.100943	-0.946001
S	-4.332573	-4.133726	-0.790301
P	6.077315	0.080324	-1.438349
C	4.594499	2.370545	-1.328520
C	5.860415	1.825306	-1.310766
C	6.416116	4.066772	-1.277860
C	6.907584	2.798145	-1.282284
S	4.693954	4.099356	-1.303557

O	-0.253350	3.754062	-1.762326
O	0.636661	-3.842468	-0.599218
C	-0.334025	4.051816	-3.157925
H	-1.229122	3.593277	-3.605356
H	-0.388187	5.142809	-3.250027
H	0.559604	3.678481	-3.682866
C	0.636701	-4.314840	0.745892
H	0.619381	-5.410057	0.699014
H	1.541540	-3.984700	1.279858
H	-0.253746	-3.955625	1.285754
H	-7.582578	-2.590876	-1.207224
H	-6.608137	-5.033694	-0.922854
H	6.973116	5.002494	-1.260943
H	7.969546	2.553041	-1.275627
C	7.229418	-0.506523	-0.192605
C	6.813100	-0.645366	1.135614
C	8.548681	-0.792972	-0.554109
C	7.716728	-1.086277	2.096080
H	5.783080	-0.418766	1.420009
C	9.446269	-1.235433	0.412760
H	8.867867	-0.675620	-1.589833
C	9.030199	-1.383975	1.734194
H	7.392959	-1.202721	3.132060
H	10.475067	-1.467105	0.130673
H	9.735507	-1.734674	2.490540
O	6.688637	-0.265808	-2.816063
O	-6.347576	0.212456	-2.664015
C	-6.813436	0.521254	-0.052649
C	-6.376298	0.632614	1.271381
C	-8.119184	0.870635	-0.406855
C	-7.250989	1.105252	2.243033
H	-5.353997	0.357935	1.542627
C	-8.988355	1.336872	0.575163
H	-8.440321	0.785811	-1.444778
C	-8.555131	1.456034	1.894150
H	-6.913671	1.202856	3.276593
H	-10.009624	1.613537	0.306361
H	-9.239765	1.827572	2.659583
B	6.665016	-1.038433	-4.130467
C	7.980527	-2.011915	-4.010882
C	9.149151	-1.912961	-4.758592
F	9.264858	-1.045475	-5.768513
C	10.284664	-2.684097	-4.510257
F	11.373737	-2.536366	-5.257246

C	10.281351	-3.599558	-3.470565
F	11.355666	-4.333904	-3.215943
C	9.137701	-3.735513	-2.693233
F	9.116548	-4.600926	-1.685538
C	8.037409	-2.943867	-2.978153
F	6.976739	-3.098567	-2.170827
C	6.739158	0.005531	-5.383590
C	6.829927	1.388386	-5.308315
F	6.933471	2.021417	-4.135788
C	6.818866	2.211440	-6.432599
F	6.903691	3.532235	-6.299985
C	6.717109	1.650260	-7.695579
F	6.708221	2.423155	-8.774693
C	6.631676	0.268605	-7.820064
F	6.545059	-0.285571	-9.025428
C	6.640190	-0.509868	-6.672570
F	6.577428	-1.837745	-6.842262
C	5.217223	-1.802862	-4.233928
C	4.060969	-1.031723	-4.153503
F	4.158273	0.301650	-4.032505
C	2.774331	-1.552579	-4.138332
F	1.722081	-0.750239	-4.018746
C	2.610427	-2.929209	-4.214525
F	1.404982	-3.474911	-4.150114
C	3.729641	-3.738926	-4.346131
F	3.579476	-5.055844	-4.440760
C	4.997632	-3.167856	-4.373952
F	6.014303	-4.019027	-4.532602
B	-5.906998	0.499705	-4.099847
C	-4.402679	-0.135337	-4.250644
C	-3.212712	0.583085	-4.318330
F	-3.209793	1.919974	-4.390463
C	-1.954909	-0.011108	-4.253470
F	-0.852287	0.733834	-4.247492
C	-1.857893	-1.385814	-4.115384
F	-0.680739	-1.970849	-3.976780
C	-3.015463	-2.148295	-4.061974
F	-2.930321	-3.459978	-3.871265
C	-4.245571	-1.515336	-4.132758
F	-5.320790	-2.305014	-4.034783
C	-5.989231	2.105855	-4.399573
C	-6.472650	3.089331	-3.547696
F	-6.858740	2.809661	-2.293763
C	-6.574077	4.431588	-3.907026

F	-7.039137	5.326111	-3.040284
C	-6.190706	4.830889	-5.176808
F	-6.282719	6.104117	-5.538306
C	-5.701386	3.882040	-6.067671
F	-5.317260	4.253260	-7.284160
C	-5.626973	2.557252	-5.666282
F	-5.149970	1.679831	-6.558681
C	-7.043429	-0.207793	-5.045738
C	-8.390007	-0.016606	-4.745323
F	-8.751725	0.779032	-3.729530
C	-9.430080	-0.605917	-5.453887
F	-10.695942	-0.402338	-5.102194
C	-9.135661	-1.401265	-6.552588
F	-10.112051	-1.978003	-7.241193
C	-7.810263	-1.586308	-6.919142
F	-7.513834	-2.335544	-7.976591
C	-6.801091	-0.982246	-6.175362
F	-5.554675	-1.202749	-6.604379

9. References

S1 J.-B. Giguère, Q. Verolet and J.-F. Morin, *Chem. Eur. J.*, 2013, **19**, 372–381.

S2 F. Lirette, C. Aumaitre, C.-É. Fecteau, P. A. Johnson and J.-F. Morin, *ACS Omega*, 2019, **4**, 14742–14749.

S3 R. E. H. Kuveke, L. Barwise, Y. Van Ingen, K. Vashisth, N. Roberts, S. S. Chitnis, J. L. Dutton, C. D. Martin and R. L. Melen, *ACS Cent. Sci.*, 2022, **8**, 855–863.

S4 C. Reus, M. Stolar, J. Vanderkley, J. Nebauer and T. Baumgartner, *J. Am. Chem. Soc.*, 2015, **137**, 11710–11717.

S5 A. D. Becke, *The Journal of Chemical Physics*, 1992, **96**, 2155–2160.

S6 F. Weigend and R. Ahlrichs, *Phys. Chem. Chem. Phys.*, 2005, **7**, 3297.

S7 J. Tomasi, B. Mennucci and R. Cammi, *Chem. Rev.*, 2005, **105**, 2999–3094.

S8 P. V. R. Schleyer, C. Maerker, A. Dransfeld, H. Jiao and N. J. R. Van Eikema Hommes, *J. Am. Chem. Soc.*, 1996, **118**, 6317–6318.

S9 J.-D. Chai and M. Head-Gordon, *The Journal of Chemical Physics*, 2009, **131**, 174105.

S10 E. Paenurk and R. Gershoni-Poranne, *Phys. Chem. Chem. Phys.*, 2022, **24**, 8631–8644.

S11 J. R. Gaffen, J. N. Bentley, L. C. Torres, C. Chu, T. Baumgartner and C. B. Caputo, *Chem*, 2019, **5**, 1567–1583.

S12 J. R. Lakowicz, Principles of Fluorescence Spectroscopy, Springer (third edition), 2006.
